# Exploring the interplay between kidney function and urinary metabolites in young adults: the African-PREDICT study

**DOI:** 10.1007/s00726-024-03412-7

**Published:** 2024-08-29

**Authors:** Wessel L. du Toit, Ruan Kruger, Lebo F. Gafane-Matemane, Aletta E. Schutte, Roan Louw, Catharina M. C. Mels

**Affiliations:** 1 Hypertension in Africa Research Team (HART), North-West University, Private Bag X6001, Potchefstroom, 2520 South Africa; 2 Cardiovascular Pathophysiology and Genomics Research Unit (CPGRU), University of the Witwatersrand, Johannesburg, South Africa; 3 MRC Research Unit for Hypertension and Cardiovascular Disease, North-West University, Potchefstroom, South Africa; 4 School of Population Health, University of New South Wales, The George Institute for Global Health, Sydney, Australia; 5 Human Metabolomics, North-West University, Potchefstroom Campus, Potchefstroom, South Africa

**Keywords:** Cardiovascular disease, Kidney disease, Estimated glomerular filtration rate, Kidney function, Metabolomics, Risk factors

## Abstract

The exposure to modifiable risk factors at young ages have been linked to premature fatal and non-fatal cardiovascular and kidney outcomes. The use of urinary metabolomics has shown strong predictability of kidney function and cardiovascular disease (CVD). We therefore determined the associations between estimated glomerular filtration rate (eGFR) and urinary metabolites in young adults with and without CVD risk factors. Apparently healthy Black and White sexes were included (aged 20–30 years) and categorised by the presence or absence of risk factors, i.e., obesity, physical inactivity, smoking, excessive alcohol intake, masked hypertension, hyperglycemia, dyslipidemia and low socio-economic status, forming the CVD risk group (N = 1036), CVD risk clusters (i.e. presenting with 1 CVD risk factor (N = 344), 2 CVD risk factors (N = 360) and 3 + CVD risk factors (N = 332)) and the control group (N = 166). eGFR was calculated with CKD-EPI equations. A targeted metabolomics approach using liquid chromatography-tandem mass spectrometry was used to measure amino acids and acylcarnitines. Lower cystatin C-based eGFR were indicated in the CVD risk group, 2 and 3 + CVD risk clusters compared to the control group (all P ≤ 0.033). In the CVD risk group, eGFR associated positively with histidine, lysine, asparagine, glycine, serine, glutamine, dimethylglycine, threonine, alanine, creatine, cystine, methionine, tyrosine, pyroglutamic acid, leucine/isoleucine, aspartic acid, tryptophan, glutamic acid, free carnitine, acetylcarnitine, propionylcarnitine, isovalerylcarnitine, octanoylcarnitine and decanoylcarnitine (all P ≤ 0.044), with similar results found in the CVD risk clusters, particularly the 2 CVD risk cluster. eGFR was positively associated with metabolites linked to aromatic amino acid and branched-chain amino acid metabolism, energy metabolism and oxidative stress. These findings may indicate altered reabsorption of these metabolites or altered metabolic regulation to preserve renal health in the setting of CVD risk factors at this young age without established CVD.

**Supplementary Information:**

The online version contains supplementary material available at 10.1007/s00726-024-03412-7.

## Introduction

Kidney disease, a rapidly growing global health problem, is closely associated with the development of cardiovascular disease (CVD), another major worldwide health challenge (Ulasi et al. 2022; World Health Organisation 2022; Deferrari et al. 2021). The cardiovascular system and kidneys maintain a close interconnection, mutually influencing each other to uphold overall physiological balance and homeostasis (Deferrari et al. 2021). Deviations from this delicate balance can result in complications affecting both the cardiovascular system and kidneys (Deferrari et al. 2021). Thus, highlighting the importance to detect changes in kidney function at early stages as these changes may have adverse effects on cardiovascular and kidney health (World Health Organisation 2022; Deferrari et al. 2021; Jacobs et al. 2022).

Early exposure to CVD risk factors (Jacobs et al. 2022; Cercato and Fonseca 2019; Lavie et al. 2019; Banks et al. 2019; Piano 2017; Kjeldsen 2018; Matheus et al. 2013; Nelson 2013; Rosengren et al. 2019; Schultz et al. 2018) contributes to premature changes in the cardiovascular system which may also affect the kidneys (Kovesdy et al. 2017; Volaklis et al. 2021; Habas et al. 2022; Amorim et al. 2019; Mikolasevic et al. 2017; Grant et al. 2023; Xia et al. 2017; Pan et al. 2018; Kazancioğlu 2013). These risk factors are also associated with kidney disease and include risk factors such as obesity and physical inactivity to elevated blood pressure (BP), hyperglycemia, dyslipidemia, and low socio-economic status (SES), along with tobacco and alcohol use, or combinations thereof (Kovesdy et al. 2017; Volaklis et al. 2021; Habas et al. 2022; Amorim et al. 2019; Mikolasevic et al. 2017; Grant et al. 2023; Xia et al. 2017; Pan et al. 2018; Kazancioğlu 2013). The presence of CVD risk factors and their impact on kidney function may precipitate metabolic changes even before the onset of established kidney and/or cardiovascular complications (Danilova et al. 2023; McGarrah et al. 2018). To uncover potential early metabolic shifts, we utilised metabolomics, a potent high-throughput tool enabling the simultaneous quantification of multiple metabolites (Danilova et al. 2023; McGarrah et al. 2018).

As part of the African Prospective study on Early Detection and Identification of Cardiovascular disease and Hypertension (African-PREDICT) cohort (aged 20–30 years), specific urinary metabolomic profiles and pathways associated with cardiovascular markers (markers of arterial stiffness and cardiac structural alterations) in the presence of CVD risk factors have been identified (Mels et al. 2019; De Beer et al. 2020; du Toit et al. 2022, 2023a, b). Among these, altered aromatic amino acid (AAA) and branched-chain amino acid metabolism (BCAA), energetics, and oxidative stress were found to be negatively associated with markers of arterial stiffness and positively associated with cardiac structural alterations (du Toit et al. 2022, 2023a, b). From our previous results, it remains uncertain whether the identified metabolic pathways associated with the cardiovascular markers can be translated to early metabolic alterations linked with changes in kidney function in the presence of CVD risk factors.

Therefore, our objective was to explore the links between kidney function (assessed through estimated glomerular filtration rate (eGFR)) and urinary metabolites in young apparently healthy adults, categorised based on the presence or absence of CVD risk factors (obesity, physical inactivity, smoking, excessive alcohol intake, masked hypertension, hyperglycemia, dyslipidemia and low SES). Uncovering these early metabolic changes associated with kidney function in young adults with CVD risk factors has the potential to unveil biomarkers or pathways that are normally masked by factors such as disease and advanced age.

## Methods

### Study design and population

This research forms part of the African-PREDICT study, which is aimed at investigating early CVD-related pathophysiology by studying apparently healthy Black and White adults aged 20–30 years longitudinally. Detailed information about this study has been previously published (Schutte et al. 2019). In short, participants were recruited on a voluntary basis from the North-West Province of South Africa. During the screening phase, individuals were included if they were normotensive (clinic BP < 140/90 mmHg) (Mancia et al. 2013), uninfected with the human immunodeficiency virus, not diagnosed with chronic diseases or using medication for chronic conditions (self-reported), and not pregnant or lactating (self-reported).

**Fig. 1:**
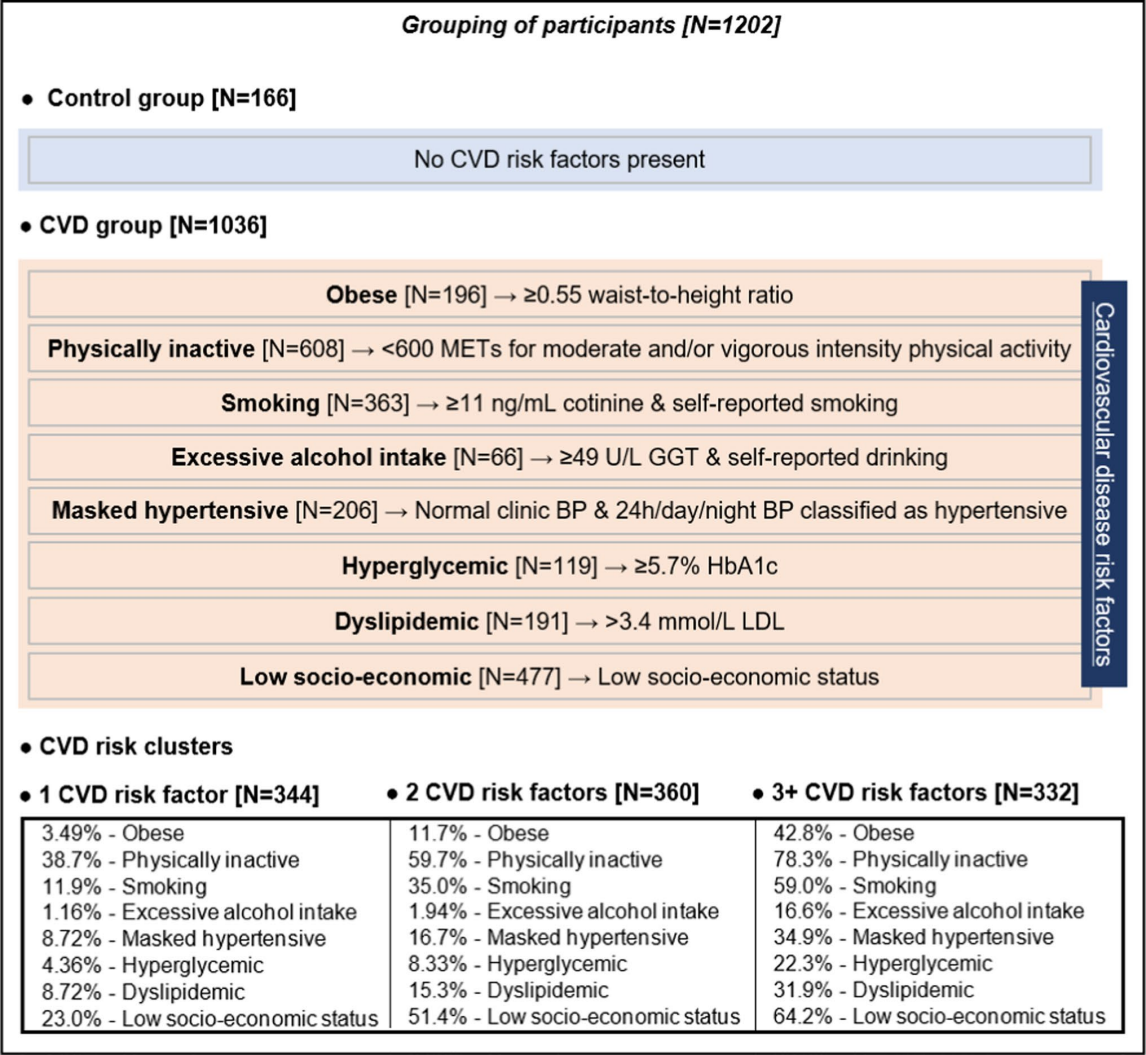
Grouping of participants according to the presence/absence of cardiovascular disease risk factors. Cardiovascular disease risk group criteria and sources: Obese (Yoo 2016; Amirabdollahian and Haghighatdoost 2018)-≥ 0.55 waist-to-height ratio; Physically inactive (Keating et al. 2019; World Health Organisation. 2023)-< 600 METs for moderate and/or vigorous intensity physical activity; Smoking (Raja et al. 2016; Kim 2016)-≥ 11 ng/mL cotinine & self-reported smoking; Excessive alcohol intake (Agarwal et al. 2016; Jastrzebska et al. 2016; Puukka et al. 2006)-≥ 49 U/L GGT & self-reported drinking; Masked hypertensive (Anstey et al. 2018)-normal clinic BP & 24 h/day/night BP classified as hypertensive; Hyperglycemic (Sherwani et al. 2016)-≥ 5.7% HbA1c; Dyslipidemic (Nelson 2013; Pagana et al. 2020)-> 3.4 mmol/L LDL; Low socio-economic (Patro et al. 2012)-low SES. *CVD* cardiovascular disease, *METs* metabolic equivalents, *GGT* gamma-glutamyl transferase, *BP* blood pressure, *HbA1c* glycated haemoglobin, *LDL* low density lipoprotein cholesterol

Approval for this study was obtained from the Health Research Ethics Committee of the North-West University (NWU-00411-20-A1) and adhered to the principles outlined in the Declaration of Helsinki. All participants provided written informed consent. The full baseline cohort of 1202 young adults were analysed cross-sectionally, with stratification into groups based on CVD risk factor criteria (obesity, physical inactivity, smoking, excessive alcohol intake, masked hypertension, hyperglycemia, dyslipidemia and low SES) (Fig. 1).

### Questionnaire data

Questionnaire data, previously detailed (du Toit et al. 2022, 2023a, b), included age, sex, ethnicity, education level, employment information, household income, medication use, smoking and alcohol use from the Demographic Questionnaire; SES and SES score using Kuppuswamy’s Socioeconomic Status Scale 2010 adapted for a South African environment (Patro et al. 2012); sedentary behaviour, moderate and vigorous intensity physical activity (metabolic equivalents) from the Global Physical Activity Questionnaire (Keating et al. 2019; World Health Organisation. 2023); and protein intake from 24h dietary recall questionnaire (Steinfeldt et al. 2013).

### Anthropometric measurements

Anthropometric measurements, following the International Society for the Advancement of Kinanthropometry guidelines (International Society for the Advancement of Kinanthropometry 2001), included height (SECA 213 Portable Stadiometer (SECA, Hamburg, Germany)), weight (SECA 813 Electronic Scales (SECA, Hamburg, Germany)), and waist circumference (Lufkin Steel Anthropometric Tape (W606 PM; Lufkin, Apex, USA)). Subsequently, body mass index and waist-to-height ratio were calculated.

### Cardiovascular measurements

Blood pressure measurements, as previously detailed (du Toit et al. 2022, 2023a, b), included clinic BP taken using the Dinamap Procare 100 Vital Signs Monitor (GE Medical Systems, Milwaukee, USA) (Reinders et al. 2006) and ambulatory BP taken using the Card(X) plore apparatus (Meditech, Budapest, Hungary). The device measured BP in 30 min intervals during daytime (6 a.m–10 p.m.) and hourly during the night (10 p.m–6 a.m.). The mean successful inflation rate over the 24h period was 88%.

### Kidney function measurements

Kidney function was evaluated by measuring both cystatin C and creatinine levels in serum (Cobas Integra® 400 plus (Roche, Basel, Switzerland)), which was subsequently used to calculate eGFR using the Chronic Kidney Disease Epidemiology Collaboration (CKD-EPI) equations (without ethnicity) (Levey and Stevens 2010). Evidence suggests that cystatin C is a superior measure compared to creatinine-based eGFR (limitations in accuracy and sensitivity), however the latter is more commonly used in clinical practice (Spencer et al. 2023). As such, for comprehensive insights, we have included creatinine-based eGFR in supplementary analysis, however it should be noted that for the metabolomics data, urinary creatinine was used to obtain a predetermined urine volume.

### Biochemical analyses

A registered nurse collected blood and spot urine samples from fasting participants. The biological samples were immediately processed, aliquoted into cryovials, and stored at -80ºC until analysis. Biochemical variables, including gamma-glutamyl transferase (GGT), lipid profile (total cholesterol, high-density lipoprotein cholesterol, low-density lipoprotein cholesterol (LDL), and triglycerides), C-reactive protein (all serum samples), glucose levels (sodium fluoride plasma samples), glycated haemoglobin (HbA1c) (EDTA whole blood samples), albumin and creatinine (all spot urine samples) were analysed using the Cobas Integra® 400 plus (Roche, Basel, Switzerland). Cotinine was analysed with the Immulite apparatus (Siemens, Erlangen, Germany), creatine kinase (MB isoenzyme) with the Cobas e411 (Roche, Basel, Switzerland), and serum peroxides as a measure of reactive oxygen species (ROS) using the Synergy H4 hybrid microplate reader (BioTek, Winooski, VT, USA) (Hayashi et al. 2007) (all serum samples).

Metabolomics data (30 amino acids and 9 acylcarnitines) (urine samples) were analysed using a liquid chromatography-tandem mass spectrometry method on an Agilent^©^ system (1200 series LC front end coupled to a 6410 series triple quadrupole mass analyser) with an electrospray ionisation source operating in positive ionisation mode (du Toit et al. 2022). Randomised urine samples were prepared, which included the addition of an isotope mixture added to a predetermined urine volume (corresponding to 0.25 µmoles creatinine, to compensate for variation in urine concentrations) and analysed in batches of 20 samples. Each batch included 3 quality control urine samples and an additional in-house standard mixture (comprising all analysed metabolites to monitor data integrity). Separation of metabolites utilised a Zorbax SB-Aq 80Å StableBond column (Agilent^©^, 2.1 mm × 100 mm × 1.8 μ; cat# 828,700–914) and a Zorbax Eclipse Plus C18 guard column (Agilent^©^, 2.1 mm x 5 mm, 1.8 μm, cat# 821,725–901) with specific run order times and parameters. For data preprocessing, a peak intensity filter removed features with areas below the limit of quantification (LOQ cutoff of area < 750). The metabolomics data were then normalised to the added isotope internal standards. Individual inspection of spectral data matrices for each batch ensured good data quality. Overall, the data demonstrated high quality, with no visible batch effects. Detailed information about this method has been previously published (du Toit et al. 2022).

### Statistical analyses

Statistical analyses were conducted using IBM® SPSS® version 29 (IBM Corporation, Armonk, New York). Variables underwent normality testing, with logarithmic transformation applied to skewed variables. Logged variables included albumin, albumin/creatinine ratio, physical activity, cotinine, GGT, triglycerides, creatine kinase, C-reactive protein, ROS, protein intake, and the metabolomics data. Results are presented as mean with 95% confidence intervals (normally distributed variables), or geometric mean with 95% confidence intervals (logarithmically transformed variables). Participants were grouped based on the presence or absence of CVD risk factor(s) into the CVD risk group, CVD risk clusters and the control group (Fig. 1). Characteristics between the control, CVD risk group and CVD risk clusters were compared using the Chi-square test for categorical variables and ANCOVA (controlled for sex and ethnicity) for continuous variables. Metabolomics data were further adjusted for protein intake, and P-values for comparing metabolomics data between the control, CVD risk groups and CVD risk clusters were adjusted for multiple comparisons using the Benjamini–Hochberg procedure (q-value). Multivariable adjusted regression analyses were performed to assess associations of eGFR (cystatin C-based) with the metabolomics data in the control, CVD risk group and CVD risk clusters. The fully adjusted model included age, sex, ethnicity, protein intake, waist-to-height ratio, physical activity, cotinine, GGT, 24-h systolic BP, HbA1c, LDL, and SES score. Moreover, we also conducted an additional analysis using the same procedure outlined above but focusing on creatinine-based eGFR. The data underlying this article is available in the article and in its online supplementary material.

## Results

The demographics, cardiovascular and kidney risk factors and kidney measurements between the control, CVD risk group and CVD risk clusters are shown in Table 1. As expected, the CVD risk group had a worse CVD risk factor profile (such as higher anthropometry, smoking and alcohol use, masked hypertension, BP, glucose, cholesterol and lower physical activity and SES (all P ≤ 0.003)) compared to the control group. On the other hand, the 1 CVD risk cluster showed higher smoking and alcohol use, masked hypertension, cholesterol and lower SES, compared to the control group (all P ≤ 0.042). Compared to the CVD risk group, similar results were found in the 2 and 3 + CVD risk clusters, which increased or decreased respectively as the risk factors increased (all P ≤ 0.027). The metabolomics comparison between the control, CVD risk group and CVD risk clusters have already been published elsewhere (du Toit et al. 2022, 2023a, b), but have been included as a supplementary table (Supplementary Table 1) (refer to du Toit et al. (2022) for the metabolomics comparison for the individual CVD risk factors). In short, the metabolomics comparison indicated higher creatine and tyrosine in the CVD risk group, higher decanoylcarnitine in the 1 CVD risk cluster, higher histidine and alanine in the 2 CVD risk cluster and higher serine, alanine, creatine, cystine, methionine, tyrosine, leucine/isoleucine, phenylalanine, hexanoylcarnitine and lower acetylcarnitine in the 3 + CVD risk cluster compared to the control group (all P ≤ 0.049). However, after performing the Benjamini–Hochberg adjustment only alanine and tyrosine in the 3 + CVD risk cluster remained significant (all q ≤ 0.039). Kidney function markers revealed lower cystatin C-based eGFR in the CVD risk group compared to the control group (P ≤ 0.005), with no difference in creatinine-based eGFR between the groups. Similar results were found in the 2 and 3 + CVD risk clusters, which decreased as the risk factors increased (P ≤ 0.033). Biochemical markers indicating energy metabolism (creatine kinase) were lower and oxidative stress and inflammation (ROS and C-reactive protein) were higher in the CVD risk group compared to the control group (all P ≤ 0.009), with similar results found in the 2 and 3 + CVD risk clusters, which decreased or increased respectively as the risk factors increased (all P ≤ 0.038).

**Table 1:** Comparisons of demographics, cardiovascular and kidney risk factors and kidney measurements between control, cardiovascular disease risk group and cardiovascular disease risk clusters

	Control group (N = 166)	CVD risk group (N = 1036)	1 CVD risk factor (N = 344)	2 CVD risk factors (N = 360)	3 + CVD risk factors (N = 332)
Age, years	24.8 (24.3; 25.3)	24.5 (24.3; 24.7)	24.5 (24.2; 24.8)	24.2 (23.9; 24.5)	24.9 (24.5; 25.2)
Sex, female, n (%)	108 (65.1)	516 (49.8)**	195 (56.7)	169 (46.9)**	152 (45.8)**
Ethnicity, Black, n (%)	39 (23.5)	567 (54.7)**	154 (44.8)**	205 (56.9)**	208 (62.7)**
Cardiovascular/kidney disease risk factors					
Weight, kg	65.4 (62.9; 67.9)	72.2 (71.2; 73.1)**	67.5 (66.3; 68.7)	70.5 (69.0; 71.9)**	80.7 (78.7; 82.7)**
Waist circumference, cm	74.7 (72.9; 76.5)	81.0 (80.3; 81.7)**	76.7 (75.9; 77.6)	79.6 (78.6; 80.6)**	88.0 (86.6; 89.4)**
Height, m	1.69 (1.68; 1.70)	1.68 (1.68; 1.69)	1.69 (1.68; 1.70)	1.69 (1.68; 1.70)	1.68 (1.67; 1.69)
Body mass index, kg/m^2^	23.0 (22.2; 23.9)	25.4 (25.1; 25.7)**	23.6 (23.2; 24.0)	24.6 (24.1; 25.0)**	28.7 (28.0; 29.3)**
Waist-to-height ratio	0.44 (0.43; 0.45)	0.48 (0.48; 0.49)**	0.45 (0.45; 0.46)	0.47 (0.47; 0.48)**	0.53 (0.52; 0.53)**
Physical act, kCal/kg/day	271 (240; 309)	206 (195; 219)**	239 (223; 256)	206 (189; 224)**	176 (160; 193)**
Cotinine, ng/ml	1.15 (0.83; 1.58)	4.32 (3.80; 4.90)**	1.65 (1.45; 1.88)**	4.01 (3.29; 4.90)**	12.6 (9.99; 15.9)**
Self-reported smoking, n (%)	0 (0)	286 (27.6)**	28 (8.16)**	100 (27.8)**	158 (47.6)**
GGT, U/L	13.1 (12.0; 14.5)	19.2 (18.6; 20.0)**	15.2 (14.4; 16.1)**	17.5 (16.5; 18.6)**	25.3 (23.6; 27.1)**
Self-reported alcohol use, n (%)	69 (41.6)	597 (58.1)**	174 (51.2)*	216 (60.3)**	207 (62.7)**
Masked hypertensive, n (%)	0 (0)	206 (20.1)**	30 (8.77)**	60 (17.1)**	116 (35.4)**
24 h Systolic BP, mmHg	114 (113; 115)	117 (117; 118)**	115 (114; 115)	117 (116; 117)**	121 (120; 122)**
24 h Diastolic BP, mmHg	67.0 (66.1; 68.0)	69.0 (68.6; 69.3)**	67.7 (67.1; 68.3)	68.2 (67.6; 68.8)*	70.9 (70.3; 71.6)**
HbA1c, (%)	5.26 (5.21; 5.30)	5.33 (5.31; 5.35)*	5.27 (5.24; 5.29)	5.29 (5.26; 5.32)*	5.40 (5.37; 5.44)**
Glucose, mmol/L	3.85 (3.69; 4.02)	4.13 (4.07; 4.19)*	3.98 (3.87; 4.09)	4.14 (4.03; 4.25)*	4.36 (4.25; 4.48)**
Total cholesterol, mmol/L	3.29 (3.12; 3.47)	3.83 (3.76; 3.90)**	3.64 (3.53; 3.75)*	3.77 (3.65; 3.88)**	4.26 (4.13; 4.38)**
HDL cholesterol, mmol/L	1.17 (1.11; 1.23)	1.16 (1.13; 1.18)	1.22 (1.17; 1.26)	1.16 (1.11; 1.20)	1.12 (1.08; 1.16)
LDL cholesterol, mmol/L	2.03 (1.88; 2.17)	2.51 (2.45; 2.57)**	2.29 (2.21; 2.37)*	2.43 (2.34; 2.52)**	2.93 (2.82; 3.04)**
Triglycerides, mmol/L	0.56 (0.52; 0.62)	0.75 (0.72; 0.78)**	0.64 (0.61; 0.67)*	0.73 (0.70; 0.77)**	0.95 (0.90; 1.00)**
Creatine kinase, ng/ml	1.75 (1.62; 1.91)	1.52 (1.48; 1.58)*	1.48 (1.39; 1.57)	1.47 (1.39; 1.56)*	1.49 (1.40; 1.58)**
C-reactive protein, mg/L	0.59 (0.47; 0.74)	0.95 (0.87; 1.05)**	0.71 (0.62; 0.83)	0.79 (0.68; 0.91)*	1.61 (1.38; 1.89)**
ROS, mg/L H_2_O_2_	34.3 (30.9; 38.0)	39.4 (38.0; 40.7)*	38.4 (36.0; 40.8)	38.3 (35.8; 40.9)	41.9 (39.2; 44.8)**
SES score	23.5 (22.7; 24.3)	20.2 (19.9; 20.5)**	22.5 (22.0; 23.0)**	20.2 (19.6; 20.7)**	18.5 (17.9; 19.1)**
Protein intake, g	69.7 (64.6; 74.1)	66.5 (64.6; 67.6)	67.6 (64.6; 70.7)	66.4 (63.3; 69.6)	66.7 (63.3; 70.3)
Kidney measurements					
eGFR (Cystatin-C), ml/min/1.73m^2^	128 (125; 132)	123 (122; 124)*	126 (123; 128)	124 (122; 126)*	117 (115; 120)**
eGFR (Creatinine), ml/min/1.73m^2^	120 (117; 123)	119 (118; 121)	119 (117; 121)	119 (117; 121)	119 (117; 121)
Cystatin-C, mg/L	0.67 (0.64; 0.70)	0.71 (0.70; 0.72)*	0.68 (0.66; 0.70)	0.71 (0.69; 0.72)*	0.76 (0.74; 0.78)**
Creatinine, μmol/L	65.5 (63.1; 67.9)	66.5 (65.5; 67.4)	65.7 (63.9; 67.4)	66.5 (64.8; 68.3)	66.8 (65.2; 68.5)
Albumin (urine), mg/L	6.17 (5.50; 6.92)	5.33 (5.13; 5.50)*	5.27 (4.89; 5.67)*	5.40 (5.03; 5.79)	5.40 (4.96; 5.86)*
Creatinine (urine), mmol/L	13.7 (12.6; 14.9)	13.0 (12.6; 13.5)	13.0 (12.2; 13.8)	13.1 (12.3; 13.9)	13.0 (12.1; 13.8)
Albumin/creatinine (urine), mg/mmol	0.53 (0.47; 0.62)	0.50 (0.47; 0.52)	0.50 (0.46; 0.55)	0.50 (0.46; 0.54)	0.50 (0.45; 0.56)

Tests used: Chi-square tests and ANCOVAs (adjusted for sex and ethnicity). Data are presented as mean or geometric mean with 95% confidence intervals. Bold values denote P ≤ 0.05; *P ≤ 0.05; **P ≤ 0.001. Cardiovascular disease risk group criteria: Obese-≥ 0.55 waist-to-height ratio; Physically inactive-< 600 METs for moderate and/or vigorous intensity physical activity; Smoking-≥ 11 ng/mL cotinine & self-reported smoking; Excessive alcohol intake-≥ 49 U/L GGT & self-reported drinking; Masked hypertensive-normal clinic BP & 24 h/day/night BP classified as hypertensive; Hyperglycemic-≥ 5.7% HbA1c; Dyslipidemic-> 3.4 mmol/L LDL; Low socio-economic-low SES. *Physical act* physical activity, *GGT* gamma-glutamyl transferase, *BP* blood pressure; *HbA1c* glycated haemoglobin, *HDL* high-density lipoprotein, *LDL* low-density lipoprotein, *ROS* reactive oxygen species, *SES* socio-economic status, *eGFR* estimated glomerular filtration rate, *CVD* cardiovascular disease.

**Fig. 2:**
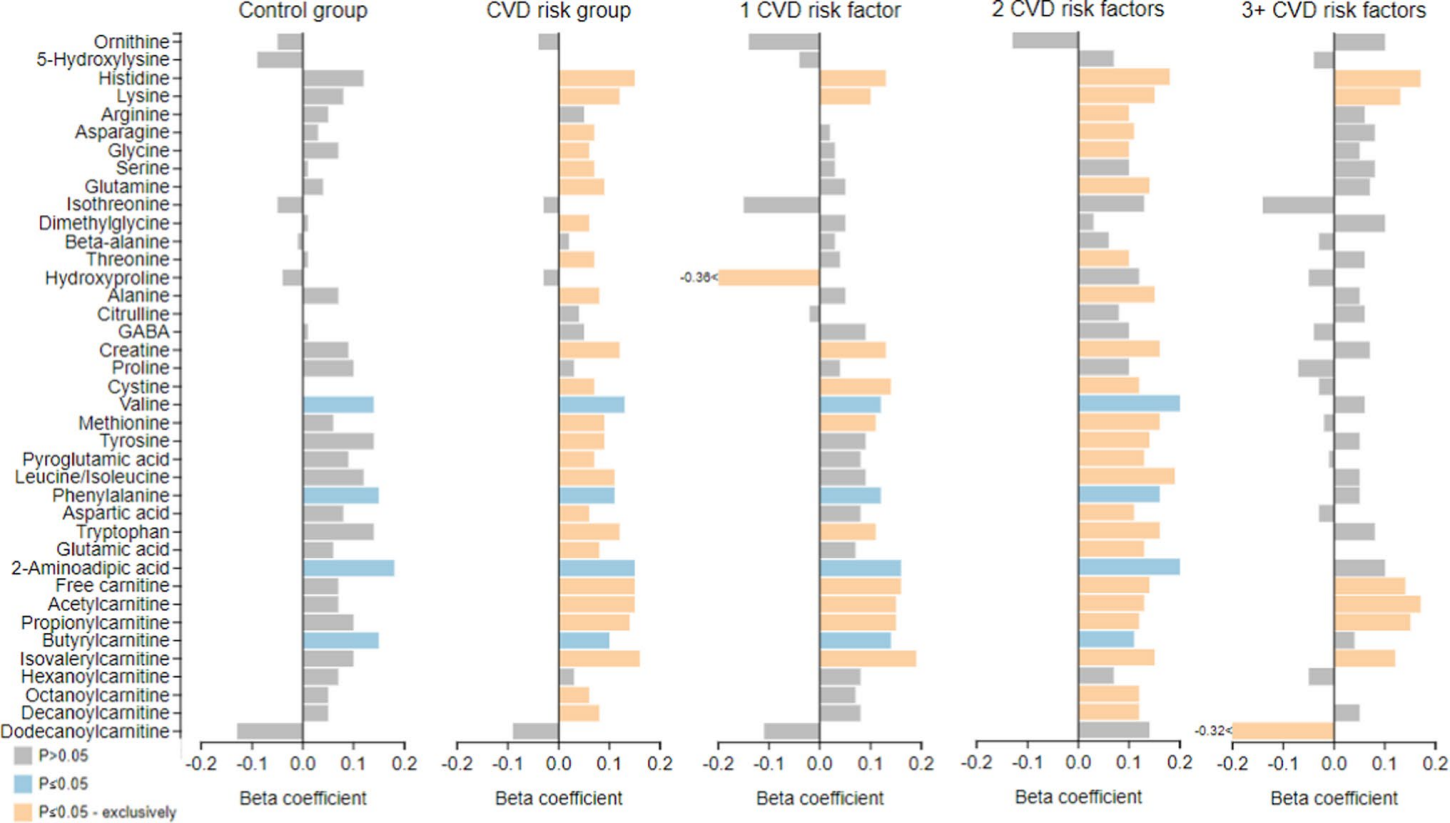
Multi-variable adjusted regression analysis with estimated glomerular filtration rate (cystatin C-based) as the dependent variable, with the metabolomics data in control, cardiovascular disease risk group and cardiovascular disease risk clusters. Test used: Multiple linear regressions. β coefficient are presented-separate models. Estimated glomerular filtration rate (cystatin C-based) adjusted for age, sex, ethnicity, protein intake, waist-to-height ratio, physical activity, cotinine, GGT, 24 h systolic BP, HbA1c, LDL, and SES score. Cardiovascular disease risk group criteria: Obese-≥ 0.55 waist-to-height ratio; Physically inactive-< 600 METs for moderate and/or vigorous intensity physical activity; Smoking-≥ 11 ng/mL cotinine & self-reported smoking; Excessive alcohol intake-≥ 49 U/L GGT & self-reported drinking; Masked hypertensive-normal clinic BP & 24 h/day/night BP classified as hypertensive; Hyperglycemic-≥ 5.7% HbA1c; Dyslipidemic-> 3.4 mmol/L LDL; Low socio-economic-low SES. Metabolite concentration expressed as arbitrary units. *CVD* cardiovascular disease

Utilising multiple regression models (adjusted for age, sex, ethnicity, protein intake, waist-to-height ratio, physical activity, cotinine, GGT, 24-h systolic BP, HbA1c, LDL, and SES score) we investigated the associations between eGFR (cystatin C-based) and the metabolites in the control, CVD risk group and CVD risk clusters (Fig. 2 and Supplementary Table 2A-J). In all groups (except the in the 3 + CVD risk cluster), positive associations of eGFR with valine, phenylalanine, 2-aminoadipic acid and butyrylcarnitine (all P ≤ 0.047) were observed. Within the CVD risk group, positive associations were identified between eGFR and various metabolites including histidine, lysine, asparagine, glycine, serine, glutamine, dimethylglycine, threonine, alanine, creatine, cystine, methionine, tyrosine, pyroglutamic acid, leucine/isoleucine, aspartic acid, tryptophan, glutamic acid, free carnitine, acetylcarnitine, propionylcarnitine, isovalerylcarnitine, octanoylcarnitine and decanoylcarnitine (all P ≤ 0.044). The 1 CVD risk cluster showed positive associations between eGFR with histidine, lysine, creatine, cystine, methionine, tryptophan, free carnitine, acetylcarnitine, propionylcarnitine, isovalerylcarnitine, and a negative association between eGFR with hydroxyproline (all P ≤ 0.025). Compared to the CVD risk group, similar results were observed in the 2 CVD risk cluster, with the additional positive association identified between eGFR with arginine (all P ≤ 0.028), and the loss of associations between eGFR with serine and dimethylglycine. In the 3 + CVD risk cluster, positive associations were identified between eGFR with histidine, lysine, free carnitine, acetylcarnitine, propionylcarnitine, isovalerylcarnitine and a negative association between eGFR with dodecanoylcarnitine (all P ≤ 0.027).

Furthermore, in the CVD risk group and CVD risk clusters, risk factors such as age, male sex, Black ethnicity, cotinine, GGT, and LDL cholesterol were found to be associated with eGFR (Supplementary Table 2A-J) (all P ≤ 0.046).

### Supplementary analysis

Despite the demonstrated superiority of cystatin C-based eGFR over creatinine-based eGFR, the latter is more commonly utilised in clinical practice (Spencer et al. 2023). Consequently, we performed a supplementary analysis following the same procedure as for cystatin C-based eGFR (Supplementary Table 3A-J). A comparison of the fully adjusted model between cystatin C-based eGFR and creatinine-based eGFR revealed some differences. In the control group, histidine, lysine, glycine, proline, and leucine/isoleucine were additionally associated with creatinine-based eGFR, while butyrylcarnitine lost significance. In the CVD risk group, arginine, GABA, proline and hexanoylcarnitine were additionally associated with creatinine-based eGFR, while dimethylglycine and acetylcarnitine lost significance. In the 1 CVD risk cluster isothreonine, GABA, tyrosine, pyroglutamic acid, aspartic acid and glutamic acid were additionally associated with creatinine-based eGFR, while lysine, creatine, methionine, free carnitine, acetylcarnitine, propionylcarnitine, butyrylcarnitine and isovalerylcarnitine lost significance. In the 2 CVD risk cluster, GABA, proline and hexanoylcarnitine were additionally associated with creatinine-based eGFR, while arginine, asparagine, glycine, free carnitine, acetylcarnitine, propionylcarnitine, butyrylcarnitine and isovalerylcarnitine lost significance. Lastly, in the 3 + CVD risk cluster, alanine and decanoylcarnitine were additionally associated with creatinine-based eGFR, while free carnitine, acetylcarnitine, propionylcarnitine, isovalerylcarnitine and dodecanoylcarnitine lost significance.

## Discussion

In this study, we sought to determine the associations between eGFR and urinary metabolites in young adults categorised based on the presence or absence of CVD risk factors. Our findings revealed lower cystatin C-based eGFR, with no difference in creatinine-based eGFR in the CVD risk group, 2 and 3+ CVD risk clusters compared to the control group. This may be linked to creatinine’s limitations in accuracy and sensitivity (Spencer et al. 2023), particularly in the context of the young without kidney disease. The positive associations between eGFR and several metabolites were only present in the CVD risk group and CVD risk clusters, which may demonstrate altered renal reabsorption of these metabolites and/or AAA and BCAA metabolism, energy metabolism and oxidative stress. In contrast to other metabolomic studies, which were mostly conducted in aged adults and in those with established kidney disease (Danilova et al. 2023), our findings highlight the early metabolic changes associated with eGFR in individuals at risk for the early development of CVD. In this regard, Danilova et al. (2023) highlighted consistent findings in metabolomic studies, showing changes in urinary composition related to mitochondrial and membrane dysfunction, oxidative stress, and metabolic abnormalities, including tricarboxylic acid cycle, amino acid, and fatty acid metabolism abnormalities.

### Aromatic amino acid metabolism

**Fig. 3:**
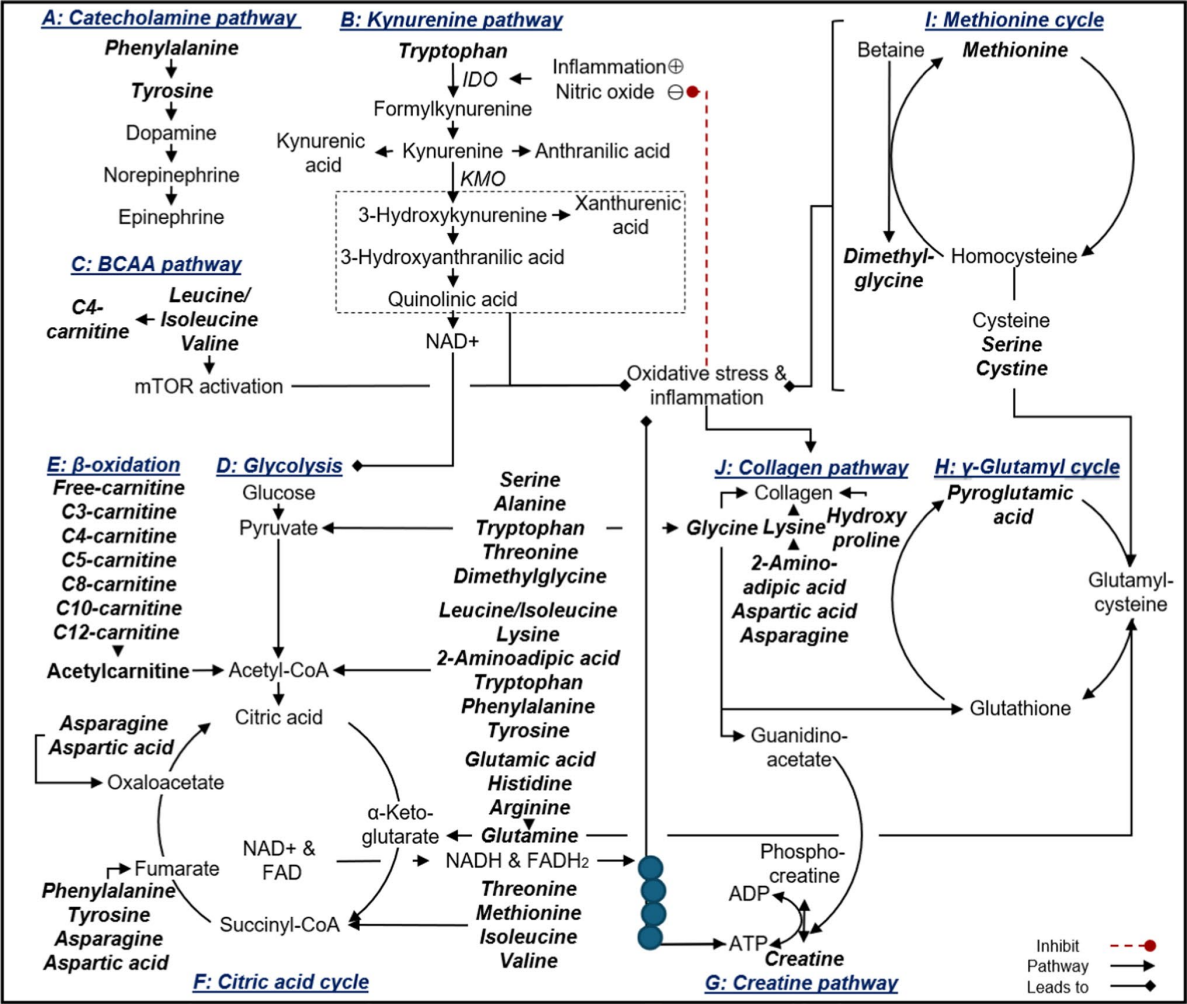
Estimated glomerular filtration rate (cystatin C-based) relates to altered metabolic pathways within the cardiovascular disease risk group and cardiovascular disease risk clusters. Within the CVD risk group and CVD risk clusters, eGFR showed associations with metabolites linked to AAA metabolism (catecholamine and tryptophan pathways), BCAA metabolism, energy metabolism (glycolysis, β-oxidation and creatine pathway) and oxidative stress (y-glutamyl cycle). *IDO* indoleamine-2,3-dioxygenase, *KMO* kynurenine 3-monooxygenase, *NAD+ * nicotinamide adenine dinucleotide; *mTOR* mammalian target of rapamycin, *FAD* flavin adenine dinucleotide, *ADP* adenosine diphosphate, *ATP* adenosine triphosphate

In the CVD risk group, positive associations were found between eGFR and phenylalanine (additionally associated with eGFR in the control group, 1 and 2 CVD risk clusters) and tyrosine (additionally associated with eGFR in the 2 CVD risk cluster). In line with this, another study in apparently healthy adults found similar results for phenylalanine, demonstrating a positive association with the progressive decline in eGFR (Mahbub et al. 2021). Phenylalanine, an essential amino acid, converts to tyrosine, a precursor for catecholamines, including dopamine, norepinephrine, and epinephrine (Fig. 3, A). In this regard, the kidney possesses the complete enzymatic machinery essential for maintaining a local dopaminergic system, where the production of renal dopamine relies on the precursor L-dihydroxyphenylalanine (downstream product of tyrosine via the action of tyrosine hydroxylase) and the activity of dopa decarboxylase (Choi et al. 2015). Dopamine was shown to facilitate natriuresis, diuresis, and enhances renal blood flow via renal vasodilation (at low doses) and enhance cardiac output (at high doses) (Choi et al. 2015; Motiejunaite et al. 2021). Furthermore, dopamine exhibits potent anti-inflammatory, antioxidant and immunomodulatory properties within the kidney. (Choi et al. 2015). On the other hand, the impact of norepinephrine and epinephrine (produced within the sympathetic nervous system and adrenal glands) on the heart and blood vessels elicits positive inotropic, chronotropic and vasoconstrictor effects (Motiejunaite et al. 2021). These actions can indirectly increase renal blood flow by elevating BP (Ivy and Bailey 2014). The observed positive associations between eGFR and these AAAs, especially tyrosine may be linked to the impact of dopamine, norepinephrine and epinephrine on the kidneys, and thus maintaining an appropriate eGFR amid the presence of cardiovascular risk factors.

Furthermore, in the CVD risk group, 1 and 2 CVD risk clusters, eGFR revealed a positive association with tryptophan, an essential amino acid. Dysregulated tryptophan metabolism has been linked to both kidney disease such as acute and chronic kidney disease and CVD (Hui et al. 2023; Song et al. 2017). Tryptophan catabolism occurs predominantly (> 95%) via the kynurenine pathway (Fig. 3, B), where tryptophan undergoes conversion to formylkynurenine (subsequently converted to kynurenine), this metabolic process is governed by two rate-limiting enzymes: tryptophan 2,3-dioxygenase (TDO–basal tryptophan metabolism) in the liver and indoleamine-2,3-dioxygenase (IDO, activated by oxidative and inflammatory conditions) in extrahepatic tissues (Hui et al. 2023; Song et al. 2017). The initiation of tryptophan metabolism under oxidative and inflammatory conditions, coupled with the inflammatory and oxidative nature of some downstream products, establishes a cycle of IDO activation, potentially leading to complications like cardiovascular and kidney dysfunction (Hui et al. 2023; Song et al. 2017). In this young study population without CVD, but with CVD risk factors, ROS levels and the inflammatory marker C-reactive protein were higher (but still within normal ranges). Thus, targeting key enzymes in this pathway, such as kynurenine 3-monooxygenase (KMO), which contributes to the formation of pro-oxidative and pro-inflammatory metabolites, may offer potential benefits. This approach may lead to limiting the harmful effects while preserving the protective aspects of the pathway, including the formation of antioxidant metabolites like kynurenic acid and anthranilic acid (different branches of this pathway) (Lugo-Huitrón et al. 2011; Francisco-Marquez et al. 2016). Furthermore, kynurenine can promote vasodilation (Song et al. 2017; Wang et al. 2010). In our prior investigation on markers of arterial stiffness and urinary metabolomics in young adults with early CVD risk, we uncovered an inverse relationship between pulse wave velocity and tryptophan (du Toit et al. 2023b). We hypothesised that this observed association may signify an activated kynurenine pathway, leading to vasodilation, and thus preserving vascular tone within physiological levels (du Toit et al. 2023b). Aligning with this hypothesis, this vasodilatory response might contribute to an increase in renal blood flow, and consequently eGFR, however, uncontrolled kynurenine pathway activation may contribute to further kidney dysfunction through increased inflammation and oxidative stress.

### Branched chain amino acid metabolism

In the CVD risk group and 2 CVD risk cluster, eGFR showed associations with BCAAs such as leucine/isoleucine and valine (additionally associated with eGFR in the control group and 1 CVD risk cluster). Additionally, there was an association with butyrylcarnitine (additionally associated with eGFR in the control group, 1 and 2 CVD risk clusters), a byproduct of BCAA metabolism. Similar findings were reported in a study involving apparently healthy adults for leucine and isoleucine (Mahbub et al. 2021). Branched-chain amino acids, along with other nutrient signals such as insulin and primer amino acids, and growth factor signalling lead to mammalian target of rapamycin (mTOR) activation (Laplante and Sabatini 2012; Dyachok et al. 2016) (Fig. 3, C). Under normal physiological conditions, mTOR signalling preserves the homeostasis of podocytes and tubular cells (Gui and Dai 2020). However, in cases of kidney injury, mTOR activation in tubular cells and interstitial fibroblasts supports renal regeneration and repair, accompanied by glomerular hypertrophy and interstitial fibrosis under persistent activation (Gui and Dai 2020). Our hypothesis suggests that the association between eGFR and BCAAs may indicate mTOR activation, signifying kidney adaptation to maintain an appropriate eGFR amid increased CVD risk. However, it is crucial to ensure that mTOR activation remains in a compensatory state to prevent deregulated activation and pathological consequences leading to kidney dysfunction (Laplante and Sabatini 2012; Gui and Dai 2020). Persistent mTOR activation may ultimately exacerbate kidney dysfunction, underscoring the importance of maintaining a balanced and regulated mTOR signalling for kidney health (Laplante and Sabatini 2012; Gui and Dai 2020).

### Kidney energy metabolism

The kidneys are among the most metabolically active organs, being rich in mitochondria and displaying high energy metabolism. The majority of renal energy expenditure is dedicated to preserving fluid and electrolyte balance, nutrient reabsorption and elimination of waste products (Liu et al. 2022; Mårtensson 2019). The majority of adenosine triphosphate (ATP) produced in the healthy kidney is through oxidative phosphorylation (glycolysis (Fig. 3, D) and β-oxidation (Fig. 3, E)) using various fuels such as glucose, amino acids and fatty acids (Liu et al. 2022; Mårtensson 2019). In the CVD risk group and CVD risk clusters, eGFR showed positive associations with various amino acids and acetylcarnitines (and a negative association between eGFR with dodecanoylcarnitine in the 3 + CVD risk cluster, which may indicate that short-medium chain fatty acids being utilized for energy with the presence of increasing risk factors). Essentially all of these amino and fatty acids feed into glycolysis or the citric acid cycle on different levels such as pyruvate, acetyl-CoA or various citric acid cycle intermediates (Fig. 3, F) to produce the reducing agents flavin adenine dinucleotide (FADH_2_) and nicotinamide adenine dinucleotide (NADH), which subsequently enters the electron transport chain to generate ATP and ammonia (enters the urea cycle) (Akram 2014). Furthermore, the creatine pathway (Fig. 3, G), together with creatine kinase serves a crucial function in buffering the energy demands of the kidneys by providing near-instantaneous regeneration of ATP during periods of heightened workload (Clarke et al. 2020; Flahault et al. 2016). In the CVD risk group, 2 and 3 + CVD risk clusters, creatine kinase levels were lower compared to the control, which may reflect adversely in the progression of kidney disease (Clarke et al. 2020; Flahault et al. 2016). Adequate oxygen delivery to the kidneys is therefore vital for maintaining normal energy metabolism (Liu et al. 2022; Mårtensson 2019). While increased renal blood flow positively impacts renal oxygen delivery, the situation in the kidneys differs from other major organs due to concurrent changes in glomerular filtration rate and filtered solute load (Liu et al. 2022; Mårtensson 2019). These factors, in turn, increase renal oxygen consumption due to increased tubular reabsorption (Liu et al. 2022; Mårtensson 2019). Recent findings indicate that disturbances in renal bioenergetics constitute a significant pathophysiologic occurrence in the progression of kidney disease (Liu et al. 2022). In the progression to kidney disease there is a loss of functional nephrons which subsequently stimulates compensatory hyperfiltration and hypertrophy in intact nephrons which increases workload, thus the energy demand (Schnaper 2017). Our findings may therefore highlight the early stages of this compensatory mechanism as a lower eGFR was demonstrated in the CVD risk group, 2 and 3 + CVD risk clusters compared to the control group (but still within normal ranges). Furthermore, upregulated energetics may promote inflammation and renal fibrosis, through the autocrine and paracrine actions of adenosine and ATP signalling pathways in the kidneys (Liu et al. 2022). It is therefore essential to maintain appropriate kidney energetics.

### Oxidative stress

In addition to the previously mentioned proinflammatory and oxidative pathways, several metabolites were associated with eGFR in the CVD risk group and CVD risk clusters that may act as precursors for the γ-glutamyl cycle (Fig. 3, H), a crucial antioxidant pathway. These metabolites include histidine, glycine, serine, glutamine, dimethylglycine, threonine, alanine, cystine, methionine, pyroglutamic acid, tryptophan and glutamic acid. The γ-glutamyl cycle plays a role in producing glutathione, where various amino acids serve as precursors for its synthesis (Lushchak 2012). Glutathione, a vital antioxidant, helps maintain a healthy redox state (Lushchak 2012). The positive associations found between eGFR and the precursors for glutathione suggest increased availability of amino acids for glutathione production, potentially in response to heightened oxidative stress, as indicated by elevated ROS levels in the CVD risk group and CVD risk clusters compared to the control group. Furthermore, some precursors of the γ-glutamyl cycle, such as methionine and cysteine (Fig. 3, I), may contribute to an oxidative environment, exerting pro-inflammatory, pro-oxidant, and pro-atherogenic effects (Rehman et al. 2020). This oxidative environment could impact nitric oxide (NO) bioavailability and vascular damage (fibrosis) (Rehman et al. 2020; Cyr et al. 2020) (Fig. 3, J). In support of this, positive associations were observed between eGFR and lysine (with asparagine, aspartic acid, and 2-aminoadipic acid (additionally associated with eGFR in the control group) as precursors) and glycine in the CVD risk group and the CVD risk clusters (particularly the 2 CVD risk cluster), and a negative association between eGFR with hydroxyproline in the 1 CVD risk cluster, highlighting their potential involvement in fibrosis through collagen production.

Taken together, kidney dysfunction progression involves damage to the glomerulus and renal tubules due to among others, inflammation, resulting in the loss of functional nephrons (Schnaper 2017). This is followed by compensatory hyperfiltration and nephron hypertrophy, initially aiding but eventually leading to metabolic imbalances and a self-sustaining cycle of inflammation and fibrosis (Schnaper 2017). In this young study population with CVD risk factors, the associations found between eGFR, and the various metabolites may suggest alterations in renal reabsorption of functional metabolites associated with AAA and BCAA metabolism, energy metabolism and oxidative stress, which may be implicated in early kidney dysfunction. Therefore, early correction of these metabolic changes and/or targeting identified pathways may offer strategies to prevent alterations in kidney function.

## Strengths and limitations

Our study's primary strength lies in its emphasis on high-level metabolomics data derived from a young, apparently healthy population of African and European descent without CVD, thereby minimising the impact of age and existing diseases on metabolism. Additionally, our exploration of these findings in a multi-ethnic cohort, a relatively limited approach in Africa, contributes to the study's uniqueness. However, it is crucial to acknowledge the hypothesis-generating nature of this study, and future research is needed to confirm and validate the observed findings. Furthermore, it should be noted that for the metabolomics data, urinary creatinine was used to obtain a predetermined urine volume, this therefore strengthened our focus on cystatin C (serum)-based eGFR (superior compared to creatinine (serum)-based eGFR (Spencer et al. 2023)).The cross-sectional design also limits the ability to infer causal relationships, and while efforts were made to minimise confounding, the influence of residual confounding cannot be entirely ruled out.

## Conclusion

In conclusion, in a young study population without CVD, but with CVD risk factors, eGFR were positively associated with metabolites linked to AAA and BCAA metabolism, energy metabolism and oxidative stress. These associations may indicate altered reabsorption of these metabolites or altered metabolic regulation as a protective response in the early stages of kidney dysfunction to maintain an appropriate eGFR among increased CVD risk.

## Supplementary Information

Below is the link to the electronic supplementary material.

Supplementary file1 (DOCX 494 KB)

## Data Availability

Data used in this study is available upon reasonable request to the principal investigator of the study.
