# Supplementary material for: Exploring the interplay between kidney function and urinary metabolites in young adults: the African-PREDICT study
Source: Amino Acids. 2024 Aug 29;56:53. doi: 10.1007/s00726-024-03412-7 (PMC11362211; doi:10.1007/s00726-024-03412-7)
Supplement: Supplementary file 1 [file 726_2024_3412_MOESM1_ESM.docx]

**Exploring the interplay between kidney function and urinary metabolites in young adults: the African-PREDICT study**

Wessel L. du Toit^1,2^, Ruan Kruger^1,3^, Lebo F. Gafane-Matemane^1,3^_,_ Aletta E. Schutte^1,3,4^, Roan Louw^5^, Catharina M.C. Mels^1,3^

^1^*Hypertension in Africa Research Team (HART), North-West University, Potchefstroom, South Africa.*

^2^*Cardiovascular Pathophysiology and Genomics Research Unit (CPGRU), University of the Witwatersrand, Johannesburg, South Africa.*

^3^*MRC Research Unit for Hypertension and Cardiovascular Disease, North-West University, Potchefstroom, South* *Africa.*

*^4^School of Population Health, University of New South Wales; The George Institute for Global Health, Sydney, Australia.*

*^5^Human Metabolomics, North-West University, Potchefstroom Campus, Potchefstroom, South Africa.*

Wessel L. du Toit - ORCID:0000-0002-1883-8456

Ruan Kruger - ORCID:0000-0001-7680-2032

Lebo F. Gafane-Matemane - ORCID:0000-0003-4596-7218

Aletta E. Schutte - ORCID:0000-0001-9217-4937

Roan Louw - ORCID:0000-0002-6542-8644

Catharina M.C. Mels - ORCID:0000-0003-0138-3341

**Correspondence:**

Prof. Carina Mels

Hypertension in Africa Research Team (HART)

North-West University

Private Bag X6001

Potchefstroom

2520

South Africa

Tel: +27 18 299 1983

Fax: +27 18 285 2432

E-mail: [carina.mels@nwu.ac.za](mailto:carina.mels@nwu.ac.za)

**Supplementary Table 1. Metabolomic data of control, cardiovascular disease risk group and cardiovascular disease risk clusters**

| ***Metabolomic data*** | **Control group (N=166)** | **CVD risk group (N=1036)** | **1 CVD risk factor (N=344)** | **2 CVD risk factors (N=360)** | **3+ CVD risk factors (N=332)** |
| --- | --- | --- | --- | --- | --- |
| Ornithine, AU | 8.93 (7.24; 11.0) | 7.46 (6.92; 7.94) | 7.55 (2.14; 24.3) | 7.69 (2.02; 25.2) | 7.31 (2.26; 22.0) |
| 5-Hydroxylysine, AU | 9.23 (7.94; 10.7) | 9.71 (9.12; 10.2) | 9.57 (1.95; 23.7) | 9.43 (2.07; 25.0) | 10.1 (1.75; 27.0) |
| Histidine, AU | 731 (661; 813) | 731 (708; 759) | 697 (207; 1762) | **774 (249; 2062)*** | 755 (249; 2183) |
| Lysine, AU | 36.1 (31.6; 41.7) | 37.1 (35.5; 38.9) | 36.8 (12.1; 140) | 36.8 (12.4; 153) | 38.1 (13.3; 165) |
| Arginine, AU | 11.8 (10.7; 12.9) | 12.1 (11.8; 12.6) | 12.1 (5.59; 29.6) | 12.0 (6.03; 26.4) | 12.1 (6.15; 28.7) |
| Asparagine, AU | 24.6 (22.4; 27.5) | 24.1 (22.9; 25.1) | 23.8 (9.67; 59.1) | 24.5 (9.53; 60.6) | 24.2 (9.11; 68.8) |
| Glycine, AU | 504 (437; 575) | 526 (501; 550) | 512 (147; 1937) | 536 (134; 1959) | 540 (134; 1882) |
| Serine, AU | 227 (204; 251) | 236 (224; 245) | 232 (79.5; 600) | 236 (79.0; 618) | **245 (84.7; 644)*** |
| Glutamine, AU | 223 (195; 251) | 235 (224; 245) | 231 (69.1; 755) | 241 (65.2; 758) | 240 (81.1; 762) |
| Isothreonine, AU | 3.50 (2.51; 4.90) | 3.72 (3.31; 4.17) | 4.51 (1.86; 19.9) | 3.48 (1.63; 9.02) | 3.35 (1.45; 16.7) |
| Dimethylglycine, AU | 16.2 (12.9; 20.4) | 15.9 (14.5; 17.4) | 14.1 (3.26; 174) | 17.1 (3.14; 294) | 17.4 (3.30; 257) |
| Beta-alanine, AU | 46.0 (36.3; 58.9) | 51.6 (46.8; 56.2) | 54.8 (4.45; 442) | 49.6 (4.81; 322) | 52.4 (4.29; 358) |
| Threonine, AU | 78.3 (69.2; 89.1) | 81.5 (77.6; 85.1) | 81.7 (25.9; 269) | 80.8 (25.7; 253) | 83.3 (28.8; 249) |
| Hydroxyproline, AU | 3.36 (2.57; 4.37) | 3.51 (3.24; 3.80) | 3.56 (1.67; 14.6) | 3.66 (1.43; 13.4) | 3.33 (1.40; 10.8) |
| Alanine, AU | 226 (200; 257) | 249 (234; 263) | 237 (69.2; 831) | **250 (76.6; 837)*** | **268 (74.7; 809)*** |
| Citrulline, AU | 4.66 (4.07; 5.25) | 4.72 (4.47; 4.90) | 4.71 (2.10; 14.6) | 4.75 (2.02; 13.8) | 4.73 (2.18; 15.1) |
| GABA, AU | 0.25 (0.22; 0.27) | 0.26 (0.25; 0.27) | 0.26 (0.13; 0.82) | 0.25 (0.13; 0.67) | 0.25 (0.13; 0.75) |
| Creatine, AU | 16.6 (14.5; 19.1) | **19.3 (18.2; 20.4)*** | 18.6 (7.35; 102) | 18.7 (8.09; 111) | **20.9 (7.62; 137)*** |
| Proline, AU | 1.73 (1.58; 1.91) | 1.83 (1.78; 1.91) | 1.83 (0.81; 5.02) | 1.76 (0.70; 4.59) | 1.86 (0.77; 4.35) |
| Cystine, AU | 0.61 (0.54; 0.68) | 0.67 (0.65; 0.69) | 0.63 (0.27; 1.53) | 0.67 (0.25; 2.00) | **0.72 (0.27; 2.91)*** |
| Valine, AU | 4.73 (4.27; 5.25) | 5.20 (5.01; 5.37) | 5.14 (1.83; 13.4) | 5.06 (1.90; 12.5) | 5.37 (2.11; 13.5) |
| Methionine, AU | 1.14 (1.02; 1.26) | 1.20 (1.15; 1.26) | 1.17 (0.45; 3.01) | 1.21 (0.42; 3.36) | **1.24 (0.51; 3.31)*** |
| Tyrosine, AU | 32.4 (28.8; 36.3) | **37.8 (36.3; 39.8)*** | 36.4 (13.0; 99.8) | 36.4 (12.7; 91.0) | **41.2 (16.3; 110)**** |
| Pyroglutamic acid, AU | 23.3 (20.9; 25.7) | 24.8 (24.0; 25.7) | 24.6 (9.67; 66.6) | 24.6 (8.95; 60.0) | 25.5 (10.1; 71.5) |
| Leucine/Isoleucine, AU | 12.0 (10.7; 13.5) | 13.2 (12.6; 13.8) | 12.7 (4.47; 32.0) | 13.0 (5.16; 35.5) | **13.9 (5.74; 37.2)*** |
| Phenylalanine, AU | 16.3 (14.5; 18.2) | 18.4 (17.8; 19.1) | 17.6 (5.61; 45.1) | 18.2 (6.60; 50.8) | **19.6 (7.90; 55.0)*** |
| Aspartic acid, AU | 4.58 (4.17; 5.01) | 4.78 (4.57; 4.90) | 4.68 (2.09; 10.8) | 4.83 (2.15; 12.6) | 4.82 (2.20; 12.2) |
| Tryptophan, AU | 1.13 (0.98; 1.29) | 1.30 (1.23; 1.38) | 1.28 (0.35; 4.83) | 1.24 (0.29; 4.23) | 1.37 (0.43; 5.49) |
| Glutamic acid, AU | 2.17 (1.95; 2.45) | 2.37 (2.29; 2.45) | 2.36 (0.86; 6.85) | 2.37 (0.79; 6.46) | 2.40 (0.85; 6.86) |
| 2-Aminoadipic acid, AU | 0.87 (0.76; 1.00) | 0.89 (0.83; 0.93) | 0.89 (0.23; 2.90) | 0.87 (0.24; 2.96) | 0.89 (0.23; 2.83) |
| Free carnitine, AU | 45.3 (40.7; 50.1) | 46.8 (44.7; 49.0) | 47.5 (16.4; 136) | 47.5 (17.4; 129) | 44.5 (16.2; 132) |
| Acetylcarnitine, AU | 11.9 (9.55; 14.8) | 11.4 (10.5; 12.3) | 12.4 (0.74; 80.6) | 11.3 (0.95; 80.0) | **9.90 (0.62; 63.0)*** |
| Propionylcarnitine, AU | 1.65 (1.35; 2.00) | 1.70 (1.58; 1.82) | 1.76 (0.23; 10.4) | 1.74 (0.23; 9.08) | 1.57 (0.23; 7.94) |
| Butyrylcarnitine, AU | 11.5 (10.0; 13.2) | 11.2 (10.7; 11.8) | 12.0 (3.05; 41.7) | 11.0 (3.02; 38.3) | 10.5 (3.06; 41.3) |
| Isovalerylcarnitine, AU | 0.51 (0.45; 0.59) | 0.58 (0.55; 0.60) | 0.59 (0.12; 2.40) | 0.55 (0.11; 2.27) | 0.58 (0.14; 2.24) |
| Hexanoylcarnitine, AU | 0.06 (0.05; 0.07) | 0.07 (0.06; 0.07) | 0.06 (0.02; 0.21) | 0.06 (0.02; 0.21) | **0.07 (0.03; 0.22)*** |
| Octanoylcarnitine, AU | 0.31 (0.28; 0.34) | 0.34 (0.32; 0.35) | 0.34 (0.16; 0.90) | 0.32 (0.15; 0.87) | 0.34 (0.16; 0.97) |
| Decanoylcarnitine, AU | 0.18 (0.16; 0.21) | 0.21 (0.19; 0.22) | **0.22 (0.08; 0.76)*** | 0.19 (0.05; 0.62) | 0.21 (0.05; 0.77) |
| Dodecanoylcarnitine, AU | 0.02 (0.02; 0.03) | 0.02 (0.02; 0.03) | 0.02 (0.01; 0.08) | 0.02 (0.01; 0.07) | 0.02 (0.01; 0.08) |

Test used: ANCOVAs (adjusted for sex, ethnicity, and protein intake). Data are presented as geometric mean with 95% confidence intervals. Bold values denote P≤0.05; *P≤0.05; **P≤0.001. Cardiovascular disease risk group criteria: Obese - ≥0.55 waist-to-height ratio; Physically inactive - <600 METs for moderate and/or vigorous intensity physical activity; Smoking - ≥11 ng/mL cotinine & self-reported smoking; Excessive alcohol intake - ≥49 U/L GGT & self-reported drinking; Masked hypertensive - normal clinic BP & 24h/day/night BP classified as hypertensive; Hyperglycemic - ≥5.7% HbA1c; Dyslipidemic - >3.4 mmol/L LDL; Low socio-economic - low SES.

*Abbreviations*: AU, arbitrary units; CVD, cardiovascular disease.

.

**Supplementary Table 2A. Multi-variable adjusted regression analysis with estimated glomerular filtration rate (cystatin C-based) as the dependent variable, with the metabolomics data in control, cardiovascular disease risk group and cardiovascular disease risk clusters**

|  | **eGFR (cystatin-C), ml/min/1.73m^2^** | | | | | | | | | |
| --- | --- | --- | --- | --- | --- | --- | --- | --- | --- | --- |
|  | **Control group**  **(N=166)** | | **CVD risk group**  **(N=1036)** | | **1 CVD risk factor**  **(N=344)** | | **2 CVD risk factors (N=360)** | | **3+ CVD risk factors (N=332)** | |
| ***Metabolomic data*** | **Adj R^2^** | **β (95%Cl)** | **Adj R^2^** | **β (95%Cl)** | **Adj R^2^** | **β (95%Cl)** | **Adj R^2^** | **β (95%Cl)** | **Adj R^2^** | **β (95%Cl)** |
| Ornithine, AU | **0.28*** | -0.05 (-0.37; 0.26) | **0.30**** | -0.04 (-0.13; 0.04) | **0.27**** | -0.14 (-0.34; 0.04) | **0.30**** | -0.13 (-0.25; 0.02) | **0.20**** | 0.10 (-0.06; 0.25) |
| Age, years |  | -0.16 (-0.57; 0.17) |  | -0.08 (-0.17; 0.02) |  | -0.10 (-0.29; 0.09) |  | -0.09 (-0.25; 0.08) |  | -0.03 (-0.18; 0.13) |
| Sex, female/male |  | **0.34 (<0.01; 0.80)*** |  | **0.26 (0.15; 0.36)**** |  | **0.34 (0.12; 0.57)*** |  | **0.23 (0.05; 0.39)*** |  | **0.21 (0.02; 0.38)*** |
| Ethnicity, Black/White |  | -0.17 (-0.65; 0.19) |  | **-0.18 (-0.29; -0.07)**** |  | -0.17 (-0.40; 0.05) |  | **-0.23 (-0.40; -0.04)*** |  | -0.16 (-0.36; 0.05) |
| Protein intake, g |  | -0.02 (-0.34; 0.31) |  | 0.04 (-0.05; 0.12) |  | 0.01 (-0.18; 0.20) |  | 0.07 (-0.08; 0.22) |  | -0.02 (-0.16; 0.13) |
| Waist-to-height ratio |  | 0.05 (-0.35; 0.50) |  | 0.04 (-0.05; 0.14) |  | 0.10 (-0.11; 0.42) |  | 0.02 (-0.17; 0.22) |  | -0.04 (-0.17; 0.11) |
| Physical act, kCal/kg/day |  | 0.03 (-0.39; 0.50) |  | 0.03 (-0.05; 0.11) |  | 0.01 (-0.20; 0.22) |  | 0.05 (-0.09; 0.18) |  | 0.01 (-0.12; 0.13) |
| Cotinine, ng/ml |  |  |  | -0.08 (-0.16; 0.01) |  | -0.07 (-0.37; 0.15) |  | -0.03 (-0.17; 0.12) |  | -0.14 (-0.25; 0.02) |
| GGT, U/L |  | **-0.36 (-0.93; -0.06)*** |  | **-0.26 (-0.36; -0.16)**** |  | **-0.32 (-0.58; -0.13)*** |  | **-0.22 (-0.42; -0.07)*** |  | **-0.22 (-0.35; -0.04)*** |
| 24h Systolic BP, mmHg |  | -0.09 (-0.63; 0.36) |  | -0.01 (-0.11; 0.09) |  | <0.01 (-0.23; 0.23) |  | -0.01 (-0.18; 0.17) |  | -0.04 (-0.20; 0.14) |
| HbA1c, (%) |  | <0.01 (-0.40; 0.40) |  | -0.04 (-0.13; 0.05) |  | -0.03 (-0.24; 0.17) |  | -0.05 (-0.19; 0.10) |  | -0.06 (-0.20; 0.10) |
| LDL cholesterol, mmol/L |  | **-0.35 (-1.10; -0.10)*** |  | **-0.32 (-0.39; -0.21)**** |  | **-0.29 (-0.56; -0.13)**** |  | **-0.36 (-0.51; -0.19)**** |  | **-0.31 (-0.38; -0.10)**** |
| SES score |  | 0.05 (-0.50; 0.70) |  | <0.01 (-0.10; 0.10) |  | -0.05 (-0.30; 0.19) |  | 0.05 (-0.14; 0.24) |  | 0.01 (-0.17; 0.18) |
| 5-Hydroxylysine, AU | **0.40**** | -0.09 (-0.28; 0.08) | **0.31**** | <0.01 (-0.06; 0.06) | **0.31**** | -0.04 (-0.16; 0.07) | **0.32**** | 0.07 (-0.03; 0.18) | **0.22**** | -0.04 (-0.14; 0.07) |
| Age, years |  | -0.14 (-0.39; 0.05) |  | **-0.08 (-0.15; -0.01)*** |  | -0.09 (-0.21; 0.04) |  | -0.12 (-0.23; 0.01) |  | -0.02 (-0.14; 0.11) |
| Sex, female/male |  | **0.35 (0.19; 0.65)**** |  | **0.25 (0.17; 0.32)**** |  | **0.27 (0.14; 0.41)**** |  | **0.21 (0.08; 0.33)**** |  | **0.23 (0.07; 0.35)*** |
| Ethnicity, Black/White |  | -0.18 (-0.48; 0.01) |  | **-0.18 (-0.26; -0.10)**** |  | **-0.15 (-0.3; <0.01)*** |  | **-0.23 (-0.36; -0.08)**** |  | -0.16 (-0.31; 0.01) |
| Protein intake, g |  | -0.01 (-0.20; 0.18) |  | 0.04 (-0.03; 0.10) |  | 0.02 (-0.10; 0.14) |  | 0.09 (-0.02; 0.20) |  | -0.02 (-0.13; 0.10) |
| Waist-to-height ratio |  | 0.03 (-0.19; 0.30) |  | 0.04 (-0.03; 0.11) |  | 0.10 (-0.02; 0.33) |  | 0.03 (-0.11; 0.18) |  | -0.05 (-0.15; 0.07) |
| Physical act, kCal/kg/day |  | 0.03 (-0.21; 0.30) |  | 0.03 (-0.03; 0.09) |  | 0.03 (-0.10; 0.17) |  | 0.06 (-0.05; 0.15) |  | 0.01 (-0.09; 0.11) |
| Cotinine, ng/ml |  |  |  | **-0.08 (-0.13; -0.01)*** |  | -0.04 (-0.22; 0.11) |  | -0.03 (-0.13; 0.08) |  | **-0.16 (-0.23; -0.02)*** |
| GGT, U/L |  | **-0.36 (-0.75; -0.24)**** |  | **-0.25 (-0.33; -0.18)**** |  | **-0.28 (-0.46; -0.17)**** |  | **-0.20 (-0.35; -0.08)**** |  | **-0.23 (-0.33; -0.09)**** |
| 24h Systolic BP, mmHg |  | -0.10 (-0.43; 0.14) |  | -0.01 (-0.09; 0.06) |  | <0.01 (-0.15; 0.15) |  | -0.02 (-0.15; 0.12) |  | -0.02 (-0.15; 0.12) |
| HbA1c, (%) |  | 0.01 (-0.22; 0.25) |  | -0.04 (-0.10; 0.03) |  | -0.02 (-0.16; 0.11) |  | -0.04 (-0.15; 0.07) |  | -0.05 (-0.16; 0.07) |
| LDL cholesterol, mmol/L |  | **-0.35 (-0.88; -0.30)**** |  | **-0.32 (-0.37; -0.23)**** |  | **-0.30 (-0.50; -0.22)**** |  | **-0.34 (-0.46; -0.21)**** |  | **-0.31 (-0.36; -0.13)**** |
| SES score |  | 0.05 (-0.26; 0.44) |  | -0.01 (-0.08; 0.07) |  | -0.07 (-0.24; 0.08) |  | 0.05 (-0.09; 0.19) |  | 0.01 (-0.13; 0.15) |
| Histidine, AU | **0.42**** | 0.12 (-0.02; 0.30) | **0.33**** | **0.15 (0.10; 0.21)**** | **0.33**** | **0.13 (0.03; 0.24)*** | **0.35**** | **0.18 (0.08; 0.26)**** | **0.26**** | **0.17 (0.06; 0.27)*** |
| Age, years |  | -0.15 (-0.37; 0.01) |  | **-0.08 (-0.14; -0.02)*** |  | -0.08 (-0.19; 0.03) |  | **-0.12 (-0.22; -0.01)*** |  | -0.04 (-0.14; 0.07) |
| Sex, female/male |  | **0.32 (0.17; 0.59)**** |  | **0.22 (0.15; 0.28)**** |  | **0.24 (0.13; 0.38)**** |  | **0.19 (0.07; 0.29)**** |  | **0.20 (0.06; 0.31)*** |
| Ethnicity, Black/White |  | **-0.18 (-0.46; -0.03)*** |  | **-0.16 (-0.23; -0.09)**** |  | **-0.13 (-0.27; <0.01)*** |  | **-0.22 (-0.33; -0.09)**** |  | -0.13 (-0.26; 0.02) |
| Protein intake, g |  | -0.01 (-0.18; 0.15) |  | 0.04 (-0.01; 0.10) |  | 0.03 (-0.08; 0.14) |  | 0.09 (-0.01; 0.18) |  | <0.01 (-0.10; 0.10) |
| Waist-to-height ratio |  | 0.03 (-0.17; 0.26) |  | 0.04 (-0.03; 0.10) |  | 0.08 (-0.03; 0.28) |  | 0.03 (-0.09; 0.17) |  | -0.05 (-0.13; 0.06) |
| Physical act, kCal/kg/day |  | 0.03 (-0.18; 0.28) |  | 0.04 (-0.02; 0.09) |  | 0.01 (-0.11; 0.14) |  | 0.06 (-0.03; 0.14) |  | 0.02 (-0.07; 0.11) |
| Cotinine, ng/ml |  |  |  | **-0.07 (-0.11; -0.01)*** |  | -0.02 (-0.17; 0.12) |  | -0.02 (-0.11; 0.08) |  | **-0.14 (-0.20; -0.01)*** |
| GGT, U/L |  | **-0.35 (-0.71; -0.25)**** |  | **-0.24 (-0.30; -0.17)**** |  | **-0.27 (-0.43; -0.17)**** |  | **-0.19 (-0.32; -0.09)**** |  | **-0.21 (-0.30; -0.09)**** |
| 24h Systolic BP, mmHg |  | -0.09 (-0.39; 0.12) |  | -0.02 (-0.09; 0.04) |  | 0.02 (-0.11; 0.16) |  | -0.05 (-0.16; 0.07) |  | -0.02 (-0.14; 0.09) |
| HbA1c, (%) |  | 0.02 (-0.18; 0.23) |  | -0.04 (-0.10; 0.02) |  | -0.03 (-0.15; 0.09) |  | -0.05 (-0.15; 0.04) |  | -0.04 (-0.13; 0.07) |
| LDL cholesterol, mmol/L |  | **-0.33 (-0.82; -0.31)**** |  | **-0.30 (-0.34; -0.22)**** |  | **-0.26 (-0.44; -0.18)**** |  | **-0.34 (-0.44; -0.23)**** |  | **-0.28 (-0.32; -0.12)**** |
| SES score |  | 0.05 (-0.20; 0.42) |  | <0.01 (-0.07; 0.07) |  | -0.09 (-0.25; 0.04) |  | 0.08 (-0.05; 0.20) |  | -0.01 (-0.13; 0.12) |
| Lysine, AU | **0.41**** | 0.08 (-0.07; 0.26) | **0.33**** | **0.12 (0.06; 0.17)**** | **0.32**** | **0.10 (<0.01; 0.21)*** | **0.34**** | **0.15 (0.05; 0.23)*** | **0.25**** | **0.13 (0.02; 0.21)*** |
| Age, years |  | **-0.16 (-0.39; <0.01)*** |  | **-0.09 (-0.15; -0.02)*** |  | -0.08 (-0.20; 0.03) |  | **-0.12 (-0.22; -0.01)*** |  | -0.03 (-0.14; 0.08) |
| Sex, female/male |  | **0.35 (0.21; 0.62)**** |  | **0.25 (0.18; 0.31)**** |  | **0.27 (0.15; 0.40)**** |  | **0.22 (0.10; 0.32)**** |  | **0.24 (0.10; 0.35)**** |
| Ethnicity, Black/White |  | **-0.18 (-0.46; -0.03)*** |  | **-0.19 (-0.26; -0.11)**** |  | -0.16 (-0.29; -0.03)* |  | **-0.25 (-0.36; -0.12)**** |  | **-0.15 (-0.29; -0.01)*** |
| Protein intake, g |  | -0.03 (-0.21; 0.14) |  | 0.04 (-0.02; 0.10) |  | 0.03 (-0.08; 0.14) |  | 0.09 (-0.01; 0.18) |  | <0.01 (-0.10; 0.10) |
| Waist-to-height ratio |  | 0.04 (-0.15; 0.28) |  | 0.03 (-0.03; 0.09) |  | 0.09 (-0.03; 0.29) |  | 0.02 (-0.10; 0.15) |  | -0.05 (-0.14; 0.06) |
| Physical act, kCal/kg/day |  | 0.02 (-0.21; 0.26) |  | 0.04 (-0.02; 0.09) |  | 0.02 (-0.10; 0.14) |  | 0.06 (-0.03; 0.14) |  | 0.02 (-0.07; 0.10) |
| Cotinine, ng/ml |  |  |  | **-0.07 (-0.12; -0.01)*** |  | -0.02 (-0.18; 0.12) |  | -0.03 (-0.12; 0.07) |  | **-0.15 (-0.21; -0.03)*** |
| GGT, U/L |  | **-0.35 (-0.71; -0.25)**** |  | **-0.24 (-0.31; -0.18)**** |  | **-0.27 (-0.44; -0.18)**** |  | **-0.19 (-0.33; -0.10)**** |  | **-0.22 (-0.30; -0.09)**** |
| 24h Systolic BP, mmHg |  | -0.09 (-0.39; 0.12) |  | -0.02 (-0.09; 0.05) |  | 0.02 (-0.12; 0.16) |  | -0.04 (-0.16; 0.08) |  | -0.03 (-0.14; 0.09) |
| HbA1c, (%) |  | <0.01 (-0.20; 0.21) |  | -0.04 (-0.10; 0.02) |  | -0.02 (-0.15; 0.10) |  | -0.05 (-0.14; 0.05) |  | -0.04 (-0.14; 0.07) |
| LDL cholesterol, mmol/L |  | **-0.33 (-0.83; -0.30)**** |  | **-0.31 (-0.35; -0.23)**** |  | **-0.28 (-0.46; -0.20)**** |  | **-0.34 (-0.44; -0.23)**** |  | **-0.30 (-0.33; -0.13)**** |
| SES score |  | 0.05 (-0.21; 0.42) |  | <0.01 (-0.07; 0.07) |  | -0.08 (-0.25; 0.05) |  | 0.08 (-0.05; 0.20) |  | 0.01 (-0.12; 0.13) |

Test used: Multiple linear regressions. Data are presented as adjusted R^2^ with β coefficient and 95% confidence intervals. Estimated glomerular filtration rate (cystatin C-based), adjusted for age, sex, ethnicity, protein intake, waist-to-hight ratio, physical activity, cotinine, GGT, 24h systolic BP, HbA1c, LDL, SES score. Bold values denote P≤0.05; *P≤0.05; **P≤0.001. Cardiovascular disease risk group criteria: Obese - ≥0.55 waist-to-height ratio; Physically inactive - <600 METs for moderate and/or vigorous intensity physical activity; Smoking - ≥11 ng/mL cotinine & self-reported smoking; Excessive alcohol intake - ≥49 U/L GGT & self-reported drinking; Masked hypertensive – normal clinic BP & 24h/day/night BP classified as hypertensive; Hyperglycemic - ≥5.7% HbA1c; Dyslipidemic - >3.4 mmol/L LDL; Low socio-economic – low SES.

*Abbreviations*: AU, arbitrary units; physical act, physical activity; GGT, gamma-glutamyl transferase; BP, blood pressure; HbA1c, glycated haemoglobin; LDL, low density lipoprotein; SES, socio-economic status; GFR, estimated glomerular filtration rate; CVD, cardiovascular disease.

|  | **eGFR (cystatin-C), ml/min/1.73m^2^** | | | | | | | | | |
| --- | --- | --- | --- | --- | --- | --- | --- | --- | --- | --- |
|  | **Control group**  **(N=166)** | | **CVD risk group**  **(N=1036)** | | **1 CVD risk factor**  **(N=344)** | | **2 CVD risk factors (N=360)** | | **3+ CVD risk factors (N=332)** | |
| ***Metabolomic data*** | **Adj R^2^** | **β (95%Cl)** | **Adj R^2^** | **β (95%Cl)** | **Adj R^2^** | **β (95%Cl)** | **Adj R^2^** | **β (95%Cl)** | **Adj R^2^** | **β (95%Cl)** |
| Arginine, AU | **0.41**** | 0.05 (-0.12; 0.24) | **0.31**** | 0.05 (<0.01; 0.10) | **0.31**** | <0.01 (-0.10; 0.10) | **0.33**** | **0.10 (<0.01; 0.20)*** | **0.24**** | 0.06 (-0.04; 0.15) |
| Age, years |  | -0.16 (-0.38; 0.01) |  | **-0.09 (-0.15; -0.02)*** |  | -0.09 (-0.21; 0.02) |  | **-0.12 (-0.22; -0.01)*** |  | -0.03 (-0.14; 0.08) |
| Sex, female/male |  | **0.35 (0.21; 0.63)**** |  | **0.26 (0.18; 0.32)**** |  | **0.27 (0.15; 0.40)**** |  | **0.24 (0.11; 0.34)**** |  | **0.24 (0.10; 0.35)**** |
| Ethnicity, Black/White |  | **-0.19 (-0.47; -0.03)*** |  | **-0.18 (-0.25; -0.11)**** |  | **-0.15 (-0.29; -0.02)*** |  | **-0.24 (-0.35; -0.10)**** |  | **-0.15 (-0.29; -0.01)*** |
| Protein intake, g |  | -0.01 (-0.19; 0.15) |  | 0.04 (-0.02; 0.10) |  | 0.02 (-0.09; 0.14) |  | 0.09 (-0.01; 0.18) |  | -0.01 (-0.11; 0.09) |
| Waist-to-height ratio |  | 0.05 (-0.15; 0.29) |  | 0.04 (-0.02; 0.11) |  | 0.10 (-0.01; 0.30) |  | 0.04 (-0.09; 0.17) |  | -0.05 (-0.13; 0.07) |
| Physical act, kCal/kg/day |  | 0.02 (-0.19; 0.27) |  | 0.03 (-0.02; 0.08) |  | 0.02 (-0.09; 0.15) |  | 0.05 (-0.04; 0.13) |  | 0.01 (-0.08; 0.10) |
| Cotinine, ng/ml |  |  |  | **-0.08 (-0.12; -0.02)*** |  | -0.03 (-0.20; 0.10) |  | -0.03 (-0.13; 0.06) |  | **-0.15 (-0.21; -0.02)*** |
| GGT, U/L |  | **-0.36 (-0.72; -0.26)**** |  | **-0.25 (-0.31; -0.18)**** |  | **-0.28 (-0.44; -0.18)**** |  | **-0.20 (-0.34; -0.10)**** |  | **-0.22 (-0.31; -0.09)**** |
| 24h Systolic BP, mmHg |  | -0.10 (-0.40; 0.12) |  | -0.02 (-0.08; 0.05) |  | 0.01 (-0.13; 0.15) |  | -0.03 (-0.14; 0.09) |  | -0.03 (-0.14; 0.09) |
| HbA1c, (%) |  | 0.01 (-0.19; 0.22) |  | -0.04 (-0.10; 0.02) |  | -0.02 (-0.15; 0.10) |  | -0.05 (-0.14; 0.05) |  | -0.05 (-0.15; 0.06) |
| LDL cholesterol, mmol/L |  | **-0.33 (-0.84; -0.31)**** |  | **-0.31 (-0.36; -0.24)**** |  | **-0.29 (-0.48; -0.22)**** |  | **-0.34 (-0.44; -0.23)**** |  | **-0.30 (-0.34; -0.14)**** |
| SES score |  | 0.05 (-0.21; 0.42) |  | <0.01 (-0.07; 0.06) |  | -0.07 (-0.22; 0.07) |  | 0.06 (-0.07; 0.18) |  | 0.01 (-0.12; 0.13) |
| Asparagine, AU | **0.41**** | 0.03 (-0.13; 0.19) | **0.32**** | **0.07 (0.02; 0.13)*** | **0.31**** | 0.02 (-0.09; 0.13) | **0.33**** | **0.11 (0.01; 0.19)*** | **0.24**** | 0.08 (-0.03; 0.17) |
| Age, years |  | -0.16 (-0.39; <0.01) |  | **-0.09 (-0.15; -0.02)*** |  | -0.09 (-0.21; 0.02) |  | **-0.12 (-0.22; -0.01)*** |  | -0.03 (-0.13; 0.09) |
| Sex, female/male |  | **0.34 (0.20; 0.62)**** |  | **0.25 (0.18; 0.31)**** |  | **0.27 (0.15; 0.40)**** |  | **0.22 (0.10; 0.33)**** |  | **0.23 (0.09; 0.33)**** |
| Ethnicity, Black/White |  | **-0.18 (-0.46; -0.03)*** |  | **-0.18 (-0.25; -0.10)**** |  | **-0.15 (-0.29; -0.02)*** |  | **-0.23 (-0.34; -0.10)**** |  | -0.15 (-0.28; <0.01) |
| Protein intake, g |  | -0.01 (-0.19; 0.16) |  | 0.04 (-0.02; 0.10) |  | 0.02 (-0.09; 0.14) |  | 0.09 (-0.01; 0.18) |  | <0.01 (-0.10; 0.10) |
| Waist-to-height ratio |  | 0.04 (-0.15; 0.29) |  | 0.04 (-0.02; 0.11) |  | 0.09 (-0.02; 0.30) |  | 0.04 (-0.08; 0.18) |  | -0.04 (-0.13; 0.07) |
| Physical act, kCal/kg/day |  | 0.02 (-0.19; 0.27) |  | 0.03 (-0.02; 0.08) |  | 0.02 (-0.10; 0.15) |  | 0.05 (-0.04; 0.13) |  | 0.01 (-0.08; 0.10) |
| Cotinine, ng/ml |  |  |  | **-0.07 (-0.12; -0.01)*** |  | -0.03 (-0.20; 0.10) |  | -0.03 (-0.12; 0.07) |  | **-0.15 (-0.21; -0.02)*** |
| GGT, U/L |  | **-0.36 (-0.73; -0.26)**** |  | **-0.24 (-0.31; -0.18)**** |  | **-0.27 (-0.44; -0.18)**** |  | **-0.19 (-0.33; -0.10)**** |  | **-0.22 (-0.30; -0.09)**** |
| 24h Systolic BP, mmHg |  | -0.09 (-0.39; 0.13) |  | -0.02 (-0.09; 0.05) |  | 0.01 (-0.12; 0.15) |  | -0.04 (-0.15; 0.08) |  | -0.03 (-0.14; 0.09) |
| HbA1c, (%) |  | 0.01 (-0.19; 0.22) |  | -0.04 (-0.10; 0.02) |  | -0.02 (-0.15; 0.10) |  | -0.05 (-0.14; 0.05) |  | -0.05 (-0.14; 0.06) |
| LDL cholesterol, mmol/L |  | **-0.34 (-0.84; -0.32)**** |  | **-0.31 (-0.35; -0.23)**** |  | **-0.29 (-0.48; -0.22)**** |  | **-0.34 (-0.44; -0.23)**** |  | **-0.30 (-0.33; -0.13)**** |
| SES score |  | 0.06 (-0.20; 0.43) |  | <0.01 (-0.07; 0.06) |  | -0.07 (-0.23; 0.06) |  | 0.06 (-0.07; 0.18) |  | <0.01 (-0.12; 0.12) |
| Glycine, AU | **0.41**** | 0.07 (-0.08; 0.25) | **0.32**** | **0.06 (<0.01; 0.12)*** | **0.31**** | 0.03 (-0.07; 0.14) | **0.33**** | **0.10 (<0.01; 0.19)*** | **0.23**** | 0.05 (-0.06; 0.15) |
| Age, years |  | **-0.16 (-0.39; -0.01)*** |  | **-0.09 (-0.14; -0.02)*** |  | -0.09 (-0.21; 0.02) |  | **-0.12 (-0.22; -0.01)*** |  | -0.02 (-0.13; 0.09) |
| Sex, female/male |  | **0.36 (0.22; 0.64)**** |  | **0.26 (0.18; 0.32)**** |  | **0.27 (0.16; 0.41)**** |  | **0.23 (0.10; 0.33)**** |  | **0.24 (0.09; 0.34)**** |
| Ethnicity, Black/White |  | **-0.18 (-0.45; -0.02)*** |  | **-0.18 (-0.25; -0.10)**** |  | **-0.15 (-0.28; -0.02)*** |  | **-0.22 (-0.34; -0.09)**** |  | **-0.15 (-0.28; <0.01)*** |
| Protein intake, g |  | -0.01 (-0.19; 0.15) |  | 0.04 (-0.02; 0.10) |  | 0.03 (-0.09; 0.14) |  | 0.09 (-0.01; 0.18) |  | -0.01 (-0.11; 0.10) |
| Waist-to-height ratio |  | 0.04 (-0.16; 0.28) |  | 0.04 (-0.02; 0.10) |  | 0.09 (-0.02; 0.30) |  | 0.03 (-0.09; 0.17) |  | -0.04 (-0.13; 0.07) |
| Physical act, kCal/kg/day |  | 0.03 (-0.18; 0.28) |  | 0.04 (-0.02; 0.09) |  | 0.02 (-0.10; 0.15) |  | 0.06 (-0.04; 0.14) |  | 0.02 (-0.08; 0.10) |
| Cotinine, ng/ml |  |  |  | **-0.08 (-0.13; -0.02)*** |  | -0.03 (-0.20; 0.10) |  | -0.04 (-0.13; 0.06) |  | **-0.16 (-0.22; -0.03)*** |
| GGT, U/L |  | **-0.35 (-0.71; -0.25)**** |  | **-0.24 (-0.31; -0.17)**** |  | **-0.27 (-0.44; -0.18)**** |  | **-0.19 (-0.33; -0.09)**** |  | **-0.22 (-0.31; -0.09)**** |
| 24h Systolic BP, mmHg |  | -0.09 (-0.38; 0.13) |  | -0.02 (-0.09; 0.05) |  | 0.01 (-0.12; 0.15) |  | -0.03 (-0.14; 0.09) |  | -0.03 (-0.14; 0.09) |
| HbA1c, (%) |  | 0.01 (-0.19; 0.23) |  | -0.04 (-0.10; 0.02) |  | -0.02 (-0.15; 0.10) |  | -0.04 (-0.14; 0.05) |  | -0.05 (-0.15; 0.06) |
| LDL cholesterol, mmol/L |  | **-0.34 (-0.84; -0.32)**** |  | **-0.31 (-0.36; -0.24)**** |  | **-0.29 (-0.47; -0.21)**** |  | **-0.34 (-0.44; -0.23)**** |  | **-0.31 (-0.34; -0.14)**** |
| SES score |  | 0.06 (-0.19; 0.43) |  | <0.01 (-0.07; 0.07) |  | -0.07 (-0.23; 0.06) |  | 0.07 (-0.06; 0.19) |  | 0.01 (-0.11; 0.13) |
| Serine, AU | **0.41**** | 0.01 (-0.16; 0.17) | **0.32**** | **0.07 (0.01; 0.12)*** | **0.31**** | 0.03 (-0.07; 0.13) | **0.33**** | 0.10 (<0.01; 0.18) | **0.24**** | 0.08 (-0.03; 0.18) |
| Age, years |  | **-0.16 (-0.39; <0.01)*** |  | **-0.08 (-0.14; -0.02)*** |  | -0.09 (-0.20; 0.02) |  | **-0.12 (-0.22; -0.01)*** |  | -0.02 (-0.13; 0.09) |
| Sex, female/male |  | **0.34 (0.20; 0.62)**** |  | **0.25 (0.18; 0.31)**** |  | **0.27 (0.15; 0.40)**** |  | **0.23 (0.10; 0.33)**** |  | **0.24 (0.09; 0.34)**** |
| Ethnicity, Black/White |  | **-0.18 (-0.46; -0.03)*** |  | **-0.17 (-0.24; -0.10)**** |  | **-0.15 (-0.28; -0.02)*** |  | **-0.23 (-0.34; -0.09)**** |  | **-0.15 (-0.28; <0.01)*** |
| Protein intake, g |  | -0.01 (-0.19; 0.16) |  | 0.04 (-0.02; 0.10) |  | 0.02 (-0.09; 0.14) |  | 0.09 (-0.01; 0.19) |  | <0.01 (-0.10; 0.10) |
| Waist-to-height ratio |  | 0.04 (-0.16; 0.28) |  | 0.05 (-0.02; 0.11) |  | 0.09 (-0.01; 0.30) |  | 0.04 (-0.08; 0.18) |  | -0.03 (-0.12; 0.07) |
| Physical act, kCal/kg/day |  | 0.03 (-0.19; 0.28) |  | 0.03 (-0.02; 0.08) |  | 0.02 (-0.10; 0.15) |  | 0.05 (-0.05; 0.13) |  | 0.01 (-0.08; 0.10) |
| Cotinine, ng/ml |  |  |  | **-0.07 (-0.12; -0.02)*** |  | -0.03 (-0.20; 0.10) |  | -0.03 (-0.12; 0.07) |  | **-0.15 (-0.21; -0.02)*** |
| GGT, U/L |  | **-0.36 (-0.73; -0.27)**** |  | **-0.24 (-0.31; -0.18)**** |  | **-0.27 (-0.44; -0.18)**** |  | **-0.20 (-0.33; -0.10)**** |  | **-0.21 (-0.30; -0.09)**** |
| 24h Systolic BP, mmHg |  | -0.09 (-0.39; 0.13) |  | -0.02 (-0.09; 0.05) |  | 0.01 (-0.12; 0.15) |  | -0.03 (-0.14; 0.09) |  | -0.03 (-0.15; 0.09) |
| HbA1c, (%) |  | 0.01 (-0.19; 0.22) |  | -0.04 (-0.10; 0.02) |  | -0.02 (-0.15; 0.10) |  | -0.04 (-0.13; 0.06) |  | -0.05 (-0.15; 0.06) |
| LDL cholesterol, mmol/L |  | **-0.34 (-0.85; -0.32)**** |  | **-0.31 (-0.36; -0.24)**** |  | **-0.29 (-0.47; -0.21)**** |  | **-0.34 (-0.44; -0.23)**** |  | **-0.30 (-0.34; -0.14)**** |
| SES score |  | 0.06 (-0.20; 0.43) |  | <0.01 (-0.07; 0.07) |  | -0.07 (-0.23; 0.06) |  | 0.06 (-0.07; 0.18) |  | 0.01 (-0.12; 0.13) |

**Supplementary Table 2B. Multi-variable adjusted regression analysis with estimated glomerular filtration rate (cystatin C-based) as the dependent variable, with the metabolomics data in control, cardiovascular disease risk group and cardiovascular disease risk clusters**

Test used: Multiple linear regressions. Data are presented as adjusted R^2^ with β coefficient and 95% confidence intervals. Estimated glomerular filtration rate (cystatin C-based), adjusted for age, sex, ethnicity, protein intake, waist-to-hight ratio, physical activity, cotinine, GGT, 24h systolic BP, HbA1c, LDL, SES score. Bold values denote P≤0.05; *P≤0.05; **P≤0.001. Cardiovascular disease risk group criteria: Obese - ≥0.55 waist-to-height ratio; Physically inactive - <600 METs for moderate and/or vigorous intensity physical activity; Smoking - ≥11 ng/mL cotinine & self-reported smoking; Excessive alcohol intake - ≥49 U/L GGT & self-reported drinking; Masked hypertensive – normal clinic BP & 24h/day/night BP classified as hypertensive; Hyperglycemic - ≥5.7% HbA1c; Dyslipidemic - >3.4 mmol/L LDL; Low socio-economic – low SES.

*Abbreviations*: AU, arbitrary units; physical act, physical activity; GGT, gamma-glutamyl transferase; BP, blood pressure; HbA1c, glycated haemoglobin; LDL, low density lipoprotein; SES, socio-economic status; GFR, estimated glomerular filtration rate; CVD, cardiovascular disease.

**Supplementary Table 2C. Multi-variable adjusted regression analysis with estimated glomerular filtration rate (cystatin C-based) as the dependent variable, with the metabolomics data in control, cardiovascular disease risk group and cardiovascular disease risk clusters**

|  | **eGFR (cystatin-C), ml/min/1.73m^2^** | | | | | | | | | |
| --- | --- | --- | --- | --- | --- | --- | --- | --- | --- | --- |
|  | **Control group**  **(N=166)** | | **CVD risk group**  **(N=1036)** | | **1 CVD risk factor**  **(N=344)** | | **2 CVD risk factors (N=360)** | | **3+ CVD risk factors (N=332)** | |
| ***Metabolomic data*** | **Adj R^2^** | **β (95%Cl)** | **Adj R^2^** | **β (95%Cl)** | **Adj R^2^** | **β (95%Cl)** | **Adj R^2^** | **β (95%Cl)** | **Adj R^2^** | **β (95%Cl)** |
| Glutamine, AU | **0.41**** | 0.04 (-0.12; 0.20) | **0.32**** | **0.09 (0.03; 0.14)*** | **0.31**** | 0.05 (-0.05; 0.15) | **0.34**** | **0.14 (0.04; 0.22)*** | **0.24**** | 0.07 (-0.03; 0.17) |
| Age, years |  | **-0.16 (-0.39; <0.01)*** |  | **-0.09 (-0.15; -0.02)*** |  | -0.09 (-0.20; 0.02) |  | **-0.12 (-0.22; -0.01)*** |  | -0.03 (-0.13; 0.08) |
| Sex, female/male |  | **0.35 (0.20; 0.62)**** |  | **0.25 (0.17; 0.31)**** |  | **0.27 (0.15; 0.40)**** |  | **0.22 (0.10; 0.32)**** |  | **0.23 (0.09; 0.34)**** |
| Ethnicity, Black/White |  | **-0.18 (-0.46; -0.02)*** |  | **-0.17 (-0.24; -0.10)**** |  | **-0.14 (-0.28; -0.01)*** |  | **-0.22 (-0.34; -0.09)**** |  | **-0.15 (-0.28; <0.01)*** |
| Protein intake, g |  | -0.01 (-0.19; 0.15) |  | 0.04 (-0.01; 0.10) |  | 0.03 (-0.09; 0.14) |  | 0.09 (-0.01; 0.18) |  | <0.01 (-0.10; 0.10) |
| Waist-to-height ratio |  | 0.04 (-0.15; 0.28) |  | 0.05 (-0.02; 0.11) |  | 0.09 (-0.02; 0.30) |  | 0.04 (-0.08; 0.18) |  | -0.04 (-0.13; 0.07) |
| Physical act, kCal/kg/day |  | 0.03 (-0.19; 0.28) |  | 0.03 (-0.02; 0.08) |  | 0.02 (-0.10; 0.15) |  | 0.05 (-0.04; 0.13) |  | 0.01 (-0.08; 0.10) |
| Cotinine, ng/ml |  |  |  | **-0.07 (-0.12; -0.01)*** |  | -0.03 (-0.19; 0.11) |  | -0.03 (-0.12; 0.07) |  | **-0.15 (-0.21; -0.02)*** |
| GGT, U/L |  | **-0.36 (-0.72; -0.26)**** |  | **-0.24 (-0.31; -0.18)**** |  | **-0.27 (-0.44; -0.18)**** |  | **-0.20 (-0.33; -0.10)**** |  | **-0.21 (-0.30; -0.09)**** |
| 24h Systolic BP, mmHg |  | -0.09 (-0.39; 0.12) |  | -0.02 (-0.09; 0.05) |  | 0.01 (-0.12; 0.15) |  | -0.03 (-0.15; 0.08) |  | -0.03 (-0.14; 0.09) |
| HbA1c, (%) |  | 0.01 (-0.19; 0.22) |  | -0.04 (-0.10; 0.02) |  | -0.02 (-0.15; 0.10) |  | -0.04 (-0.13; 0.06) |  | -0.05 (-0.15; 0.06) |
| LDL cholesterol, mmol/L |  | **-0.34 (-0.84; -0.32)**** |  | **-0.31 (-0.35; -0.23)**** |  | **-0.28 (-0.47; -0.21)**** |  | **-0.33 (-0.43; -0.22)**** |  | **-0.30 (-0.34; -0.14)**** |
| SES score |  | 0.06 (-0.20; 0.43) |  | <0.01 (-0.07; 0.07) |  | -0.07 (-0.23; 0.06) |  | 0.07 (-0.06; 0.19) |  | 0.01 (-0.12; 0.13) |
| Isothreonine, AU | -0.26 | -0.05 (-0.93; 0.81) | **0.27**** | -0.03 (-0.16; 0.10) | 0.17 | -0.15 (-0.40; 0.12) | **0.21*** | 0.13 (-0.11; 0.39) | 0.09 | -0.14 (-0.41; 0.14) |
| Age, years |  | -0.15 (-1.02; 0.67) |  | -0.09 (-0.22; 0.06) |  | -0.10 (-0.39; 0.18) |  | -0.08 (-0.34; 0.18) |  | -0.01 (-0.26; 0.25) |
| Sex, female/male |  | 0.33 (-0.53; 1.31) |  | **0.25 (0.09; 0.39)*** |  | 0.23 (-0.09; 0.56) |  | 0.20 (-0.07; 0.46) |  | 0.23 (-0.08; 0.50) |
| Ethnicity, Black/White |  | -0.18 (-1.15; 0.67) |  | **-0.19 (-0.35; -0.02)*** |  | -0.17 (-0.52; 0.16) |  | -0.22 (-0.51; 0.08) |  | -0.21 (-0.54; 0.15) |
| Protein intake, g |  | <0.01 (-0.73; 0.72) |  | 0.04 (-0.09; 0.17) |  | 0.04 (-0.25; 0.33) |  | 0.10 (-0.14; 0.32) |  | -0.04 (-0.27; 0.21) |
| Waist-to-height ratio |  | 0.03 (-0.91; 1.00) |  | 0.04 (-0.10; 0.18) |  | 0.10 (-0.24; 0.55) |  | 0.03 (-0.27; 0.34) |  | -0.07 (-0.28; 0.18) |
| Physical act, kCal/kg/day |  | 0.02 (-0.94; 1.02) |  | 0.04 (-0.08; 0.15) |  | 0.05 (-0.26; 0.38) |  | 0.04 (-0.17; 0.24) |  | 0.03 (-0.19; 0.23) |
| Cotinine, ng/ml |  |  |  | -0.08 (-0.20; 0.05) |  | -0.06 (-0.48; 0.29) |  | -0.03 (-0.25; 0.20) |  | -0.18 (-0.37; 0.08) |
| GGT, U/L |  | -0.38 (-1.54; 0.49) |  | **-0.26 (-0.41; -0.11)**** |  | -0.26 (-0.62; 0.04) |  | -0.19 (-0.49; 0.06) |  | -0.28 (-0.52; 0.02) |
| 24h Systolic BP, mmHg |  | -0.08 (-1.21; 0.98) |  | -0.01 (-0.16; 0.14) |  | -0.01 (-0.36; 0.34) |  | -0.01 (-0.29; 0.26) |  | 0.02 (-0.27; 0.30) |
| HbA1c, (%) |  | 0.01 (-0.86; 0.89) |  | -0.04 (-0.17; 0.09) |  | -0.04 (-0.36; 0.27) |  | -0.03 (-0.26; 0.20) |  | -0.08 (-0.31; 0.18) |
| LDL cholesterol, mmol/L |  | -0.34 (-1.67; 0.52) |  | **-0.32 (-0.43; -0.17)**** |  | -0.34 (-0.74; -0.06)* |  | **-0.35 (-0.60; -0.09)*** |  | -0.28 (-0.46; 0.02) |
| SES score |  | 0.04 (-1.35; 1.50) |  | <0.01 (-0.15; 0.15) |  | <0.01 (-0.39; 0.40) |  | 0.04 (-0.26; 0.33) |  | 0.03 (-0.26; 0.31) |
| Dimethylglycine, AU | **0.41**** | 0.01 (-0.16; 0.19) | **0.32**** | **0.06 (<0.01; 0.11)*** | **0.31**** | 0.05 (-0.06; 0.16) | **0.32**** | 0.03 (-0.06; 0.12) | **0.24**** | 0.10 (-0.01; 0.19) |
| Age, years |  | -0.16 (-0.39; 0.01) |  | **-0.09 (-0.15; -0.02)*** |  | -0.09 (-0.21; 0.02) |  | **-0.12 (-0.23; -0.01)*** |  | -0.03 (-0.14; 0.08) |
| Sex, female/male |  | **0.34 (0.20; 0.62)**** |  | **0.25 (0.17; 0.31)**** |  | **0.27 (0.15; 0.40)**** |  | **0.21 (0.09; 0.32)**** |  | **0.22 (0.08; 0.33)**** |
| Ethnicity, Black/White |  | **-0.18 (-0.46; -0.03)*** |  | **-0.18 (-0.25; -0.10)**** |  | **-0.15 (-0.29; -0.02)*** |  | **-0.23 (-0.34; -0.09)**** |  | **-0.16 (-0.30; -0.01)*** |
| Protein intake, g |  | -0.01 (-0.19; 0.16) |  | 0.04 (-0.02; 0.10) |  | 0.03 (-0.09; 0.14) |  | 0.09 (-0.01; 0.18) |  | -0.01 (-0.11; 0.09) |
| Waist-to-height ratio |  | 0.04 (-0.16; 0.28) |  | 0.04 (-0.02; 0.11) |  | 0.10 (-0.01; 0.31) |  | 0.03 (-0.09; 0.17) |  | -0.04 (-0.13; 0.07) |
| Physical act, kCal/kg/day |  | 0.03 (-0.19; 0.28) |  | 0.03 (-0.02; 0.08) |  | 0.02 (-0.09; 0.15) |  | 0.05 (-0.05; 0.13) |  | 0.01 (-0.08; 0.10) |
| Cotinine, ng/ml |  |  |  | **-0.08 (-0.13; -0.02)*** |  | -0.03 (-0.20; 0.10) |  | -0.03 (-0.12; 0.07) |  | **-0.14 (-0.21; -0.02)*** |
| GGT, U/L |  | **-0.36 (-0.73; -0.27)**** |  | **-0.25 (-0.31; -0.18)**** |  | **-0.27 (-0.44; -0.17)**** |  | **-0.20 (-0.34; -0.11)**** |  | **-0.22 (-0.31; -0.09)**** |
| 24h Systolic BP, mmHg |  | -0.09 (-0.39; 0.13) |  | -0.02 (-0.08; 0.05) |  | 0.01 (-0.13; 0.15) |  | -0.01 (-0.13; 0.11) |  | -0.02 (-0.13; 0.10) |
| HbA1c, (%) |  | 0.01 (-0.20; 0.23) |  | -0.04 (-0.1; 0.02) |  | -0.02 (-0.15; 0.10) |  | -0.04 (-0.14; 0.06) |  | -0.06 (-0.15; 0.06) |
| LDL cholesterol, mmol/L |  | **-0.34 (-0.85; -0.32)**** |  | **-0.31 (-0.36; -0.24)**** |  | **-0.29 (-0.48; -0.21)**** |  | **-0.35 (-0.45; -0.23)**** |  | **-0.29 (-0.33; -0.13)**** |
| SES score |  | 0.06 (-0.20; 0.43) |  | -0.01 (-0.07; 0.06) |  | -0.07 (-0.23; 0.07) |  | 0.04 (-0.09; 0.17) |  | <0.01 (-0.12; 0.13) |
| Beta-alanine, AU | **0.41**** | -0.01 (-0.19; 0.16) | **0.31**** | 0.02 (-0.03; 0.08) | **0.31**** | 0.03 (-0.07; 0.13) | **0.32**** | 0.06 (-0.03; 0.16) | **0.23**** | -0.03 (-0.12; 0.07) |
| Age, years |  | -0.16 (-0.39; 0.01) |  | **-0.08 (-0.14; -0.02)*** |  | -0.09 (-0.21; 0.02) |  | **-0.12 (-0.22; <0.01)*** |  | -0.02 (-0.13; 0.09) |
| Sex, female/male |  | **0.34 (0.20; 0.62)**** |  | **0.25 (0.18; 0.31)**** |  | **0.27 (0.15; 0.40)**** |  | **0.22 (0.09; 0.32)**** |  | **0.22 (0.08; 0.33)**** |
| Ethnicity, Black/White |  | **-0.18 (-0.46; -0.03)*** |  | **-0.18 (-0.25; -0.10)**** |  | **-0.15 (-0.29; -0.02)*** |  | **-0.23 (-0.35; -0.10)**** |  | **-0.16 (-0.30; -0.01)*** |
| Protein intake, g |  | -0.01 (-0.19; 0.16) |  | 0.04 (-0.02; 0.10) |  | 0.02 (-0.09; 0.14) |  | 0.08 (-0.02; 0.18) |  | -0.01 (-0.11; 0.09) |
| Waist-to-height ratio |  | 0.04 (-0.16; 0.28) |  | 0.04 (-0.02; 0.10) |  | 0.09 (-0.02; 0.30) |  | 0.03 (-0.09; 0.17) |  | -0.05 (-0.13; 0.06) |
| Physical act, kCal/kg/day |  | 0.03 (-0.19; 0.28) |  | 0.03 (-0.02; 0.08) |  | 0.02 (-0.10; 0.15) |  | 0.06 (-0.04; 0.14) |  | 0.01 (-0.08; 0.10) |
| Cotinine, ng/ml |  |  |  | **-0.08 (-0.13; -0.02)*** |  | -0.03 (-0.20; 0.10) |  | -0.03 (-0.12; 0.07) |  | **-0.15 (-0.21; -0.02)*** |
| GGT, U/L |  | **-0.37 (-0.73; -0.27)**** |  | **-0.25 (-0.32; -0.19)**** |  | **-0.28 (-0.44; -0.18)**** |  | **-0.21 (-0.34; -0.11)**** |  | **-0.23 (-0.31; -0.10)**** |
| 24h Systolic BP, mmHg |  | -0.09 (-0.39; 0.13) |  | -0.01 (-0.08; 0.05) |  | 0.01 (-0.12; 0.15) |  | -0.02 (-0.14; 0.10) |  | -0.02 (-0.13; 0.10) |
| HbA1c, (%) |  | 0.01 (-0.19; 0.23) |  | -0.04 (-0.10; 0.02) |  | -0.02 (-0.15; 0.10) |  | -0.04 (-0.14; 0.06) |  | -0.05 (-0.15; 0.06) |
| LDL cholesterol, mmol/L |  | **-0.34 (-0.85; -0.33)**** |  | **-0.32 (-0.36; -0.24)**** |  | **-0.29 (-0.48; -0.22)**** |  | **-0.34 (-0.44; -0.23)**** |  | **-0.31 (-0.34; -0.14)**** |
| SES score |  | 0.06 (-0.20; 0.43) |  | <0.01 (-0.07; 0.06) |  | -0.07 (-0.22; 0.07) |  | 0.06 (-0.07; 0.18) |  | 0.01 (-0.11; 0.13) |

Test used: Multiple linear regressions. Data are presented as adjusted R^2^ with β coefficient and 95% confidence intervals. Estimated glomerular filtration rate (cystatin C-based), adjusted for age, sex, ethnicity, protein intake, waist-to-hight ratio, physical activity, cotinine, GGT, 24h systolic BP, HbA1c, LDL, SES score. Bold values denote P≤0.05; *P≤0.05; **P≤0.001. Cardiovascular disease risk group criteria: Obese - ≥0.55 waist-to-height ratio; Physically inactive - <600 METs for moderate and/or vigorous intensity physical activity; Smoking - ≥11 ng/mL cotinine & self-reported smoking; Excessive alcohol intake - ≥49 U/L GGT & self-reported drinking; Masked hypertensive – normal clinic BP & 24h/day/night BP classified as hypertensive; Hyperglycemic - ≥5.7% HbA1c; Dyslipidemic - >3.4 mmol/L LDL; Low socio-economic – low SES.

*Abbreviations*: AU, arbitrary units; physical act, physical activity; GGT, gamma-glutamyl transferase; BP, blood pressure; HbA1c, glycated haemoglobin; LDL, low density lipoprotein; SES, socio-economic status; GFR, estimated glomerular filtration rate; CVD, cardiovascular disease.

**Supplementary Table 2D. Multi-variable adjusted regression analysis with estimated glomerular filtration rate (cystatin C-based) as the dependent variable, with the metabolomics data in control, cardiovascular disease risk group and cardiovascular disease risk clusters**

|  | **eGFR (cystatin-C), ml/min/1.73m^2^** | | | | | | | | | |
| --- | --- | --- | --- | --- | --- | --- | --- | --- | --- | --- |
|  | **Control group**  **(N=166)** | | **CVD risk group**  **(N=1036)** | | **1 CVD risk factor**  **(N=344)** | | **2 CVD risk factors (N=360)** | | **3+ CVD risk factors (N=332)** | |
| ***Metabolomic data*** | **Adj R^2^** | **β (95%Cl)** | **Adj R^2^** | **β (95%Cl)** | **Adj R^2^** | **β (95%Cl)** | **Adj R^2^** | **β (95%Cl)** | **Adj R^2^** | **β (95%Cl)** |
| Threonine, AU | **0.41**** | 0.01 (-0.15; 0.18) | **0.32**** | **0.07 (0.01; 0.12)*** | **0.31**** | 0.04 (-0.06; 0.14) | **0.33**** | **0.10 (<0.01; 0.18)*** | **0.23**** | 0.06 (-0.05; 0.16) |
| Age, years |  | **-0.16 (-0.39; <0.01)*** |  | **-0.08 (-0.14; -0.02)*** |  | -0.09 (-0.20; 0.02) |  | **-0.12 (-0.22; -0.01)*** |  | -0.02 (-0.13; 0.09) |
| Sex, female/male |  | **0.34 (0.20; 0.62)**** |  | **0.25 (0.18; 0.31)**** |  | **0.27 (0.15; 0.40)**** |  | **0.22 (0.10; 0.33)**** |  | **0.23 (0.09; 0.34)**** |
| Ethnicity, Black/White |  | **-0.18 (-0.46; -0.03)*** |  | **-0.18 (-0.25; -0.10)**** |  | **-0.15 (-0.29; -0.02)*** |  | **-0.23 (-0.34; -0.10)**** |  | **-0.15 (-0.29; <0.01)*** |
| Protein intake, g |  | -0.01 (-0.19; 0.15) |  | 0.04 (-0.02; 0.10) |  | 0.02 (-0.09; 0.14) |  | 0.09 (-0.01; 0.18) |  | <0.01 (-0.11; 0.10) |
| Waist-to-height ratio |  | 0.04 (-0.16; 0.28) |  | 0.04 (-0.02; 0.11) |  | 0.09 (-0.02; 0.30) |  | 0.04 (-0.08; 0.18) |  | -0.04 (-0.13; 0.07) |
| Physical act, kCal/kg/day |  | 0.03 (-0.19; 0.28) |  | 0.03 (-0.02; 0.09) |  | 0.02 (-0.10; 0.15) |  | 0.05 (-0.04; 0.13) |  | 0.01 (-0.08; 0.10) |
| Cotinine, ng/ml |  |  |  | **-0.07 (-0.12; -0.01)*** |  | -0.03 (-0.19; 0.11) |  | -0.03 (-0.12; 0.07) |  | **-0.15 (-0.21; -0.02)*** |
| GGT, U/L |  | **-0.36 (-0.73; -0.27)**** |  | **-0.24 (-0.31; -0.18)**** |  | **-0.27 (-0.44; -0.17)**** |  | **-0.20 (-0.33; -0.10)**** |  | **-0.22 (-0.30; -0.09)**** |
| 24h Systolic BP, mmHg |  | -0.09 (-0.39; 0.13) |  | -0.02 (-0.09; 0.05) |  | 0.01 (-0.12; 0.15) |  | -0.03 (-0.15; 0.09) |  | -0.03 (-0.14; 0.09) |
| HbA1c, (%) |  | 0.01 (-0.19; 0.22) |  | -0.04 (-0.10; 0.02) |  | -0.02 (-0.15; 0.10) |  | -0.04 (-0.14; 0.05) |  | -0.05 (-0.15; 0.06) |
| LDL cholesterol, mmol/L |  | **-0.34 (-0.85; -0.32)**** |  | **-0.31 (-0.35; -0.23)**** |  | **-0.29 (-0.47; -0.21)**** |  | **-0.34 (-0.44; -0.23)**** |  | **-0.30 (-0.34; -0.14)**** |
| SES score |  | 0.06 (-0.20; 0.43) |  | <0.01 (-0.07; 0.07) |  | -0.07 (-0.23; 0.06) |  | 0.06 (-0.07; 0.18) |  | 0.01 (-0.12; 0.13) |
| Hydroxyproline, AU | -0.26 | -0.04 (-1.08; 0.98) | **0.28**** | -0.03 (-0.14; 0.09) | **0.26*** | **-0.36 (-0.64; -0.07)*** | **0.23*** | 0.12 (-0.10; 0.32) | 0.10 | -0.05 (-0.28; 0.20) |
| Age, years |  | -0.16 (-1.01; 0.64) |  | -0.08 (-0.21; 0.05) |  | -0.14 (-0.41; 0.13) |  | -0.13 (-0.36; 0.11) |  | -0.01 (-0.25; 0.24) |
| Sex, female/male |  | 0.32 (-0.61; 1.38) |  | **0.25 (0.10; 0.38)**** |  | 0.27 (-0.02; 0.57) |  | 0.21 (-0.04; 0.45) |  | 0.22 (-0.07; 0.48) |
| Ethnicity, Black/White |  | -0.18 (-1.16; 0.68) |  | **-0.19 (-0.35; -0.03)*** |  | -0.19 (-0.51; 0.13) |  | -0.19 (-0.46; 0.09) |  | -0.17 (-0.48; 0.15) |
| Protein intake, g |  | -0.01 (-0.74; 0.72) |  | 0.04 (-0.09; 0.16) |  | -0.04 (-0.32; 0.23) |  | 0.09 (-0.12; 0.30) |  | -0.01 (-0.23; 0.21) |
| Waist-to-height ratio |  | 0.04 (-0.86; 0.98) |  | 0.05 (-0.09; 0.18) |  | 0.26 (-0.03; 0.81) |  | 0.01 (-0.26; 0.30) |  | -0.05 (-0.25; 0.18) |
| Physical act, kCal/kg/day |  | 0.04 (-0.98; 1.10) |  | 0.04 (-0.08; 0.15) |  | 0.12 (-0.16; 0.46) |  | 0.05 (-0.14; 0.24) |  | 0.02 (-0.18; 0.21) |
| Cotinine, ng/ml |  |  |  | -0.08 (-0.19; 0.04) |  | -0.13 (-0.56; 0.18) |  | -0.05 (-0.26; 0.16) |  | -0.16 (-0.33; 0.08) |
| GGT, U/L |  | -0.35 (-1.57; 0.62) |  | **-0.26 (-0.40; -0.12)**** |  | **-0.36 (-0.73; -0.09)*** |  | -0.18 (-0.45; 0.06) |  | -0.24 (-0.46; 0.02) |
| 24h Systolic BP, mmHg |  | -0.08 (-1.21; 0.97) |  | -0.01 (-0.15; 0.14) |  | -0.04 (-0.37; 0.29) |  | -0.02 (-0.27; 0.24) |  | <0.01 (-0.27; 0.27) |
| HbA1c, (%) |  | <0.01 (-0.93; 0.93) |  | -0.04 (-0.17; 0.09) |  | -0.04 (-0.34; 0.24) |  | -0.04 (-0.25; 0.17) |  | -0.06 (-0.28; 0.18) |
| LDL cholesterol, mmol/L |  | -0.35 (-1.70; 0.51) |  | **-0.32 (-0.43; -0.17)**** |  | **-0.28 (-0.64; -0.03)*** |  | **-0.35 (-0.57; -0.11)*** |  | -0.30 (-0.46; -0.02)* |
| SES score |  | 0.04 (-1.35; 1.53) |  | <0.01 (-0.15; 0.14) |  | -0.03 (-0.39; 0.30) |  | 0.04 (-0.23; 0.31) |  | 0.02 (-0.25; 0.28) |
| Alanine, AU | **0.41**** | 0.07 (-0.08; 0.24) | **0.32**** | **0.08 (0.03; 0.14)*** | **0.31**** | 0.05 (-0.06; 0.15) | **0.34**** | **0.15 (0.05; 0.23)*** | **0.23**** | 0.05 (-0.06; 0.16) |
| Age, years |  | -0.16 (-0.38; <0.01) |  | **-0.09 (-0.14; -0.02)*** |  | -0.09 (-0.20; 0.02) |  | **-0.12 (-0.22; <0.01)*** |  | -0.02 (-0.13; 0.09) |
| Sex, female/male |  | **0.35 (0.21; 0.62)**** |  | **0.24 (0.17; 0.31)**** |  | **0.27 (0.15; 0.40)**** |  | **0.21 (0.09; 0.31)**** |  | **0.23 (0.09; 0.34)**** |
| Ethnicity, Black/White |  | **-0.18 (-0.46; -0.03)*** |  | **-0.18 (-0.25; -0.10)**** |  | **-0.15 (-0.28; -0.02)*** |  | **-0.23 (-0.34; -0.10)**** |  | **-0.15 (-0.28; <0.01)*** |
| Protein intake, g |  | -0.01 (-0.18; 0.16) |  | 0.04 (-0.02; 0.10) |  | 0.02 (-0.09; 0.14) |  | 0.09 (-0.01; 0.18) |  | <0.01 (-0.11; 0.10) |
| Waist-to-height ratio |  | 0.05 (-0.15; 0.29) |  | 0.04 (-0.02; 0.10) |  | 0.09 (-0.02; 0.29) |  | 0.04 (-0.08; 0.17) |  | -0.04 (-0.13; 0.07) |
| Physical act, kCal/kg/day |  | 0.02 (-0.19; 0.27) |  | 0.04 (-0.02; 0.09) |  | 0.02 (-0.10; 0.15) |  | 0.05 (-0.04; 0.13) |  | 0.01 (-0.08; 0.10) |
| Cotinine, ng/ml |  |  |  | **-0.07 (-0.12; -0.01)*** |  | -0.03 (-0.19; 0.11) |  | -0.03 (-0.12; 0.07) |  | **-0.15 (-0.21; -0.03)*** |
| GGT, U/L |  | **-0.36 (-0.72; -0.26)**** |  | **-0.24 (-0.31; -0.18)**** |  | **-0.27 (-0.44; -0.17)**** |  | **-0.19 (-0.33; -0.10)**** |  | **-0.22 (-0.31; -0.09)**** |
| 24h Systolic BP, mmHg |  | -0.10 (-0.41; 0.11) |  | -0.02 (-0.09; 0.05) |  | 0.01 (-0.12; 0.15) |  | -0.04 (-0.15; 0.08) |  | -0.03 (-0.15; 0.09) |
| HbA1c, (%) |  | 0.01 (-0.19; 0.22) |  | -0.04 (-0.1; 0.02) |  | -0.02 (-0.15; 0.10) |  | -0.04 (-0.13; 0.06) |  | -0.05 (-0.15; 0.06) |
| LDL cholesterol, mmol/L |  | **-0.33 (-0.83; -0.31)**** |  | **-0.31 (-0.35; -0.23)**** |  | **-0.29 (-0.47; -0.21)**** |  | **-0.33 (-0.43; -0.22)**** |  | **-0.31 (-0.34; -0.14)**** |
| SES score |  | 0.06 (-0.19; 0.43) |  | <0.01 (-0.07; 0.07) |  | -0.07 (-0.23; 0.06) |  | 0.07 (-0.06; 0.19) |  | 0.01 (-0.12; 0.13) |
| Citrulline, AU | **0.39**** | <0.01 (-0.17; 0.18) | **0.31**** | 0.04 (-0.02; 0.10) | **0.31**** | -0.02 (-0.14; 0.10) | **0.32**** | 0.08 (-0.03; 0.17) | **0.23**** | 0.06 (-0.05; 0.18) |
| Age, years |  | -0.16 (-0.41; 0.02) |  | **-0.09 (-0.15; -0.02)*** |  | -0.09 (-0.22; 0.04) |  | **-0.13 (-0.24; <0.01)*** |  | -0.03 (-0.15; 0.10) |
| Sex, female/male |  | **0.34 (0.18; 0.64)**** |  | **0.25 (0.17; 0.32)**** |  | **0.27 (0.14; 0.42)**** |  | **0.22 (0.09; 0.34)**** |  | **0.23 (0.08; 0.35)*** |
| Ethnicity, Black/White |  | **-0.18 (-0.49; <0.01)*** |  | **-0.18 (-0.26; -0.09)**** |  | **-0.15 (-0.31; -0.01)*** |  | **-0.23 (-0.36; -0.08)*** |  | -0.15 (-0.30; 0.02) |
| Protein intake, g |  | -0.01 (-0.21; 0.17) |  | 0.04 (-0.03; 0.10) |  | 0.02 (-0.10; 0.15) |  | 0.08 (-0.03; 0.19) |  | <0.01 (-0.12; 0.11) |
| Waist-to-height ratio |  | 0.04 (-0.18; 0.31) |  | 0.04 (-0.03; 0.11) |  | 0.10 (-0.03; 0.32) |  | 0.04 (-0.10; 0.19) |  | -0.04 (-0.14; 0.08) |
| Physical act, kCal/kg/day |  | 0.03 (-0.22; 0.30) |  | 0.03 (-0.03; 0.09) |  | 0.03 (-0.11; 0.17) |  | 0.05 (-0.05; 0.14) |  | 0.01 (-0.09; 0.11) |
| Cotinine, ng/ml |  |  |  | **-0.08 (-0.13; -0.01)*** |  | -0.04 (-0.22; 0.11) |  | -0.03 (-0.13; 0.08) |  | **-0.15 (-0.23; -0.02)*** |
| GGT, U/L |  | **-0.36 (-0.76; -0.24)**** |  | **-0.25 (-0.32; -0.17)**** |  | **-0.28 (-0.46; -0.17)**** |  | **-0.20 (-0.35; -0.09)**** |  | **-0.22 (-0.32; -0.08)**** |
| 24h Systolic BP, mmHg |  | -0.09 (-0.41; 0.16) |  | -0.02 (-0.09; 0.06) |  | 0.01 (-0.14; 0.16) |  | -0.02 (-0.15; 0.11) |  | -0.04 (-0.16; 0.10) |
| HbA1c, (%) |  | 0.01 (-0.22; 0.25) |  | -0.04 (-0.10; 0.03) |  | -0.02 (-0.16; 0.11) |  | -0.04 (-0.15; 0.07) |  | -0.05 (-0.16; 0.08) |
| LDL cholesterol, mmol/L |  | **-0.34 (-0.88; -0.29)**** |  | **-0.32 (-0.36; -0.23)**** |  | **-0.30 (-0.50; -0.21)**** |  | **-0.34 (-0.46; -0.22)**** |  | **-0.31 (-0.35; -0.13)**** |
| SES score |  | 0.06 (-0.23; 0.47) |  | <0.01 (-0.08; 0.07) |  | -0.07 (-0.24; 0.08) |  | 0.06 (-0.09; 0.20) |  | 0.01 (-0.12; 0.15) |

Test used: Multiple linear regressions. Data are presented as adjusted R^2^ with β coefficient and 95% confidence intervals. Estimated glomerular filtration rate (cystatin C-based), adjusted for age, sex, ethnicity, protein intake, waist-to-hight ratio, physical activity, cotinine, GGT, 24h systolic BP, HbA1c, LDL, SES score. Bold values denote P≤0.05; *P≤0.05; **P≤0.001. Cardiovascular disease risk group criteria: Obese - ≥0.55 waist-to-height ratio; Physically inactive - <600 METs for moderate and/or vigorous intensity physical activity; Smoking - ≥11 ng/mL cotinine & self-reported smoking; Excessive alcohol intake - ≥49 U/L GGT & self-reported drinking; Masked hypertensive – normal clinic BP & 24h/day/night BP classified as hypertensive; Hyperglycemic - ≥5.7% HbA1c; Dyslipidemic - >3.4 mmol/L LDL; Low socio-economic – low SES.

*Abbreviations*: AU, arbitrary units; physical act, physical activity; GGT, gamma-glutamyl transferase; BP, blood pressure; HbA1c, glycated haemoglobin; LDL, low density lipoprotein; SES, socio-economic status; GFR, estimated glomerular filtration rate; CVD, cardiovascular disease.

**Supplementary Table 2E. Multi-variable adjusted regression analysis with estimated glomerular filtration rate (cystatin C-based) as the dependent variable, with the metabolomics data in control, cardiovascular disease risk group and cardiovascular disease risk clusters**

|  | **eGFR (cystatin-C), ml/min/1.73m^2^** | | | | | | | | | |
| --- | --- | --- | --- | --- | --- | --- | --- | --- | --- | --- |
|  | **Control group**  **(N=166)** | | **CVD risk group**  **(N=1036)** | | **1 CVD risk factor**  **(N=344)** | | **2 CVD risk factors (N=360)** | | **3+ CVD risk factors (N=332)** | |
| ***Metabolomic data*** | **Adj R^2^** | **β (95%Cl)** | **Adj R^2^** | **β (95%Cl)** | **Adj R^2^** | **β (95%Cl)** | **Adj R^2^** | **β (95%Cl)** | **Adj R^2^** | **β (95%Cl)** |
| GABA, AU | **0.40**** | 0.01 (-0.18; 0.20) | **0.31**** | 0.05 (-0.01; 0.11) | **0.32**** | 0.09 (-0.02; 0.19) | **0.33**** | 0.10 (<0.01; 0.23) | **0.23**** | -0.04 (-0.13; 0.07) |
| Age, years |  | -0.16 (-0.40; 0.01) |  | **-0.09 (-0.15; -0.02)*** |  | -0.09 (-0.21; 0.03) |  | **-0.13 (-0.24; -0.01)*** |  | -0.02 (-0.13; 0.10) |
| Sex, female/male |  | **0.34 (0.18; 0.64)**** |  | **0.26 (0.18; 0.32)**** |  | **0.29 (0.16; 0.43)**** |  | **0.23 (0.10; 0.34)**** |  | **0.22 (0.07; 0.34)*** |
| Ethnicity, Black/White |  | **-0.18 (-0.48; -0.01)*** |  | **-0.19 (-0.26; -0.11)**** |  | **-0.17 (-0.31; -0.03)*** |  | **-0.25 (-0.37; -0.10)**** |  | **-0.15 (-0.29; <0.01)*** |
| Protein intake, g |  | -0.01 (-0.20; 0.17) |  | 0.05 (-0.02; 0.11) |  | 0.03 (-0.08; 0.16) |  | 0.11 (<0.01; 0.21) |  | -0.02 (-0.12; 0.09) |
| Waist-to-height ratio |  | 0.04 (-0.17; 0.30) |  | 0.05 (-0.02; 0.11) |  | 0.09 (-0.03; 0.30) |  | 0.04 (-0.09; 0.19) |  | -0.05 (-0.14; 0.07) |
| Physical act, kCal/kg/day |  | 0.03 (-0.21; 0.29) |  | 0.03 (-0.03; 0.09) |  | 0.02 (-0.11; 0.15) |  | 0.05 (-0.05; 0.14) |  | 0.01 (-0.08; 0.11) |
| Cotinine, ng/ml |  |  |  | **-0.08 (-0.13; -0.02)*** |  | -0.03 (-0.20; 0.12) |  | -0.04 (-0.13; 0.07) |  | **-0.15 (-0.22; -0.02)*** |
| GGT, U/L |  | **-0.36 (-0.74; -0.25)**** |  | **-0.25 (-0.32; -0.18)**** |  | **-0.27 (-0.44; -0.16)**** |  | **-0.20 (-0.34; -0.10)**** |  | **-0.23 (-0.32; -0.09)**** |
| 24h Systolic BP, mmHg |  | -0.09 (-0.40; 0.15) |  | -0.01 (-0.08; 0.06) |  | 0.02 (-0.12; 0.17) |  | -0.02 (-0.14; 0.11) |  | -0.02 (-0.14; 0.10) |
| HbA1c, (%) |  | 0.01 (-0.21; 0.24) |  | -0.04 (-0.10; 0.03) |  | -0.02 (-0.15; 0.11) |  | -0.04 (-0.14; 0.07) |  | -0.05 (-0.16; 0.06) |
| LDL cholesterol, mmol/L |  | **-0.34 (-0.87; -0.29)**** |  | **-0.32 (-0.36; -0.23)**** |  | **-0.28 (-0.47; -0.19)**** |  | **-0.34 (-0.45; -0.22)**** |  | **-0.31 (-0.35; -0.14)**** |
| SES score |  | 0.06 (-0.22; 0.45) |  | <0.01 (-0.07; 0.07) |  | -0.07 (-0.24; 0.07) |  | 0.06 (-0.08; 0.19) |  | 0.01 (-0.12; 0.14) |
| Creatine, AU | **0.41**** | 0.09 (-0.07; 0.28) | **0.32**** | **0.12 (0.06; 0.17)**** | **0.33**** | **0.13 (0.03; 0.24)*** | **0.34**** | **0.16 (0.06; 0.26)*** | **0.24**** | 0.07 (-0.05; 0.16) |
| Age, years |  | **-0.17 (-0.39; -0.01)*** |  | **-0.09 (-0.14; -0.02)*** |  | -0.09 (-0.20; 0.02) |  | **-0.12 (-0.22; -0.01)*** |  | -0.02 (-0.13; 0.09) |
| Sex, female/male |  | **0.37 (0.23; 0.66)**** |  | **0.30 (0.22; 0.36)**** |  | **0.31 (0.19; 0.44)**** |  | **0.29 (0.15; 0.39)**** |  | **0.26 (0.11; 0.38)**** |
| Ethnicity, Black/White |  | **-0.17 (-0.44; -0.01)*** |  | **-0.17 (-0.24; -0.10)**** |  | **-0.14 (-0.28; -0.01)*** |  | **-0.25 (-0.36; -0.12)**** |  | -0.15 (-0.28; <0.01) |
| Protein intake, g |  | -0.03 (-0.20; 0.14) |  | 0.03 (-0.03; 0.09) |  | 0.02 (-0.09; 0.13) |  | 0.07 (-0.02; 0.17) |  | -0.02 (-0.12; 0.08) |
| Waist-to-height ratio |  | 0.04 (-0.15; 0.28) |  | 0.04 (-0.03; 0.10) |  | 0.09 (-0.03; 0.28) |  | 0.03 (-0.09; 0.16) |  | -0.05 (-0.13; 0.06) |
| Physical act, kCal/kg/day |  | 0.02 (-0.21; 0.26) |  | 0.03 (-0.03; 0.08) |  | 0.01 (-0.11; 0.14) |  | 0.05 (-0.04; 0.13) |  | <0.01 (-0.09; 0.09) |
| Cotinine, ng/ml |  |  |  | **-0.07 (-0.12; -0.02)*** |  | -0.02 (-0.18; 0.11) |  | -0.03 (-0.12; 0.06) |  | **-0.15 (-0.21; -0.03)*** |
| GGT, U/L |  | **-0.36 (-0.72; -0.27)**** |  | **-0.26 (-0.32; -0.19)**** |  | **-0.28 (-0.44; -0.18)**** |  | **-0.22 (-0.36; -0.13)**** |  | **-0.22 (-0.31; -0.10)**** |
| 24h Systolic BP, mmHg |  | -0.10 (-0.4; 0.11) |  | -0.02 (-0.09; 0.05) |  | 0.01 (-0.12; 0.15) |  | -0.04 (-0.16; 0.08) |  | -0.02 (-0.14; 0.09) |
| HbA1c, (%) |  | 0.02 (-0.18; 0.24) |  | -0.04 (-0.09; 0.02) |  | -0.02 (-0.14; 0.10) |  | -0.04 (-0.13; 0.06) |  | -0.05 (-0.14; 0.07) |
| LDL cholesterol, mmol/L |  | **-0.33 (-0.83; -0.30)**** |  | **-0.32 (-0.36; -0.24)**** |  | **-0.28 (-0.46; -0.20)**** |  | **-0.34 (-0.44; -0.23)**** |  | **-0.31 (-0.35; -0.15)**** |
| SES score |  | 0.05 (-0.21; 0.41) |  | <0.01 (-0.07; 0.06) |  | -0.08 (-0.24; 0.05) |  | 0.07 (-0.06; 0.19) |  | <0.01 (-0.12; 0.13) |
| Proline, AU | **0.42**** | 0.10 (-0.05; 0.31) | **0.31**** | 0.03 (-0.03; 0.08) | **0.31**** | 0.04 (-0.06; 0.14) | **0.33**** | 0.10 (<0.01; 0.19) | **0.24**** | -0.07 (-0.15; 0.04) |
| Age, years |  | **-0.16 (-0.39; <0.01)*** |  | **-0.08 (-0.14; -0.02)*** |  | -0.09 (-0.20; 0.02) |  | **-0.12 (-0.22; <0.01)*** |  | -0.02 (-0.13; 0.09) |
| Sex, female/male |  | **0.36 (0.22; 0.64)**** |  | **0.25 (0.18; 0.31)**** |  | **0.27 (0.16; 0.41)**** |  | **0.23 (0.10; 0.33)**** |  | **0.22 (0.08; 0.32)**** |
| Ethnicity, Black/White |  | **-0.20 (-0.48; -0.05)*** |  | **-0.19 (-0.26; -0.11)**** |  | **-0.16 (-0.29; -0.03)*** |  | **-0.25 (-0.36; -0.11)**** |  | **-0.15 (-0.28; -0.01)*** |
| Protein intake, g |  | -0.01 (-0.18; 0.16) |  | 0.04 (-0.02; 0.10) |  | 0.02 (-0.09; 0.14) |  | 0.10 (<0.01; 0.19) |  | -0.02 (-0.12; 0.08) |
| Waist-to-height ratio |  | 0.05 (-0.14; 0.30) |  | 0.04 (-0.02; 0.10) |  | 0.09 (-0.02; 0.30) |  | 0.03 (-0.09; 0.17) |  | -0.05 (-0.14; 0.06) |
| Physical act, kCal/kg/day |  | 0.02 (-0.20; 0.26) |  | 0.03 (-0.02; 0.09) |  | 0.02 (-0.10; 0.15) |  | 0.06 (-0.04; 0.14) |  | 0.01 (-0.08; 0.10) |
| Cotinine, ng/ml |  |  |  | **-0.08 (-0.13; -0.02)*** |  | -0.03 (-0.20; 0.10) |  | -0.03 (-0.13; 0.06) |  | **-0.15 (-0.21; -0.03)*** |
| GGT, U/L |  | **-0.35 (-0.71; -0.25)**** |  | **-0.25 (-0.31; -0.18)**** |  | **-0.27 (-0.44; -0.18)**** |  | **-0.19 (-0.33; -0.09)**** |  | **-0.23 (-0.32; -0.10)**** |
| 24h Systolic BP, mmHg |  | -0.10 (-0.40; 0.11) |  | -0.01 (-0.08; 0.05) |  | 0.01 (-0.12; 0.15) |  | -0.03 (-0.15; 0.09) |  | -0.02 (-0.13; 0.10) |
| HbA1c, (%) |  | 0.01 (-0.19; 0.22) |  | -0.04 (-0.10; 0.02) |  | -0.02 (-0.15; 0.10) |  | -0.05 (-0.14; 0.05) |  | -0.05 (-0.15; 0.06) |
| LDL cholesterol, mmol/L |  | **-0.32 (-0.82; -0.29)**** |  | **-0.32 (-0.36; -0.24)**** |  | **-0.29 (-0.47; -0.21)**** |  | **-0.34 (-0.44; -0.23)**** |  | **-0.31 (-0.35; -0.15)**** |
| SES score |  | 0.06 (-0.19; 0.43) |  | <0.01 (-0.07; 0.07) |  | -0.07 (-0.22; 0.06) |  | 0.07 (-0.06; 0.19) |  | 0.02 (-0.11; 0.14) |
| Cystine, AU | **0.41**** | <0.01 (-0.16; 0.17) | **0.32**** | **0.07 (0.02; 0.13)*** | **0.33**** | **0.14 (0.05; 0.27)*** | **0.33**** | **0.12 (0.03; 0.22)*** | **0.23**** | -0.03 (-0.11; 0.07) |
| Age, years |  | -0.16 (-0.39; <0.01) |  | **-0.09 (-0.15; -0.02)*** |  | -0.10 (-0.21; 0.01) |  | **-0.12 (-0.22; -0.01)*** |  | -0.02 (-0.12; 0.09) |
| Sex, female/male |  | **0.34 (0.20; 0.62)**** |  | **0.25 (0.18; 0.31)**** |  | **0.26 (0.14; 0.39)**** |  | **0.22 (0.10; 0.32)**** |  | **0.23 (0.08; 0.33)**** |
| Ethnicity, Black/White |  | **-0.18 (-0.46; -0.03)*** |  | **-0.18 (-0.25; -0.11)**** |  | **-0.16 (-0.29; -0.03)*** |  | **-0.24 (-0.35; -0.10)**** |  | **-0.16 (-0.29; -0.01)*** |
| Protein intake, g |  | -0.01 (-0.19; 0.16) |  | 0.04 (-0.02; 0.10) |  | 0.04 (-0.07; 0.15) |  | 0.09 (-0.01; 0.19) |  | -0.01 (-0.11; 0.09) |
| Waist-to-height ratio |  | 0.04 (-0.16; 0.29) |  | 0.04 (-0.03; 0.10) |  | 0.09 (-0.02; 0.29) |  | 0.02 (-0.11; 0.15) |  | -0.05 (-0.13; 0.06) |
| Physical act, kCal/kg/day |  | 0.03 (-0.19; 0.28) |  | 0.03 (-0.02; 0.09) |  | 0.02 (-0.10; 0.14) |  | 0.06 (-0.04; 0.14) |  | 0.01 (-0.08; 0.10) |
| Cotinine, ng/ml |  |  |  | **-0.07 (-0.12; -0.02)*** |  | -0.01 (-0.17; 0.13) |  | -0.03 (-0.12; 0.07) |  | **-0.16 (-0.22; -0.03)*** |
| GGT, U/L |  | **-0.36 (-0.73; -0.27)**** |  | **-0.25 (-0.31; -0.18)**** |  | **-0.25 (-0.41; -0.16)**** |  | **-0.19 (-0.33; -0.10)**** |  | **-0.23 (-0.31; -0.10)**** |
| 24h Systolic BP, mmHg |  | -0.09 (-0.39; 0.13) |  | -0.02 (-0.08; 0.05) |  | 0.01 (-0.12; 0.15) |  | -0.03 (-0.15; 0.08) |  | -0.02 (-0.14; 0.10) |
| HbA1c, (%) |  | 0.01 (-0.19; 0.22) |  | -0.04 (-0.10; 0.02) |  | -0.02 (-0.14; 0.10) |  | -0.04 (-0.14; 0.05) |  | -0.05 (-0.15; 0.06) |
| LDL cholesterol, mmol/L |  | **-0.34 (-0.85; -0.32)**** |  | **-0.32 (-0.36; -0.24)**** |  | **-0.27 (-0.45; -0.20)**** |  | **-0.35 (-0.45; -0.24)**** |  | **-0.31 (-0.34; -0.14)**** |
| SES score |  | 0.06 (-0.20; 0.43) |  | <0.01 (-0.07; 0.07) |  | -0.07 (-0.22; 0.06) |  | 0.08 (-0.06; 0.20) |  | 0.01 (-0.11; 0.14) |

Test used: Multiple linear regressions. Data are presented as adjusted R^2^ with β coefficient and 95% confidence intervals. Estimated glomerular filtration rate (cystatin C-based), adjusted for age, sex, ethnicity, protein intake, waist-to-hight ratio, physical activity, cotinine, GGT, 24h systolic BP, HbA1c, LDL, SES score. Bold values denote P≤0.05; *P≤0.05; **P≤0.001. Cardiovascular disease risk group criteria: Obese - ≥0.55 waist-to-height ratio; Physically inactive - <600 METs for moderate and/or vigorous intensity physical activity; Smoking - ≥11 ng/mL cotinine & self-reported smoking; Excessive alcohol intake - ≥49 U/L GGT & self-reported drinking; Masked hypertensive – normal clinic BP & 24h/day/night BP classified as hypertensive; Hyperglycemic - ≥5.7% HbA1c; Dyslipidemic - >3.4 mmol/L LDL; Low socio-economic – low SES.

*Abbreviations*: AU, arbitrary units; physical act, physical activity; GGT, gamma-glutamyl transferase; BP, blood pressure; HbA1c, glycated haemoglobin; LDL, low density lipoprotein; SES, socio-economic status; GFR, estimated glomerular filtration rate; CVD, cardiovascular disease.

**Supplementary Table 2F. Multi-variable adjusted regression analysis with estimated glomerular filtration rate (cystatin C-based) as the dependent variable, with the metabolomics data in control, cardiovascular disease risk group and cardiovascular disease risk clusters**

|  | **eGFR (cystatin-C), ml/min/1.73m^2^** | | | | | | | | | |
| --- | --- | --- | --- | --- | --- | --- | --- | --- | --- | --- |
|  | **Control group**  **(N=166)** | | **CVD risk group**  **(N=1036)** | | **1 CVD risk factor**  **(N=344)** | | **2 CVD risk factors (N=360)** | | **3+ CVD risk factors (N=332)** | |
| ***Metabolomic data*** | **Adj R^2^** | **β (95%Cl)** | **Adj R^2^** | **β (95%Cl)** | **Adj R^2^** | **β (95%Cl)** | **Adj R^2^** | **β (95%Cl)** | **Adj R^2^** | **β (95%Cl)** |
| Valine, AU | **0.43**** | **0.14 (<0.01; 0.36)*** | **0.33**** | **0.13 (0.07; 0.18)**** | **0.33**** | **0.12 (0.02; 0.23)*** | **0.36**** | **0.20 (0.10; 0.28)**** | **0.23**** | 0.06 (-0.05; 0.15) |
| Age, years |  | **-0.17 (-0.40; -0.02)*** |  | **-0.09 (-0.14; -0.02)*** |  | -0.08 (-0.20; 0.03) |  | **-0.12 (-0.22; -0.01)*** |  | -0.02 (-0.13; 0.09) |
| Sex, female/male |  | **0.36 (0.22; 0.64)**** |  | **0.25 (0.18; 0.31)**** |  | **0.27 (0.15; 0.40)**** |  | **0.24 (0.12; 0.34)**** |  | **0.23 (0.09; 0.34)**** |
| Ethnicity, Black/White |  | **-0.20 (-0.48; -0.05)*** |  | **-0.20 (-0.26; -0.12)**** |  | **-0.16 (-0.29; -0.03)*** |  | **-0.27 (-0.38; -0.14)**** |  | **-0.16 (-0.29; -0.01)*** |
| Protein intake, g |  | -0.04 (-0.22; 0.12) |  | 0.05 (-0.01; 0.10) |  | 0.03 (-0.08; 0.14) |  | 0.08 (-0.01; 0.18) |  | <0.01 (-0.10; 0.10) |
| Waist-to-height ratio |  | 0.04 (-0.15; 0.28) |  | 0.04 (-0.03; 0.10) |  | 0.08 (-0.03; 0.28) |  | 0.03 (-0.08; 0.17) |  | -0.04 (-0.13; 0.07) |
| Physical act, kCal/kg/day |  | 0.01 (-0.21; 0.25) |  | 0.03 (-0.02; 0.08) |  | 0.01 (-0.11; 0.14) |  | 0.05 (-0.04; 0.13) |  | 0.01 (-0.08; 0.10) |
| Cotinine, ng/ml |  |  |  | **-0.07 (-0.12; -0.01)*** |  | -0.02 (-0.18; 0.11) |  | -0.02 (-0.11; 0.08) |  | **-0.15 (-0.21; -0.02)*** |
| GGT, U/L |  | **-0.35 (-0.70; -0.25)**** |  | **-0.25 (-0.31; -0.18)**** |  | **-0.26 (-0.42; -0.16)**** |  | **-0.20 (-0.34; -0.11)**** |  | **-0.23 (-0.31; -0.10)**** |
| 24h Systolic BP, mmHg |  | -0.11 (-0.41; 0.10) |  | -0.02 (-0.09; 0.05) |  | 0.02 (-0.11; 0.16) |  | -0.04 (-0.16; 0.07) |  | -0.03 (-0.14; 0.09) |
| HbA1c, (%) |  | 0.02 (-0.18; 0.23) |  | -0.04 (-0.10; 0.02) |  | -0.02 (-0.15; 0.10) |  | -0.04 (-0.13; 0.05) |  | -0.05 (-0.14; 0.06) |
| LDL cholesterol, mmol/L |  | **-0.32 (-0.80; -0.29)**** |  | **-0.31 (-0.35; -0.23)**** |  | **-0.27 (-0.45; -0.19)**** |  | **-0.33 (-0.43; -0.22)**** |  | **-0.31 (-0.34; -0.14)**** |
| SES score |  | 0.06 (-0.19; 0.43) |  | <0.01 (-0.07; 0.06) |  | -0.08 (-0.24; 0.05) |  | 0.08 (-0.05; 0.20) |  | <0.01 (-0.12; 0.13) |
| Methionine, AU | **0.41**** | 0.06 (-0.11; 0.28) | **0.32**** | **0.09 (0.03; 0.14)*** | **0.32**** | **0.11 (0.01; 0.22)*** | **0.34**** | **0.16 (0.06; 0.24)**** | **0.23**** | -0.02 (-0.11; 0.08) |
| Age, years |  | **-0.17 (-0.40; -0.01)*** |  | **-0.09 (-0.14; -0.02)*** |  | -0.09 (-0.20; 0.03) |  | **-0.12 (-0.22; -0.01)*** |  | -0.02 (-0.13; 0.09) |
| Sex, female/male |  | **0.35 (0.21; 0.63)**** |  | **0.25 (0.18; 0.31)**** |  | **0.27 (0.16; 0.41)**** |  | **0.23 (0.11; 0.33)**** |  | **0.23 (0.09; 0.33)**** |
| Ethnicity, Black/White |  | **-0.18 (-0.46; -0.03)*** |  | **-0.17 (-0.24; -0.10)**** |  | **-0.14 (-0.28; -0.01)*** |  | **-0.22 (-0.33; -0.09)**** |  | **-0.16 (-0.29; -0.01)*** |
| Protein intake, g |  | -0.02 (-0.19; 0.15) |  | 0.05 (-0.01; 0.10) |  | 0.03 (-0.08; 0.15) |  | 0.10 (<0.01; 0.19) |  | -0.01 (-0.11; 0.09) |
| Waist-to-height ratio |  | 0.05 (-0.15; 0.29) |  | 0.05 (-0.02; 0.11) |  | 0.09 (-0.02; 0.30) |  | 0.05 (-0.07; 0.18) |  | -0.05 (-0.13; 0.06) |
| Physical act, kCal/kg/day |  | 0.02 (-0.19; 0.27) |  | 0.03 (-0.02; 0.08) |  | 0.02 (-0.10; 0.14) |  | 0.05 (-0.04; 0.13) |  | 0.01 (-0.08; 0.10) |
| Cotinine, ng/ml |  |  |  | **-0.07 (-0.12; -0.01)*** |  | -0.02 (-0.18; 0.12) |  | -0.02 (-0.11; 0.07) |  | **-0.16 (-0.22; -0.03)*** |
| GGT, U/L |  | **-0.36 (-0.72; -0.26)**** |  | **-0.25 (-0.31; -0.18)**** |  | **-0.26 (-0.42; -0.16)**** |  | **-0.20 (-0.33; -0.10)**** |  | **-0.23 (-0.31; -0.10)**** |
| 24h Systolic BP, mmHg |  | -0.10 (-0.40; 0.12) |  | -0.02 (-0.09; 0.05) |  | 0.01 (-0.13; 0.15) |  | -0.04 (-0.16; 0.07) |  | -0.02 (-0.14; 0.10) |
| HbA1c, (%) |  | 0.01 (-0.19; 0.23) |  | -0.04 (-0.10; 0.02) |  | -0.03 (-0.15; 0.09) |  | -0.05 (-0.14; 0.05) |  | -0.05 (-0.15; 0.06) |
| LDL cholesterol, mmol/L |  | **-0.33 (-0.83; -0.29)**** |  | **-0.31 (-0.35; -0.23)**** |  | **-0.28 (-0.46; -0.20)**** |  | **-0.33 (-0.43; -0.22)**** |  | **-0.31 (-0.34; -0.14)**** |
| SES score |  | 0.06 (-0.19; 0.44) |  | <0.01 (-0.07; 0.07) |  | -0.08 (-0.24; 0.05) |  | 0.07 (-0.06; 0.19) |  | 0.01 (-0.11; 0.14) |
| Tyrosine, AU | **0.42**** | 0.14 (<0.01; 0.33) | **0.32**** | **0.09 (0.04; 0.14)**** | **0.32**** | 0.09 (-0.01; 0.19) | **0.34**** | **0.14 (0.05; 0.23)*** | **0.23**** | 0.05 (-0.05; 0.14) |
| Age, years |  | **-0.17 (-0.39; -0.01)*** |  | **-0.08 (-0.14; -0.02)*** |  | -0.09 (-0.20; 0.02) |  | **-0.11 (-0.21; <0.01)*** |  | -0.02 (-0.13; 0.09) |
| Sex, female/male |  | **0.36 (0.22; 0.64)**** |  | **0.25 (0.18; 0.31)**** |  | **0.27 (0.15; 0.40)**** |  | **0.23 (0.11; 0.33)**** |  | **0.23 (0.09; 0.34)**** |
| Ethnicity, Black/White |  | **-0.21 (-0.49; -0.06)*** |  | **-0.19 (-0.26; -0.11)**** |  | **-0.16 (-0.29; -0.03)*** |  | **-0.25 (-0.36; -0.12)**** |  | **-0.16 (-0.29; -0.01)*** |
| Protein intake, g |  | -0.03 (-0.20; 0.14) |  | 0.05 (-0.01; 0.10) |  | 0.03 (-0.08; 0.14) |  | **0.10 (<0.01; 0.19)*** |  | -0.01 (-0.11; 0.10) |
| Waist-to-height ratio |  | 0.05 (-0.15; 0.29) |  | 0.04 (-0.03; 0.10) |  | 0.08 (-0.03; 0.28) |  | 0.03 (-0.09; 0.16) |  | -0.05 (-0.13; 0.06) |
| Physical act, kCal/kg/day |  | 0.02 (-0.20; 0.26) |  | 0.03 (-0.02; 0.08) |  | 0.02 (-0.10; 0.14) |  | 0.05 (-0.04; 0.13) |  | 0.01 (-0.08; 0.10) |
| Cotinine, ng/ml |  |  |  | **-0.07 (-0.12; -0.01)*** |  | -0.03 (-0.19; 0.11) |  | -0.02 (-0.11; 0.07) |  | **-0.15 (-0.21; -0.03)*** |
| GGT, U/L |  | **-0.37 (-0.73; -0.28)**** |  | **-0.25 (-0.31; -0.18)**** |  | **-0.27 (-0.43; -0.17)**** |  | **-0.20 (-0.34; -0.11)**** |  | **-0.23 (-0.31; -0.10)**** |
| 24h Systolic BP, mmHg |  | -0.11 (-0.42; 0.09) |  | -0.02 (-0.09; 0.05) |  | 0.02 (-0.12; 0.15) |  | -0.04 (-0.16; 0.07) |  | -0.02 (-0.14; 0.10) |
| HbA1c, (%) |  | 0.02 (-0.18; 0.23) |  | -0.04 (-0.10; 0.02) |  | -0.02 (-0.15; 0.09) |  | -0.04 (-0.14; 0.05) |  | -0.05 (-0.14; 0.06) |
| LDL cholesterol, mmol/L |  | **-0.31 (-0.79; -0.26)**** |  | **-0.31 (-0.35; -0.23)**** |  | **-0.28 (-0.46; -0.20)**** |  | **-0.33 (-0.43; -0.22)**** |  | **-0.30 (-0.34; -0.14)**** |
| SES score |  | 0.06 (-0.19; 0.43) |  | <0.01 (-0.07; 0.07) |  | -0.07 (-0.23; 0.06) |  | 0.07 (-0.06; 0.19) |  | 0.01 (-0.12; 0.13) |
| Pyroglutamic acid, AU | **0.41**** | 0.09 (-0.06; 0.29) | **0.32**** | **0.07 (0.01; 0.12)*** | **0.32**** | 0.08 (-0.02; 0.19) | **0.34**** | **0.13 (0.03; 0.22)*** | **0.23**** | -0.01 (-0.10; 0.09) |
| Age, years |  | **-0.17 (-0.39; -0.01)*** |  | **-0.09 (-0.15; -0.02)*** |  | -0.09 (-0.21; 0.02) |  | **-0.13 (-0.23; -0.02)*** |  | -0.02 (-0.13; 0.09) |
| Sex, female/male |  | **0.35 (0.21; 0.63)**** |  | **0.25 (0.18; 0.31)**** |  | **0.27 (0.15; 0.40)**** |  | **0.23 (0.10; 0.33)**** |  | **0.23 (0.09; 0.33)**** |
| Ethnicity, Black/White |  | **-0.18 (-0.46; -0.03)*** |  | **-0.18 (-0.25; -0.10)**** |  | **-0.15 (-0.29; -0.02)*** |  | **-0.24 (-0.35; -0.11)**** |  | **-0.16 (-0.29; -0.01)*** |
| Protein intake, g |  | -0.02 (-0.19; 0.15) |  | 0.04 (-0.01; 0.10) |  | 0.03 (-0.08; 0.14) |  | 0.10 (<0.01; 0.19) |  | -0.01 (-0.11; 0.09) |
| Waist-to-height ratio |  | 0.04 (-0.15; 0.28) |  | 0.05 (-0.02; 0.11) |  | 0.09 (-0.02; 0.30) |  | 0.04 (-0.08; 0.18) |  | -0.05 (-0.13; 0.06) |
| Physical act, kCal/kg/day |  | 0.02 (-0.20; 0.27) |  | 0.03 (-0.02; 0.08) |  | 0.02 (-0.10; 0.14) |  | 0.05 (-0.04; 0.13) |  | 0.01 (-0.08; 0.10) |
| Cotinine, ng/ml |  |  |  | **-0.08 (-0.12; -0.02)*** |  | -0.03 (-0.19; 0.11) |  | -0.03 (-0.12; 0.07) |  | **-0.16 (-0.22; -0.03)*** |
| GGT, U/L |  | **-0.36 (-0.72; -0.26)**** |  | **-0.25 (-0.31; -0.18)**** |  | **-0.26 (-0.43; -0.17)**** |  | **-0.20 (-0.33; -0.10)**** |  | **-0.23 (-0.31; -0.10)**** |
| 24h Systolic BP, mmHg |  | -0.10 (-0.40; 0.11) |  | -0.02 (-0.09; 0.05) |  | 0.02 (-0.12; 0.15) |  | -0.04 (-0.15; 0.08) |  | -0.02 (-0.14; 0.10) |
| HbA1c, (%) |  | 0.01 (-0.19; 0.23) |  | -0.04 (-0.10; 0.02) |  | -0.02 (-0.15; 0.10) |  | -0.05 (-0.14; 0.05) |  | -0.05 (-0.15; 0.06) |
| LDL cholesterol, mmol/L |  | **-0.32 (-0.82; -0.29)**** |  | **-0.31 (-0.35; -0.23)**** |  | **-0.28 (-0.46; -0.20)**** |  | **-0.33 (-0.43; -0.22)**** |  | **-0.31 (-0.34; -0.14)**** |
| SES score |  | 0.06 (-0.20; 0.43) |  | <0.01 (-0.07; 0.07) |  | -0.07 (-0.23; 0.06) |  | 0.07 (-0.06; 0.19) |  | 0.01 (-0.11; 0.13) |

Test used: Multiple linear regressions. Data are presented as adjusted R^2^ with β coefficient and 95% confidence intervals. Estimated glomerular filtration rate (cystatin C-based), adjusted for age, sex, ethnicity, protein intake, waist-to-hight ratio, physical activity, cotinine, GGT, 24h systolic BP, HbA1c, LDL, SES score. Bold values denote P≤0.05; *P≤0.05; **P≤0.001. Cardiovascular disease risk group criteria: Obese - ≥0.55 waist-to-height ratio; Physically inactive - <600 METs for moderate and/or vigorous intensity physical activity; Smoking - ≥11 ng/mL cotinine & self-reported smoking; Excessive alcohol intake - ≥49 U/L GGT & self-reported drinking; Masked hypertensive – normal clinic BP & 24h/day/night BP classified as hypertensive; Hyperglycemic - ≥5.7% HbA1c; Dyslipidemic - >3.4 mmol/L LDL; Low socio-economic – low SES.

*Abbreviations*: AU, arbitrary units; physical act, physical activity; GGT, gamma-glutamyl transferase; BP, blood pressure; HbA1c, glycated haemoglobin; LDL, low density lipoprotein; SES, socio-economic status; GFR, estimated glomerular filtration rate; CVD, cardiovascular disease.

**Supplementary Table 2G. Multi-variable adjusted regression analysis with estimated glomerular filtration rate (cystatin C-based) as the dependent variable, with the metabolomics data in control, cardiovascular disease risk group and cardiovascular disease risk clusters**

|  | **eGFR (cystatin-C), ml/min/1.73m^2^** | | | | | | | | | |
| --- | --- | --- | --- | --- | --- | --- | --- | --- | --- | --- |
|  | **Control group**  **(N=166)** | | **CVD risk group**  **(N=1036)** | | **1 CVD risk factor**  **(N=344)** | | **2 CVD risk factors (N=360)** | | **3+ CVD risk factors (N=332)** | |
| ***Metabolomic data*** | **Adj R^2^** | **β (95%Cl)** | **Adj R^2^** | **β (95%Cl)** | **Adj R^2^** | **β (95%Cl)** | **Adj R^2^** | **β (95%Cl)** | **Adj R^2^** | **β (95%Cl)** |
| Leucine/Isoleucine, AU | **0.42**** | 0.12 (-0.03; 0.33) | **0.32**** | **0.11 (0.05; 0.16)**** | **0.32**** | 0.09 (-0.01; 0.19) | **0.35**** | **0.19 (0.09; 0.27)**** | **0.23**** | 0.05 (-0.06; 0.14) |
| Age, years |  | **-0.17 (-0.39; -0.01)*** |  | **-0.08 (-0.14; -0.02)*** |  | -0.08 (-0.20; 0.03) |  | **-0.12 (-0.22; -0.01)*** |  | -0.02 (-0.13; 0.09) |
| Sex, female/male |  | **0.35 (0.21; 0.62)**** |  | **0.25 (0.18; 0.31)**** |  | **0.27 (0.15; 0.40)**** |  | **0.23 (0.11; 0.33)**** |  | **0.23 (0.09; 0.34)**** |
| Ethnicity, Black/White |  | **-0.19 (-0.47; -0.04)*** |  | **-0.19 (-0.26; -0.11)**** |  | **-0.16 (-0.29; -0.03)*** |  | **-0.26 (-0.37; -0.13)**** |  | **-0.16 (-0.29; -0.01)*** |
| Protein intake, g |  | -0.03 (-0.21; 0.13) |  | 0.05 (-0.01; 0.10) |  | 0.03 (-0.08; 0.14) |  | 0.09 (-0.01; 0.18) |  | <0.01 (-0.10; 0.10) |
| Waist-to-height ratio |  | 0.04 (-0.15; 0.28) |  | 0.04 (-0.03; 0.10) |  | 0.09 (-0.03; 0.29) |  | 0.03 (-0.09; 0.16) |  | -0.05 (-0.13; 0.07) |
| Physical act, kCal/kg/day |  | 0.01 (-0.21; 0.25) |  | 0.03 (-0.02; 0.08) |  | 0.02 (-0.10; 0.14) |  | 0.04 (-0.05; 0.12) |  | 0.01 (-0.08; 0.10) |
| Cotinine, ng/ml |  |  |  | **-0.07 (-0.12; -0.01)*** |  | -0.03 (-0.19; 0.10) |  | -0.02 (-0.11; 0.08) |  | **-0.15 (-0.21; -0.03)*** |
| GGT, U/L |  | **-0.35 (-0.71; -0.26)**** |  | **-0.25 (-0.31; -0.18)**** |  | **-0.27 (-0.43; -0.17)**** |  | **-0.20 (-0.33; -0.11)**** |  | **-0.22 (-0.31; -0.10)**** |
| 24h Systolic BP, mmHg |  | -0.10 (-0.41; 0.11) |  | -0.02 (-0.09; 0.05) |  | 0.02 (-0.12; 0.15) |  | -0.05 (-0.16; 0.07) |  | -0.03 (-0.14; 0.09) |
| HbA1c, (%) |  | 0.01 (-0.19; 0.23) |  | -0.04 (-0.10; 0.02) |  | -0.02 (-0.15; 0.10) |  | -0.05 (-0.14; 0.04) |  | -0.05 (-0.14; 0.06) |
| LDL cholesterol, mmol/L |  | **-0.32 (-0.81; -0.29)**** |  | **-0.31 (-0.35; -0.23)**** |  | **-0.28 (-0.46; -0.20)**** |  | **-0.33 (-0.43; -0.22)**** |  | **-0.31 (-0.34; -0.14)**** |
| SES score |  | 0.06 (-0.19; 0.43) |  | <0.01 (-0.07; 0.07) |  | -0.08 (-0.24; 0.05) |  | 0.07 (-0.05; 0.19) |  | 0.01 (-0.12; 0.13) |
| Phenylalanine, AU | **0.43**** | **0.15 (0.01; 0.35)*** | **0.32**** | **0.11 (0.05; 0.16)**** | **0.33**** | **0.12 (0.02; 0.23)*** | **0.35**** | **0.16 (0.07; 0.25)**** | **0.23**** | 0.05 (-0.05; 0.14) |
| Age, years |  | **-0.16 (-0.39; -0.01)*** |  | **-0.08 (-0.14; -0.02)*** |  | -0.08 (-0.20; 0.03) |  | **-0.12 (-0.22; -0.01)*** |  | -0.02 (-0.13; 0.09) |
| Sex, female/male |  | **0.35 (0.22; 0.63)**** |  | **0.25 (0.18; 0.31)**** |  | **0.27 (0.15; 0.40)**** |  | **0.23 (0.11; 0.33)**** |  | **0.23 (0.09; 0.34)**** |
| Ethnicity, Black/White |  | **-0.20 (-0.49; -0.06)*** |  | **-0.18 (-0.25; -0.11)**** |  | **-0.15 (-0.29; -0.02)*** |  | **-0.24 (-0.35; -0.11)**** |  | **-0.16 (-0.29; -0.01)*** |
| Protein intake, g |  | -0.03 (-0.21; 0.13) |  | 0.05 (-0.01; 0.10) |  | 0.03 (-0.08; 0.14) |  | **0.10 (<0.01; 0.19)*** |  | -0.01 (-0.11; 0.10) |
| Waist-to-height ratio |  | 0.04 (-0.15; 0.28) |  | 0.04 (-0.03; 0.10) |  | 0.08 (-0.03; 0.28) |  | 0.03 (-0.09; 0.16) |  | -0.05 (-0.13; 0.06) |
| Physical act, kCal/kg/day |  | 0.02 (-0.19; 0.26) |  | 0.03 (-0.02; 0.08) |  | 0.01 (-0.10; 0.14) |  | 0.05 (-0.04; 0.13) |  | 0.01 (-0.08; 0.10) |
| Cotinine, ng/ml |  |  |  | **-0.07 (-0.12; -0.01)*** |  | -0.02 (-0.18; 0.11) |  | -0.02 (-0.11; 0.08) |  | **-0.15 (-0.21; -0.03)*** |
| GGT, U/L |  | **-0.36 (-0.72; -0.27)**** |  | **-0.25 (-0.31; -0.18)**** |  | **-0.26 (-0.42; -0.17)**** |  | **-0.20 (-0.33; -0.11)**** |  | **-0.23 (-0.31; -0.10)**** |
| 24h Systolic BP, mmHg |  | -0.11 (-0.42; 0.09) |  | -0.02 (-0.09; 0.04) |  | 0.01 (-0.12; 0.15) |  | -0.04 (-0.16; 0.07) |  | -0.02 (-0.14; 0.10) |
| HbA1c, (%) |  | 0.01 (-0.18; 0.23) |  | -0.04 (-0.10; 0.02) |  | -0.03 (-0.15; 0.09) |  | -0.05 (-0.14; 0.05) |  | -0.05 (-0.15; 0.06) |
| LDL cholesterol, mmol/L |  | **-0.31 (-0.79; -0.27)**** |  | **-0.31 (-0.35; -0.23)**** |  | **-0.27 (-0.45; -0.19)**** |  | **-0.34 (-0.44; -0.22)**** |  | **-0.30 (-0.34; -0.14)**** |
| SES score |  | 0.05 (-0.20; 0.41) |  | <0.01 (-0.07; 0.06) |  | -0.08 (-0.24; 0.05) |  | 0.07 (-0.06; 0.19) |  | <0.01 (-0.12; 0.13) |
| Aspartic acid, AU | **0.41**** | 0.08 (-0.07; 0.30) | **0.32**** | **0.06 (<0.01; 0.11)*** | **0.32**** | 0.08 (-0.02; 0.19) | **0.33**** | **0.11 (0.01; 0.18)*** | **0.23**** | -0.03 (-0.12; 0.07) |
| Age, years |  | **-0.17 (-0.39; -0.01)*** |  | **-0.09 (-0.15; -0.02)*** |  | -0.09 (-0.21; 0.02) |  | **-0.13 (-0.23; -0.01)*** |  | -0.02 (-0.13; 0.09) |
| Sex, female/male |  | **0.35 (0.21; 0.62)**** |  | **0.26 (0.18; 0.32)**** |  | **0.28 (0.16; 0.41)**** |  | **0.23 (0.11; 0.34)**** |  | **0.22 (0.08; 0.33)**** |
| Ethnicity, Black/White |  | **-0.19 (-0.47; -0.04)*** |  | **-0.18 (-0.25; -0.11)**** |  | **-0.16 (-0.29; -0.03)*** |  | **-0.24 (-0.35; -0.11)**** |  | **-0.16 (-0.29; -0.01)*** |
| Protein intake, g |  | -0.02 (-0.19; 0.15) |  | 0.04 (-0.02; 0.10) |  | 0.02 (-0.09; 0.14) |  | 0.09 (-0.01; 0.19) |  | -0.01 (-0.11; 0.09) |
| Waist-to-height ratio |  | 0.05 (-0.14; 0.29) |  | 0.04 (-0.02; 0.10) |  | 0.09 (-0.02; 0.29) |  | 0.04 (-0.09; 0.17) |  | -0.05 (-0.13; 0.06) |
| Physical act, kCal/kg/day |  | 0.02 (-0.20; 0.26) |  | 0.03 (-0.02; 0.08) |  | 0.02 (-0.10; 0.14) |  | 0.05 (-0.04; 0.13) |  | 0.01 (-0.08; 0.10) |
| Cotinine, ng/ml |  |  |  | **-0.07 (-0.12; -0.02)*** |  | -0.03 (-0.19; 0.10) |  | -0.03 (-0.12; 0.07) |  | **-0.16 (-0.22; -0.03)*** |
| GGT, U/L |  | **-0.36 (-0.72; -0.26)**** |  | **-0.25 (-0.31; -0.18)**** |  | **-0.27 (-0.43; -0.17)**** |  | **-0.20 (-0.33; -0.10)**** |  | **-0.23 (-0.31; -0.10)**** |
| 24h Systolic BP, mmHg |  | -0.09 (-0.39; 0.12) |  | -0.02 (-0.09; 0.05) |  | 0.02 (-0.12; 0.15) |  | -0.04 (-0.15; 0.08) |  | -0.02 (-0.13; 0.10) |
| HbA1c, (%) |  | 0.01 (-0.19; 0.22) |  | -0.04 (-0.10; 0.02) |  | -0.02 (-0.15; 0.10) |  | -0.05 (-0.14; 0.05) |  | -0.05 (-0.15; 0.06) |
| LDL cholesterol, mmol/L |  | **-0.33 (-0.82; -0.30)**** |  | **-0.31 (-0.35; -0.23)**** |  | **-0.28 (-0.46; -0.20)**** |  | **-0.34 (-0.44; -0.22)**** |  | **-0.31 (-0.34; -0.15)**** |
| SES score |  | 0.06 (-0.20; 0.43) |  | <0.01 (-0.07; 0.07) |  | -0.07 (-0.22; 0.06) |  | 0.07 (-0.06; 0.19) |  | 0.02 (-0.11; 0.14) |
| Tryptophan, AU | **0.42**** | 0.14 (-0.01; 0.35) | **0.33**** | **0.12 (0.06; 0.17)**** | **0.32**** | **0.11 (0.02; 0.22)*** | **0.35**** | **0.16 (0.06; 0.23)**** | **0.24**** | 0.08 (-0.02; 0.17) |
| Age, years |  | **-0.16 (-0.39; -0.01)*** |  | **-0.08 (-0.14; -0.02)*** |  | -0.09 (-0.20; 0.02) |  | **-0.11 (-0.21; <0.01)*** |  | -0.02 (-0.13; 0.09) |
| Sex, female/male |  | **0.35 (0.22; 0.63)**** |  | **0.26 (0.18; 0.32)**** |  | **0.27 (0.15; 0.40)**** |  | **0.23 (0.11; 0.33)**** |  | **0.24 (0.09; 0.34)**** |
| Ethnicity, Black/White |  | **-0.21 (-0.50; -0.07)*** |  | **-0.19 (-0.26; -0.12)**** |  | **-0.16 (-0.30; -0.03)*** |  | -0.25 (-0.36; -0.12)** |  | **-0.16 (-0.29; -0.02)*** |
| Protein intake, g |  | -0.02 (-0.20; 0.14) |  | 0.05 (-0.01; 0.10) |  | 0.03 (-0.08; 0.14) |  | 0.10 (<0.01; 0.19) |  | -0.01 (-0.11; 0.10) |
| Waist-to-height ratio |  | 0.05 (-0.15; 0.29) |  | 0.04 (-0.03; 0.10) |  | 0.08 (-0.03; 0.28) |  | 0.03 (-0.09; 0.16) |  | -0.05 (-0.13; 0.06) |
| Physical act, kCal/kg/day |  | 0.02 (-0.21; 0.26) |  | 0.03 (-0.02; 0.08) |  | 0.02 (-0.10; 0.14) |  | 0.05 (-0.05; 0.13) |  | 0.01 (-0.08; 0.10) |
| Cotinine, ng/ml |  |  |  | **-0.07 (-0.12; -0.01)*** |  | -0.02 (-0.18; 0.11) |  | -0.03 (-0.12; 0.07) |  | **-0.15 (-0.21; -0.03)*** |
| GGT, U/L |  | **-0.37 (-0.73; -0.28)**** |  | **-0.25 (-0.31; -0.18)**** |  | **-0.26 (-0.42; -0.16)**** |  | **-0.21 (-0.34; -0.12)**** |  | **-0.23 (-0.31; -0.10)**** |
| 24h Systolic BP, mmHg |  | -0.11 (-0.42; 0.09) |  | -0.02 (-0.09; 0.04) |  | 0.01 (-0.12; 0.15) |  | -0.04 (-0.15; 0.08) |  | -0.03 (-0.14; 0.09) |
| HbA1c, (%) |  | 0.02 (-0.17; 0.24) |  | -0.04 (-0.10; 0.02) |  | -0.02 (-0.15; 0.09) |  | -0.04 (-0.14; 0.05) |  | -0.05 (-0.14; 0.06) |
| LDL cholesterol, mmol/L |  | **-0.31 (-0.79; -0.27)**** |  | **-0.31 (-0.35; -0.23)**** |  | **-0.27 (-0.45; -0.19)**** |  | **-0.34 (-0.44; -0.23)**** |  | **-0.30 (-0.34; -0.14)**** |
| SES score |  | 0.05 (-0.21; 0.41) |  | -0.01 (-0.07; 0.06) |  | -0.08 (-0.24; 0.05) |  | 0.06 (-0.06; 0.18) |  | <0.01 (-0.12; 0.12) |

Test used: Multiple linear regressions. Data are presented as adjusted R^2^ with β coefficient and 95% confidence intervals. Estimated glomerular filtration rate (cystatin C-based), adjusted for age, sex, ethnicity, protein intake, waist-to-hight ratio, physical activity, cotinine, GGT, 24h systolic BP, HbA1c, LDL, SES score. Bold values denote P≤0.05; *P≤0.05; **P≤0.001. Cardiovascular disease risk group criteria: Obese - ≥0.55 waist-to-height ratio; Physically inactive - <600 METs for moderate and/or vigorous intensity physical activity; Smoking - ≥11 ng/mL cotinine & self-reported smoking; Excessive alcohol intake - ≥49 U/L GGT & self-reported drinking; Masked hypertensive – normal clinic BP & 24h/day/night BP classified as hypertensive; Hyperglycemic - ≥5.7% HbA1c; Dyslipidemic - >3.4 mmol/L LDL; Low socio-economic – low SES.

*Abbreviations*: AU, arbitrary units; physical act, physical activity; GGT, gamma-glutamyl transferase; BP, blood pressure; HbA1c, glycated haemoglobin; LDL, low density lipoprotein; SES, socio-economic status; GFR, estimated glomerular filtration rate; CVD, cardiovascular disease.

**Supplementary Table 2H. Multi-variable adjusted regression analysis with estimated glomerular filtration rate (cystatin C-based) as the dependent variable, with the metabolomics data in control, cardiovascular disease risk group and cardiovascular disease risk clusters**

|  | **eGFR (cystatin-C), ml/min/1.73m^2^** | | | | | | | | | |
| --- | --- | --- | --- | --- | --- | --- | --- | --- | --- | --- |
|  | **Control group**  **(N=166)** | | **CVD risk group**  **(N=1036)** | | **1 CVD risk factor**  **(N=344)** | | **2 CVD risk factors (N=360)** | | **3+ CVD risk factors (N=332)** | |
| ***Metabolomic data*** | **Adj R^2^** | **β (95%Cl)** | **Adj R^2^** | **β (95%Cl)** | **Adj R^2^** | **β (95%Cl)** | **Adj R^2^** | **β (95%Cl)** | **Adj R^2^** | **β (95%Cl)** |
| Glutamic acid, AU | **0.41**** | 0.06 (-0.10; 0.26) | **0.32**** | **0.08 (0.02; 0.13)*** | **0.32**** | 0.07 (-0.03; 0.18) | **0.34**** | **0.13 (0.03; 0.21)*** | **0.23**** | <0.01 (-0.09; 0.10) |
| Age, years |  | **-0.17 (-0.39; -0.01)*** |  | **-0.09 (-0.15; -0.02)*** |  | -0.09 (-0.21; 0.02) |  | **-0.13 (-0.23; -0.02)*** |  | -0.02 (-0.13; 0.09) |
| Sex, female/male |  | **0.35 (0.21; 0.63)**** |  | **0.26 (0.18; 0.32)**** |  | **0.27 (0.16; 0.41)**** |  | **0.24 (0.11; 0.34)**** |  | **0.23 (0.09; 0.34)**** |
| Ethnicity, Black/White |  | **-0.19 (-0.46; -0.03)*** |  | **-0.18 (-0.25; -0.11)**** |  | **-0.15 (-0.29; -0.02)*** |  | **-0.24 (-0.36; -0.11)**** |  | **-0.16 (-0.29; -0.01)*** |
| Protein intake, g |  | -0.01 (-0.19; 0.15) |  | 0.05 (-0.01; 0.10) |  | 0.03 (-0.08; 0.14) |  | 0.10 (<0.01; 0.19) |  | -0.01 (-0.11; 0.09) |
| Waist-to-height ratio |  | 0.05 (-0.15; 0.29) |  | 0.05 (-0.02; 0.11) |  | 0.09 (-0.02; 0.30) |  | 0.04 (-0.08; 0.17) |  | -0.05 (-0.13; 0.06) |
| Physical act, kCal/kg/day |  | 0.02 (-0.20; 0.27) |  | 0.03 (-0.02; 0.08) |  | 0.02 (-0.1; 0.15) |  | 0.05 (-0.04; 0.13) |  | 0.01 (-0.08; 0.10) |
| Cotinine, ng/ml |  |  |  | **-0.08 (-0.13; -0.02)*** |  | -0.03 (-0.19; 0.11) |  | -0.03 (-0.12; 0.06) |  | **-0.16 (-0.22; -0.03)*** |
| GGT, U/L |  | **-0.36 (-0.72; -0.26)**** |  | **-0.25 (-0.31; -0.18)**** |  | **-0.26 (-0.43; -0.17)**** |  | **-0.20 (-0.33; -0.10)**** |  | **-0.23 (-0.31; -0.10)**** |
| 24h Systolic BP, mmHg |  | -0.10 (-0.40; 0.11) |  | -0.02 (-0.09; 0.05) |  | 0.01 (-0.12; 0.15) |  | -0.04 (-0.15; 0.08) |  | -0.02 (-0.14; 0.10) |
| HbA1c, (%) |  | 0.01 (-0.19; 0.23) |  | -0.04 (-0.10; 0.02) |  | -0.02 (-0.15; 0.10) |  | -0.05 (-0.14; 0.05) |  | -0.05 (-0.15; 0.06) |
| LDL cholesterol, mmol/L |  | **-0.33 (-0.83; -0.31)**** |  | **-0.31 (-0.35; -0.23)**** |  | **-0.28 (-0.46; -0.20)**** |  | **-0.33 (-0.43; -0.22)**** |  | **-0.31 (-0.34; -0.14)**** |
| SES score |  | 0.06 (-0.19; 0.43) |  | <0.01 (-0.07; 0.07) |  | -0.07 (-0.23; 0.06) |  | 0.08 (-0.05; 0.20) |  | 0.01 (-0.11; 0.13) |
| 2-Aminoadipic acid, AU | **0.44**** | **0.18 (0.05; 0.40)*** | **0.33**** | **0.15 (0.09; 0.20)**** | **0.34**** | **0.16 (0.06; 0.26)*** | **0.36**** | **0.20 (0.09; 0.27)**** | **0.24**** | 0.10 (-0.01; 0.18) |
| Age, years |  | **-0.18 (-0.40; -0.02)*** |  | **-0.09 (-0.14; -0.02)*** |  | -0.09 (-0.20; 0.02) |  | -0.11 (-0.21; <0.01) |  | -0.03 (-0.13; 0.08) |
| Sex, female/male |  | **0.34 (0.21; 0.61)**** |  | **0.25 (0.17; 0.31)**** |  | **0.25 (0.14; 0.38)**** |  | **0.23 (0.11; 0.33)**** |  | **0.23 (0.09; 0.34)**** |
| Ethnicity, Black/White |  | **-0.22 (-0.50; -0.07)*** |  | **-0.20 (-0.26; -0.12)**** |  | **-0.16 (-0.30; -0.04)*** |  | **-0.27 (-0.38; -0.14)**** |  | **-0.16 (-0.29; -0.01)*** |
| Protein intake, g |  | -0.04 (-0.21; 0.12) |  | 0.04 (-0.02; 0.09) |  | 0.02 (-0.09; 0.14) |  | 0.08 (-0.02; 0.17) |  | -0.01 (-0.11; 0.09) |
| Waist-to-height ratio |  | 0.04 (-0.15; 0.28) |  | 0.03 (-0.03; 0.09) |  | 0.07 (-0.05; 0.26) |  | 0.02 (-0.10; 0.15) |  | -0.05 (-0.13; 0.06) |
| Physical act, kCal/kg/day |  | 0.02 (-0.19; 0.26) |  | 0.03 (-0.02; 0.08) |  | 0.01 (-0.11; 0.13) |  | 0.06 (-0.04; 0.14) |  | 0.01 (-0.08; 0.10) |
| Cotinine, ng/ml |  |  |  | **-0.07 (-0.12; -0.01)*** |  | -0.02 (-0.18; 0.12) |  | -0.02 (-0.11; 0.08) |  | **-0.15 (-0.21; -0.03)*** |
| GGT, U/L |  | **-0.35 (-0.70; -0.25)**** |  | **-0.25 (-0.31; -0.19)**** |  | **-0.26 (-0.42; -0.17)**** |  | **-0.21 (-0.35; -0.12)**** |  | **-0.23 (-0.31; -0.10)**** |
| 24h Systolic BP, mmHg |  | -0.12 (-0.43; 0.08) |  | -0.02 (-0.09; 0.04) |  | 0.01 (-0.12; 0.15) |  | -0.04 (-0.15; 0.08) |  | -0.03 (-0.14; 0.09) |
| HbA1c, (%) |  | 0.01 (-0.18; 0.22) |  | -0.04 (-0.10; 0.02) |  | -0.03 (-0.16; 0.09) |  | -0.04 (-0.13; 0.06) |  | -0.04 (-0.14; 0.07) |
| LDL cholesterol, mmol/L |  | **-0.32 (-0.80; -0.28)**** |  | **-0.31 (-0.35; -0.23)**** |  | **-0.27 (-0.45; -0.19)**** |  | **-0.34 (-0.44; -0.23)**** |  | **-0.30 (-0.33; -0.14)**** |
| SES score |  | 0.05 (-0.21; 0.40) |  | -0.02 (-0.09; 0.05) |  | -0.09 (-0.25; 0.03) |  | 0.05 (-0.08; 0.17) |  | -0.01 (-0.14; 0.11) |
| Free carnitine, AU | **0.41**** | 0.07 (-0.1; 0.28) | **0.33**** | **0.15 (0.09; 0.20)**** | **0.34**** | **0.16 (0.06; 0.27)**** | **0.34**** | **0.14 (0.04; 0.24)*** | **0.25**** | **0.14 (0.03; 0.22)*** |
| Age, years |  | **-0.17 (-0.40; -0.01)*** |  | **-0.09 (-0.15; -0.03)*** |  | -0.10 (-0.21; 0.01) |  | **-0.13 (-0.23; -0.02)*** |  | -0.03 (-0.13; 0.08) |
| Sex, female/male |  | **0.33 (0.19; 0.61)**** |  | **0.22 (0.15; 0.28)**** |  | **0.23 (0.11; 0.36)**** |  | **0.18 (0.06; 0.29)*** |  | **0.22 (0.08; 0.32)**** |
| Ethnicity, Black/White |  | **-0.20 (-0.50; -0.05)*** |  | **-0.21 (-0.28; -0.14)**** |  | **-0.19 (-0.33; -0.06)*** |  | **-0.26 (-0.38; -0.13)**** |  | **-0.17 (-0.30; -0.03)*** |
| Protein intake, g |  | -0.02 (-0.20; 0.14) |  | 0.04 (-0.02; 0.10) |  | 0.03 (-0.08; 0.14) |  | 0.09 (-0.01; 0.18) |  | <0.01 (-0.10; 0.10) |
| Waist-to-height ratio |  | 0.03 (-0.19; 0.26) |  | 0.05 (-0.02; 0.11) |  | 0.08 (-0.03; 0.28) |  | 0.02 (-0.10; 0.16) |  | -0.03 (-0.12; 0.08) |
| Physical act, kCal/kg/day |  | 0.03 (-0.18; 0.29) |  | 0.03 (-0.02; 0.08) |  | 0.02 (-0.10; 0.15) |  | 0.05 (-0.04; 0.13) |  | <0.01 (-0.08; 0.09) |
| Cotinine, ng/ml |  |  |  | **-0.06 (-0.11; -0.01)*** |  | -0.03 (-0.19; 0.11) |  | -0.02 (-0.11; 0.08) |  | **-0.15 (-0.21; -0.02)*** |
| GGT, U/L |  | **-0.36 (-0.72; -0.26)**** |  | **-0.26 (-0.32; -0.19)**** |  | **-0.27 (-0.43; -0.17)**** |  | **-0.21 (-0.34; -0.12)**** |  | **-0.24 (-0.32; -0.11)**** |
| 24h Systolic BP, mmHg |  | -0.09 (-0.39; 0.12) |  | -0.02 (-0.08; 0.05) |  | 0.01 (-0.13; 0.14) |  | -0.01 (-0.13; 0.10) |  | -0.03 (-0.14; 0.09) |
| HbA1c, (%) |  | 0.01 (-0.19; 0.22) |  | -0.04 (-0.10; 0.02) |  | -0.03 (-0.15; 0.09) |  | -0.05 (-0.15; 0.04) |  | -0.04 (-0.14; 0.07) |
| LDL cholesterol, mmol/L |  | **-0.33 (-0.83; -0.31)**** |  | **-0.31 (-0.35; -0.24)**** |  | **-0.28 (-0.46; -0.21)**** |  | **-0.34 (-0.44; -0.23)**** |  | **-0.32 (-0.35; -0.15)**** |
| SES score |  | 0.07 (-0.19; 0.44) |  | -0.01 (-0.08; 0.06) |  | -0.07 (-0.23; 0.06) |  | 0.05 (-0.08; 0.17) |  | <0.01 (-0.12; 0.12) |
| Acetylcarnitine, AU | **0.41**** | 0.07 (-0.10; 0.28) | **0.33**** | **0.15 (0.09; 0.20)**** | **0.33**** | **0.15 (0.05; 0.25)*** | **0.33**** | **0.13 (0.03; 0.21)*** | **0.26**** | **0.17 (0.05; 0.24)*** |
| Age, years |  | **-0.17 (-0.40; -0.01)*** |  | **-0.09 (-0.15; -0.03)*** |  | -0.10 (-0.21; 0.01) |  | **-0.12 (-0.22; -0.01)*** |  | -0.04 (-0.14; 0.07) |
| Sex, female/male |  | **0.34 (0.20; 0.62)**** |  | **0.23 (0.16; 0.29)**** |  | **0.25 (0.14; 0.38)**** |  | **0.20 (0.08; 0.30)**** |  | **0.21 (0.08; 0.32)**** |
| Ethnicity, Black/White |  | **-0.20 (-0.50; -0.05)*** |  | **-0.21 (-0.28; -0.14)**** |  | **-0.19 (-0.33; -0.06)*** |  | **-0.26 (-0.37; -0.13)**** |  | **-0.17 (-0.30; -0.03)*** |
| Protein intake, g |  | -0.02 (-0.20; 0.15) |  | 0.04 (-0.02; 0.10) |  | 0.03 (-0.08; 0.15) |  | 0.09 (-0.01; 0.18) |  | -0.01 (-0.10; 0.09) |
| Waist-to-height ratio |  | 0.02 (-0.19; 0.26) |  | 0.05 (-0.02; 0.11) |  | 0.09 (-0.03; 0.28) |  | 0.03 (-0.10; 0.16) |  | -0.03 (-0.12; 0.08) |
| Physical act, kCal/kg/day |  | 0.03 (-0.18; 0.29) |  | 0.03 (-0.03; 0.08) |  | 0.02 (-0.10; 0.14) |  | 0.05 (-0.04; 0.13) |  | <0.01 (-0.09; 0.09) |
| Cotinine, ng/ml |  |  |  | **-0.07 (-0.11; -0.01)*** |  | -0.03 (-0.20; 0.10) |  | -0.02 (-0.12; 0.07) |  | **-0.14 (-0.20; -0.02)*** |
| GGT, U/L |  | **-0.36 (-0.72; -0.26)**** |  | **-0.26 (-0.32; -0.19)**** |  | **-0.27 (-0.43; -0.17)**** |  | **-0.21 (-0.35; -0.12)**** |  | **-0.24 (-0.32; -0.11)**** |
| 24h Systolic BP, mmHg |  | -0.10 (-0.40; 0.12) |  | -0.01 (-0.08; 0.05) |  | <0.01 (-0.13; 0.14) |  | -0.01 (-0.13; 0.10) |  | -0.02 (-0.13; 0.10) |
| HbA1c, (%) |  | 0.01 (-0.19; 0.22) |  | -0.04 (-0.10; 0.02) |  | -0.02 (-0.14; 0.10) |  | -0.05 (-0.15; 0.04) |  | -0.04 (-0.14; 0.07) |
| LDL cholesterol, mmol/L |  | **-0.33 (-0.83; -0.31)**** |  | **-0.32 (-0.36; -0.24)**** |  | **-0.29 (-0.47; -0.22)**** |  | **-0.35 (-0.45; -0.23)**** |  | **-0.32 (-0.35; -0.16)**** |
| SES score |  | 0.07 (-0.19; 0.44) |  | -0.02 (-0.08; 0.05) |  | -0.07 (-0.23; 0.06) |  | 0.04 (-0.09; 0.16) |  | -0.01 (-0.13; 0.11) |

Test used: Multiple linear regressions. Data are presented as adjusted R^2^ with β coefficient and 95% confidence intervals. Estimated glomerular filtration rate (cystatin C-based), adjusted for age, sex, ethnicity, protein intake, waist-to-hight ratio, physical activity, cotinine, GGT, 24h systolic BP, HbA1c, LDL, SES score. Bold values denote P≤0.05; *P≤0.05; **P≤0.001. Cardiovascular disease risk group criteria: Obese - ≥0.55 waist-to-height ratio; Physically inactive - <600 METs for moderate and/or vigorous intensity physical activity; Smoking - ≥11 ng/mL cotinine & self-reported smoking; Excessive alcohol intake - ≥49 U/L GGT & self-reported drinking; Masked hypertensive – normal clinic BP & 24h/day/night BP classified as hypertensive; Hyperglycemic - ≥5.7% HbA1c; Dyslipidemic - >3.4 mmol/L LDL; Low socio-economic – low SES.

*Abbreviations*: AU, arbitrary units; physical act, physical activity; GGT, gamma-glutamyl transferase; BP, blood pressure; HbA1c, glycated haemoglobin; LDL, low density lipoprotein; SES, socio-economic status; GFR, estimated glomerular filtration rate; CVD, cardiovascular disease.

**Supplementary Table 2I. Multi-variable adjusted regression analysis with estimated glomerular filtration rate (cystatin C-based) as the dependent variable, with the metabolomics data in control, cardiovascular disease risk group and cardiovascular disease risk clusters**

|  | **eGFR (cystatin-C), ml/min/1.73m^2^** | | | | | | | | | |
| --- | --- | --- | --- | --- | --- | --- | --- | --- | --- | --- |
|  | **Control group**  **(N=166)** | | **CVD risk group**  **(N=1036)** | | **1 CVD risk factor**  **(N=344)** | | **2 CVD risk factors (N=360)** | | **3+ CVD risk factors (N=332)** | |
| ***Metabolomic data*** | **Adj R^2^** | **β (95%Cl)** | **Adj R^2^** | **β (95%Cl)** | **Adj R^2^** | **β (95%Cl)** | **Adj R^2^** | **β (95%Cl)** | **Adj R^2^** | **β (95%Cl)** |
| Propionylcarnitine, AU | **0.41**** | 0.10 (-0.07; 0.30) | **0.33**** | **0.14 (0.08; 0.20)**** | **0.33**** | **0.15 (0.05; 0.26)*** | **0.33**** | **0.12 (0.01; 0.21)*** | **0.25**** | **0.15 (0.03; 0.23)*** |
| Age, years |  | **-0.17 (-0.40; <0.01)*** |  | **-0.09 (-0.15; -0.02)*** |  | -0.09 (-0.21; 0.03) |  | **-0.12 (-0.23; <0.01)*** |  | -0.03 (-0.14; 0.09) |
| Sex, female/male |  | **0.32 (0.16; 0.60)**** |  | **0.22 (0.14; 0.28)**** |  | **0.23 (0.10; 0.37)**** |  | **0.19 (0.06; 0.30)*** |  | **0.21 (0.06; 0.32)*** |
| Ethnicity, Black/White |  | **-0.21 (-0.51; -0.05)*** |  | **-0.20 (-0.28; -0.12)**** |  | **-0.17 (-0.31; -0.03)*** |  | **-0.25 (-0.37; -0.11)**** |  | **-0.18 (-0.32; -0.02)*** |
| Protein intake, g |  | -0.02 (-0.19; 0.16) |  | 0.03 (-0.03; 0.09) |  | 0.01 (-0.10; 0.13) |  | 0.08 (-0.02; 0.18) |  | -0.01 (-0.11; 0.10) |
| Waist-to-height ratio |  | 0.03 (-0.18; 0.27) |  | 0.04 (-0.02; 0.11) |  | 0.08 (-0.04; 0.29) |  | 0.02 (-0.11; 0.16) |  | -0.02 (-0.12; 0.09) |
| Physical act, kCal/kg/day |  | 0.04 (-0.18; 0.30) |  | 0.03 (-0.02; 0.09) |  | 0.02 (-0.10; 0.16) |  | 0.06 (-0.04; 0.14) |  | 0.01 (-0.09; 0.10) |
| Cotinine, ng/ml |  |  |  | **-0.06 (-0.12; <0.01)*** |  | -0.03 (-0.20; 0.11) |  | -0.02 (-0.12; 0.08) |  | **-0.14 (-0.21; -0.01)*** |
| GGT, U/L |  | **-0.35 (-0.72; -0.25)**** |  | **-0.26 (-0.32; -0.19)**** |  | **-0.27 (-0.44; -0.17)**** |  | **-0.21 (-0.35; -0.10)**** |  | **-0.24 (-0.33; -0.11)**** |
| 24h Systolic BP, mmHg |  | -0.09 (-0.40; 0.13) |  | -0.02 (-0.09; 0.05) |  | 0.01 (-0.13; 0.15) |  | -0.02 (-0.14; 0.11) |  | -0.03 (-0.15; 0.09) |
| HbA1c, (%) |  | 0.01 (-0.21; 0.22) |  | -0.04 (-0.10; 0.02) |  | -0.02 (-0.15; 0.10) |  | -0.05 (-0.15; 0.05) |  | -0.05 (-0.15; 0.07) |
| LDL cholesterol, mmol/L |  | **-0.33 (-0.83; -0.29)**** |  | **-0.31 (-0.36; -0.23)**** |  | **-0.28 (-0.47; -0.20)**** |  | **-0.35 (-0.45; -0.23)**** |  | **-0.31 (-0.34; -0.14)**** |
| SES score |  | 0.07 (-0.19; 0.45) |  | -0.01 (-0.08; 0.06) |  | -0.07 (-0.23; 0.07) |  | 0.04 (-0.09; 0.17) |  | <0.01 (-0.13; 0.13) |
| Butyrylcarnitine, AU | **0.43**** | **0.15 (0.01; 0.35)*** | **0.32**** | **0.10 (0.05; 0.16)**** | **0.33**** | **0.14 (0.05; 0.24)*** | **0.33**** | **0.11 (0.02; 0.21)*** | **0.23**** | 0.04 (-0.06; 0.13) |
| Age, years |  | **-0.17 (-0.39; -0.01)*** |  | **-0.09 (-0.15; -0.02)*** |  | -0.09 (-0.20; 0.02) |  | **-0.12 (-0.23; -0.01)*** |  | -0.02 (-0.13; 0.09) |
| Sex, female/male |  | **0.34 (0.20; 0.61)**** |  | **0.24 (0.17; 0.30)**** |  | **0.25 (0.14; 0.38)**** |  | **0.21 (0.09; 0.31)**** |  | **0.23 (0.09; 0.34)**** |
| Ethnicity, Black/White |  | **-0.21 (-0.50; -0.07)*** |  | **-0.20 (-0.27; -0.12)**** |  | **-0.17 (-0.31; -0.05)*** |  | **-0.25 (-0.37; -0.12)**** |  | **-0.16 (-0.29; -0.01)*** |
| Protein intake, g |  | -0.04 (-0.21; 0.13) |  | 0.04 (-0.02; 0.09) |  | 0.02 (-0.09; 0.13) |  | 0.08 (-0.02; 0.17) |  | -0.01 (-0.11; 0.09) |
| Waist-to-height ratio |  | 0.03 (-0.16; 0.27) |  | 0.05 (-0.02; 0.11) |  | 0.09 (-0.02; 0.29) |  | 0.02 (-0.10; 0.16) |  | -0.04 (-0.13; 0.07) |
| Physical act, kCal/kg/day |  | 0.03 (-0.19; 0.27) |  | 0.03 (-0.03; 0.08) |  | 0.01 (-0.11; 0.13) |  | 0.05 (-0.04; 0.13) |  | 0.01 (-0.08; 0.10) |
| Cotinine, ng/ml |  |  |  | **-0.06 (-0.11; -0.01)*** |  | -0.02 (-0.18; 0.11) |  | -0.02 (-0.11; 0.08) |  | **-0.15 (-0.21; -0.02)*** |
| GGT, U/L |  | **-0.35 (-0.71; -0.26)**** |  | **-0.25 (-0.31; -0.18)**** |  | **-0.26 (-0.43; -0.17)**** |  | **-0.21 (-0.35; -0.12)**** |  | **-0.23 (-0.31; -0.10)**** |
| 24h Systolic BP, mmHg |  | -0.11 (-0.41; 0.10) |  | -0.02 (-0.08; 0.05) |  | 0.02 (-0.12; 0.15) |  | -0.03 (-0.14; 0.09) |  | -0.03 (-0.14; 0.09) |
| HbA1c, (%) |  | 0.01 (-0.20; 0.21) |  | -0.05 (-0.10; 0.01) |  | -0.03 (-0.16; 0.09) |  | -0.05 (-0.14; 0.05) |  | -0.05 (-0.15; 0.06) |
| LDL cholesterol, mmol/L |  | **-0.31 (-0.79; -0.27)**** |  | **-0.31 (-0.35; -0.24)**** |  | **-0.28 (-0.46; -0.2)**** |  | **-0.34 (-0.44; -0.23)**** |  | **-0.31 (-0.34; -0.14)**** |
| SES score |  | 0.05 (-0.22; 0.40) |  | -0.01 (-0.08; 0.06) |  | -0.08 (-0.24; 0.04) |  | 0.06 (-0.07; 0.18) |  | 0.01 (-0.11; 0.13) |
| Isovalerylcarnitine, AU | **0.41**** | 0.10 (-0.07; 0.32) | **0.33**** | **0.16 (0.09; 0.21)**** | **0.34**** | **0.19 (0.09; 0.29)**** | **0.34**** | **0.15 (0.04; 0.23)*** | **0.25**** | **0.12 (0.01; 0.21)*** |
| Age, years |  | **-0.18 (-0.41; -0.02)*** |  | **-0.08 (-0.14; -0.02)*** |  | -0.08 (-0.20; 0.03) |  | **-0.13 (-0.23; -0.01)*** |  | -0.02 (-0.12; 0.09) |
| Sex, female/male |  | **0.33 (0.18; 0.60)**** |  | **0.22 (0.14; 0.28)**** |  | **0.22 (0.10; 0.35)**** |  | **0.18 (0.06; 0.28)*** |  | **0.22 (0.08; 0.32)**** |
| Ethnicity, Black/White |  | **-0.21 (-0.51; -0.06)*** |  | **-0.22 (-0.29; -0.14)**** |  | **-0.19 (-0.33; -0.06)*** |  | **-0.27 (-0.38; -0.13)**** |  | **-0.17 (-0.31; -0.03)*** |
| Protein intake, g |  | -0.03 (-0.21; 0.14) |  | 0.04 (-0.02; 0.10) |  | 0.02 (-0.08; 0.14) |  | 0.08 (-0.01; 0.18) |  | <0.01 (-0.10; 0.10) |
| Waist-to-height ratio |  | 0.03 (-0.17; 0.27) |  | 0.03 (-0.04; 0.09) |  | 0.06 (-0.06; 0.25) |  | 0.01 (-0.11; 0.14) |  | -0.04 (-0.13; 0.07) |
| Physical act, kCal/kg/day |  | 0.02 (-0.19; 0.27) |  | 0.03 (-0.03; 0.08) |  | 0.02 (-0.10; 0.14) |  | 0.05 (-0.05; 0.13) |  | <0.01 (-0.09; 0.09) |
| Cotinine, ng/ml |  |  |  | **-0.07 (-0.12; -0.01)*** |  | -0.03 (-0.18; 0.11) |  | -0.02 (-0.11; 0.08) |  | **-0.16 (-0.22; -0.03)*** |
| GGT, U/L |  | **-0.37 (-0.73; -0.27)**** |  | **-0.25 (-0.31; -0.18)**** |  | **-0.26 (-0.42; -0.16)**** |  | **-0.21 (-0.35; -0.12)**** |  | **-0.23 (-0.31; -0.10)**** |
| 24h Systolic BP, mmHg |  | -0.10 (-0.40; 0.11) |  | -0.02 (-0.09; 0.05) |  | 0.01 (-0.12; 0.14) |  | -0.02 (-0.14; 0.09) |  | -0.03 (-0.14; 0.09) |
| HbA1c, (%) |  | <0.01 (-0.20; 0.21) |  | -0.04 (-0.10; 0.02) |  | -0.02 (-0.14; 0.10) |  | -0.05 (-0.15; 0.04) |  | -0.05 (-0.15; 0.06) |
| LDL cholesterol, mmol/L |  | **-0.32 (-0.81; -0.27)**** |  | **-0.32 (-0.36; -0.24)**** |  | **-0.28 (-0.46; -0.21)**** |  | **-0.34 (-0.44; -0.23)**** |  | **-0.32 (-0.35; -0.15)**** |
| SES score |  | 0.06 (-0.20; 0.43) |  | -0.01 (-0.08; 0.05) |  | -0.08 (-0.24; 0.05) |  | 0.05 (-0.08; 0.17) |  | -0.01 (-0.13; 0.12) |
| Hexanoylcarnitine, AU | **0.41**** | 0.07 (-0.10; 0.27) | **0.31**** | 0.03 (-0.02; 0.08) | **0.32**** | 0.08 (-0.01; 0.18) | **0.32**** | 0.07 (-0.03; 0.17) | **0.23**** | -0.05 (-0.13; 0.05) |
| Age, years |  | **-0.17 (-0.40; <0.01)*** |  | **-0.09 (-0.15; -0.02)*** |  | -0.09 (-0.21; 0.02) |  | **-0.12 (-0.23; -0.01)*** |  | -0.02 (-0.12; 0.10) |
| Sex, female/male |  | **0.35 (0.20; 0.63)**** |  | **0.25 (0.18; 0.31)**** |  | **0.27 (0.15; 0.40)**** |  | **0.22 (0.09; 0.32)**** |  | **0.23 (0.08; 0.33)**** |
| Ethnicity, Black/White |  | **-0.20 (-0.50; -0.04)*** |  | **-0.19 (-0.26; -0.11)**** |  | **-0.15 (-0.29; -0.02)*** |  | **-0.24 (-0.36; -0.11)**** |  | **-0.15 (-0.29; -0.01)*** |
| Protein intake, g |  | -0.01 (-0.18; 0.16) |  | 0.04 (-0.02; 0.10) |  | 0.03 (-0.08; 0.14) |  | 0.09 (-0.01; 0.19) |  | -0.02 (-0.12; 0.09) |
| Waist-to-height ratio |  | 0.04 (-0.16; 0.29) |  | 0.04 (-0.02; 0.11) |  | 0.10 (-0.01; 0.30) |  | 0.03 (-0.10; 0.16) |  | -0.05 (-0.14; 0.06) |
| Physical act, kCal/kg/day |  | 0.03 (-0.18; 0.29) |  | 0.03 (-0.02; 0.08) |  | 0.02 (-0.10; 0.14) |  | 0.05 (-0.04; 0.13) |  | 0.02 (-0.08; 0.10) |
| Cotinine, ng/ml |  |  |  | **-0.08 (-0.13; -0.02)*** |  | -0.03 (-0.19; 0.11) |  | -0.03 (-0.13; 0.07) |  | **-0.15 (-0.22; -0.03)*** |
| GGT, U/L |  | **-0.36 (-0.73; -0.27)**** |  | **-0.25 (-0.32; -0.18)**** |  | **-0.26 (-0.42; -0.16)**** |  | **-0.21 (-0.34; -0.11)**** |  | **-0.23 (-0.32; -0.10)**** |
| 24h Systolic BP, mmHg |  | -0.10 (-0.41; 0.12) |  | -0.01 (-0.08; 0.05) |  | 0.01 (-0.13; 0.15) |  | -0.02 (-0.13; 0.10) |  | -0.02 (-0.14; 0.10) |
| HbA1c, (%) |  | 0.01 (-0.20; 0.23) |  | -0.04 (-0.10; 0.02) |  | -0.02 (-0.15; 0.10) |  | -0.05 (-0.15; 0.05) |  | -0.05 (-0.15; 0.06) |
| LDL cholesterol, mmol/L |  | **-0.33 (-0.83; -0.29)**** |  | **-0.32 (-0.36; -0.24)**** |  | **-0.29 (-0.47; -0.21)**** |  | **-0.35 (-0.45; -0.23)**** |  | **-0.31 (-0.34; -0.14)**** |
| SES score |  | 0.06 (-0.20; 0.44) |  | <0.01 (-0.07; 0.07) |  | -0.07 (-0.23; 0.06) |  | 0.06 (-0.07; 0.19) |  | 0.01 (-0.11; 0.14) |

Test used: Multiple linear regressions. Data are presented as adjusted R^2^ with β coefficient and 95% confidence intervals. Estimated glomerular filtration rate (cystatin C-based), adjusted for age, sex, ethnicity, protein intake, waist-to-hight ratio, physical activity, cotinine, GGT, 24h systolic BP, HbA1c, LDL, SES score. Bold values denote P≤0.05; *P≤0.05; **P≤0.001. Cardiovascular disease risk group criteria: Obese - ≥0.55 waist-to-height ratio; Physically inactive - <600 METs for moderate and/or vigorous intensity physical activity; Smoking - ≥11 ng/mL cotinine & self-reported smoking; Excessive alcohol intake - ≥49 U/L GGT & self-reported drinking; Masked hypertensive – normal clinic BP & 24h/day/night BP classified as hypertensive; Hyperglycemic - ≥5.7% HbA1c; Dyslipidemic - >3.4 mmol/L LDL; Low socio-economic – low SES.

*Abbreviations*: AU, arbitrary units; physical act, physical activity; GGT, gamma-glutamyl transferase; BP, blood pressure; HbA1c, glycated haemoglobin; LDL, low density lipoprotein; SES, socio-economic status; GFR, estimated glomerular filtration rate; CVD, cardiovascular disease.

**Supplementary Table 2J. Multi-variable adjusted regression analysis with estimated glomerular filtration rate (cystatin C-based) as the dependent variable, with the metabolomics data in control, cardiovascular disease risk group and cardiovascular disease risk clusters**

|  | **eGFR (cystatin-C), ml/min/1.73m^2^** | | | | | | | | | |
| --- | --- | --- | --- | --- | --- | --- | --- | --- | --- | --- |
|  | **Control group**  **(N=166)** | | **CVD risk group**  **(N=1036)** | | **1 CVD risk factor**  **(N=344)** | | **2 CVD risk factors (N=360)** | | **3+ CVD risk factors (N=332)** | |
| ***Metabolomic data*** | **Adj R^2^** | **β (95%Cl)** | **Adj R^2^** | **β (95%Cl)** | **Adj R^2^** | **β (95%Cl)** | **Adj R^2^** | **β (95%Cl)** | **Adj R^2^** | **β (95%Cl)** |
| Octanoylcarnitine, AU | **0.41**** | 0.05 (-0.13; 0.26) | **0.32**** | **0.06 (0.01; 0.12)*** | **0.32**** | 0.07 (-0.03; 0.19) | **0.33**** | **0.12 (0.02; 0.22)*** | **0.23**** | <0.01 (-0.09; 0.08) |
| Age, years |  | **-0.17 (-0.40; -0.01)*** |  | **-0.09 (-0.15; -0.02)*** |  | -0.09 (-0.21; 0.02) |  | **-0.13 (-0.23; -0.02)*** |  | -0.02 (-0.13; 0.09) |
| Sex, female/male |  | **0.35 (0.20; 0.62)**** |  | **0.24 (0.17; 0.30)**** |  | **0.26 (0.14; 0.39)**** |  | **0.20 (0.08; 0.30)**** |  | **0.23 (0.09; 0.33)**** |
| Ethnicity, Black/White |  | **-0.19 (-0.48; -0.04)*** |  | **-0.19 (-0.26; -0.11)**** |  | **-0.16 (-0.29; -0.03)*** |  | **-0.26 (-0.38; -0.13)**** |  | **-0.16 (-0.29; -0.01)*** |
| Protein intake, g |  | -0.01 (-0.19; 0.15) |  | 0.05 (-0.01; 0.10) |  | 0.03 (-0.08; 0.14) |  | **0.10 (<0.01; 0.20)*** |  | -0.01 (-0.11; 0.09) |
| Waist-to-height ratio |  | 0.04 (-0.16; 0.28) |  | 0.04 (-0.02; 0.10) |  | 0.09 (-0.02; 0.30) |  | 0.03 (-0.10; 0.16) |  | -0.05 (-0.13; 0.06) |
| Physical act, kCal/kg/day |  | 0.02 (-0.20; 0.27) |  | 0.03 (-0.03; 0.08) |  | 0.02 (-0.10; 0.15) |  | 0.05 (-0.05; 0.13) |  | 0.01 (-0.08; 0.10) |
| Cotinine, ng/ml |  |  |  | **-0.07 (-0.12; -0.01)*** |  | -0.03 (-0.19; 0.10) |  | -0.03 (-0.12; 0.07) |  | **-0.16 (-0.22; -0.03)*** |
| GGT, U/L |  | **-0.36 (-0.73; -0.27)**** |  | **-0.25 (-0.31; -0.18)**** |  | **-0.26 (-0.43; -0.16)**** |  | **-0.21 (-0.34; -0.11)**** |  | **-0.23 (-0.31; -0.10)**** |
| 24h Systolic BP, mmHg |  | -0.09 (-0.39; 0.12) |  | -0.02 (-0.08; 0.05) |  | <0.01 (-0.13; 0.14) |  | -0.01 (-0.13; 0.10) |  | -0.02 (-0.14; 0.10) |
| HbA1c, (%) |  | 0.01 (-0.19; 0.23) |  | -0.04 (-0.10; 0.02) |  | -0.02 (-0.14; 0.10) |  | -0.05 (-0.14; 0.05) |  | -0.05 (-0.15; 0.06) |
| LDL cholesterol, mmol/L |  | **-0.34 (-0.84; -0.32)**** |  | **-0.32 (-0.36; -0.24)**** |  | **-0.29 (-0.47; -0.21)**** |  | **-0.34 (-0.44; -0.23)**** |  | **-0.31 (-0.34; -0.14)**** |
| SES score |  | 0.06 (-0.19; 0.43) |  | <0.01 (-0.07; 0.06) |  | -0.07 (-0.23; 0.06) |  | 0.07 (-0.06; 0.19) |  | 0.01 (-0.11; 0.13) |
| Decanoylcarnitine, AU | **0.41**** | 0.05 (-0.12; 0.23) | **0.32**** | **0.08 (0.02; 0.13)*** | **0.32**** | 0.08 (-0.02; 0.19) | **0.33**** | **0.12 (0.02; 0.21)*** | **0.23**** | 0.05 (-0.05; 0.13) |
| Age, years |  | **-0.17 (-0.39; -0.01)*** |  | **-0.08 (-0.14; -0.02)*** |  | -0.09 (-0.21; 0.02) |  | **-0.12 (-0.22; -0.01)*** |  | -0.02 (-0.13; 0.09) |
| Sex, female/male |  | **0.34 (0.20; 0.62)**** |  | **0.24 (0.17; 0.30)**** |  | **0.26 (0.14; 0.39)**** |  | **0.19 (0.07; 0.30)**** |  | **0.23 (0.09; 0.33)**** |
| Ethnicity, Black/White |  | **-0.19 (-0.48; -0.04)*** |  | **-0.19 (-0.26; -0.11)**** |  | **-0.15 (-0.29; -0.03)*** |  | **-0.26 (-0.38; -0.13)**** |  | **-0.16 (-0.29; -0.01)*** |
| Protein intake, g |  | -0.02 (-0.19; 0.15) |  | 0.04 (-0.02; 0.10) |  | 0.03 (-0.08; 0.14) |  | **0.11 (<0.01; 0.20)*** |  | -0.01 (-0.11; 0.09) |
| Waist-to-height ratio |  | 0.04 (-0.16; 0.28) |  | 0.05 (-0.02; 0.11) |  | 0.10 (-0.01; 0.30) |  | 0.03 (-0.09; 0.16) |  | -0.04 (-0.13; 0.07) |
| Physical act, kCal/kg/day |  | 0.03 (-0.19; 0.28) |  | 0.03 (-0.03; 0.08) |  | 0.02 (-0.10; 0.15) |  | 0.05 (-0.05; 0.13) |  | 0.01 (-0.08; 0.09) |
| Cotinine, ng/ml |  |  |  | **-0.07 (-0.12; -0.01)*** |  | -0.03 (-0.19; 0.10) |  | -0.02 (-0.11; 0.07) |  | **-0.15 (-0.21; -0.03)*** |
| GGT, U/L |  | **-0.37 (-0.73; -0.27)**** |  | **-0.25 (-0.31; -0.18)**** |  | **-0.26 (-0.43; -0.16)**** |  | **-0.20 (-0.34; -0.11)**** |  | **-0.22 (-0.31; -0.10)**** |
| 24h Systolic BP, mmHg |  | -0.09 (-0.40; 0.12) |  | -0.02 (-0.09; 0.05) |  | <0.01 (-0.14; 0.14) |  | -0.02 (-0.13; 0.10) |  | -0.03 (-0.14; 0.09) |
| HbA1c, (%) |  | 0.01 (-0.19; 0.23) |  | -0.04 (-0.10; 0.02) |  | -0.02 (-0.14; 0.10) |  | -0.05 (-0.15; 0.05) |  | -0.05 (-0.15; 0.06) |
| LDL cholesterol, mmol/L |  | **-0.33 (-0.83; -0.30)**** |  | **-0.32 (-0.36; -0.24)**** |  | **-0.29 (-0.47; -0.22)**** |  | **-0.34 (-0.44; -0.23)**** |  | **-0.31 (-0.34; -0.14)**** |
| SES score |  | 0.06 (-0.20; 0.43) |  | -0.01 (-0.07; 0.06) |  | -0.07 (-0.23; 0.06) |  | 0.06 (-0.07; 0.18) |  | <0.01 (-0.12; 0.13) |
| Dodecanoylcarnitine, AU | 0.23 | -0.13 (-0.60; 0.27) | **0.29**** | -0.09 (-0.19; 0.02) | **0.23*** | -0.11 (-0.31; 0.09) | **0.23*** | 0.14 (-0.09; 0.45) | **0.22*** | **-0.32 (-0.43; -0.08)*** |
| Age, years |  | -0.12 (-0.59; 0.30) |  | -0.08 (-0.20; 0.04) |  | -0.08 (-0.31; 0.15) |  | -0.08 (-0.32; 0.16) |  | 0.02 (-0.19; 0.22) |
| Sex, female/male |  | 0.30 (-0.12; 0.84) |  | **0.24 (0.10; 0.36)**** |  | **0.26 (0.01; 0.52)*** |  | 0.23 (-0.02; 0.47) |  | 0.18 (-0.07; 0.40) |
| Ethnicity, Black/White |  | -0.21 (-0.78; 0.21) |  | **-0.19 (-0.33; -0.04)*** |  | -0.15 (-0.42; 0.12) |  | -0.23 (-0.48; 0.05) |  | -0.21 (-0.46; 0.07) |
| Protein intake, g |  | 0.02 (-0.37; 0.41) |  | 0.03 (-0.08; 0.14) |  | 0.02 (-0.21; 0.24) |  | 0.11 (-0.11; 0.32) |  | -0.07 (-0.25; 0.13) |
| Waist-to-height ratio |  | 0.05 (-0.41; 0.57) |  | 0.03 (-0.09; 0.15) |  | 0.09 (-0.19; 0.45) |  | 0.02 (-0.26; 0.31) |  | -0.13 (-0.28; 0.10) |
| Physical act, kCal/kg/day |  | 0.01 (-0.50; 0.54) |  | 0.04 (-0.06; 0.14) |  | 0.03 (-0.21; 0.28) |  | 0.04 (-0.16; 0.22) |  | 0.05 (-0.13; 0.21) |
| Cotinine, ng/ml |  |  |  | -0.08 (-0.18; 0.04) |  | -0.05 (-0.38; 0.22) |  | -0.06 (-0.27; 0.15) |  | -0.15 (-0.29; 0.06) |
| GGT, U/L |  | -0.36 (-1.01; 0.01) |  | **-0.26 (-0.39; -0.13)**** |  | **-0.27 (-0.57; -0.05)*** |  | -0.19 (-0.47; 0.04) |  | **-0.29 (-0.47; -0.06)*** |
| 24h Systolic BP, mmHg |  | -0.07 (-0.68; 0.47) |  | 0.01 (-0.13; 0.14) |  | 0.03 (-0.24; 0.31) |  | -0.02 (-0.28; 0.23) |  | 0.11 (-0.14; 0.33) |
| HbA1c, (%) |  | -0.04 (-0.55; 0.44) |  | -0.05 (-0.16; 0.07) |  | -0.05 (-0.31; 0.20) |  | -0.05 (-0.25; 0.16) |  | -0.06 (-0.25; 0.14) |
| LDL cholesterol, mmol/L |  | -0.36 (-1.19; -0.03)* |  | **-0.31 (-0.41; -0.17)**** |  | **-0.31 (-0.63; -0.11)*** |  | **-0.37 (-0.60; -0.13)*** |  | -0.20 (-0.35; 0.04) |
| SES score |  | 0.06 (-0.57; 0.81) |  | <0.01 (-0.14; 0.13) |  | -0.07 (-0.37; 0.21) |  | 0.04 (-0.23; 0.31) |  | 0.01 (-0.22; 0.24) |

Test used: Multiple linear regressions. Data are presented as adjusted R^2^ with β coefficient and 95% confidence intervals. Estimated glomerular filtration rate (cystatin C-based), adjusted for age, sex, ethnicity, protein intake, waist-to-hight ratio, physical activity, cotinine, GGT, 24h systolic BP, HbA1c, LDL, SES score. Bold values denote P≤0.05; *P≤0.05; **P≤0.001. Cardiovascular disease risk group criteria: Obese - ≥0.55 waist-to-height ratio; Physically inactive - <600 METs for moderate and/or vigorous intensity physical activity; Smoking - ≥11 ng/mL cotinine & self-reported smoking; Excessive alcohol intake - ≥49 U/L GGT & self-reported drinking; Masked hypertensive – normal clinic BP & 24h/day/night BP classified as hypertensive; Hyperglycemic - ≥5.7% HbA1c; Dyslipidemic - >3.4 mmol/L LDL; Low socio-economic – low SES.

*Abbreviations*: AU, arbitrary units; physical act, physical activity; GGT, gamma-glutamyl transferase; BP, blood pressure; HbA1c, glycated haemoglobin; LDL, low density lipoprotein; SES, socio-economic status; GFR, estimated glomerular filtration rate; CVD, cardiovascular disease.

**Supplementary Table 3A. Supplementary analysis with estimated glomerular filtration rate (creatinine-based) as the dependent variable, with the metabolomics data in control, cardiovascular disease risk group and cardiovascular disease risk clusters**

|  | **eGFR (creatinine), ml/min/1.73m^2^** | | | | | | | | | |
| --- | --- | --- | --- | --- | --- | --- | --- | --- | --- | --- |
|  | **Control group**  **(N=166)** | | **CVD risk group**  **(N=1036)** | | **1 CVD risk factor**  **(N=344)** | | **2 CVD risk factors (N=360)** | | **3+ CVD risk factors (N=332)** | |
| ***Metabolomic data*** | **Adj R^2^** | **β (95%Cl)** | **Adj R^2^** | **β (95%Cl)** | **Adj R^2^** | **β (95%Cl)** | **Adj R^2^** | **β (95%Cl)** | **Adj R^2^** | **β (95%Cl)** |
| Ornithine, AU | 0.19 | <0.01 (-0.32; 0.33) | **0.26**** | <0.01 (-0.09; 0.08) | **0.28**** | -0.07 (-0.26; 0.12) | **0.27**** | -0.10 (-0.26; 0.05) | **0.16**** | 0.13 (-0.03; 0.26) |
| Age, years |  | -0.22 (-0.63; 0.13) |  | **-0.20 (-0.29; -0.10)**** |  | **-0.22 (-0.41; -0.03)*** |  | **-0.19 (-0.39; -0.01)*** |  | -0.17 (-0.29; 0.01) |
| Sex, female/male |  | 0.14 (-0.25; 0.57) |  | 0.07 (-0.03; 0.18) |  | 0.06 (-0.17; 0.28) |  | 0.06 (-0.13; 0.26) |  | 0.09 (-0.09; 0.25) |
| Ethnicity, Black/White |  | -0.13 (-0.61; 0.26) |  | **-0.16 (-0.27; -0.04)*** |  | **-0.26 (-0.49; -0.04)*** |  | -0.19 (-0.41; 0.01) |  | -0.04 (-0.23; 0.16) |
| Protein intake, g |  | -0.06 (-0.40; 0.26) |  | -0.02 (-0.11; 0.07) |  | 0.04 (-0.15; 0.23) |  | -0.05 (-0.22; 0.11) |  | -0.05 (-0.18; 0.09) |
| Waist-to-height ratio |  | 0.11 (-0.27; 0.60) |  | **0.16 (0.06; 0.26)*** |  | 0.11 (-0.10; 0.42) |  | 0.04 (-0.17; 0.27) |  | **0.21 (0.01; 0.28)*** |
| Physical act, kCal/kg/day |  | -0.04 (-0.52; 0.39) |  | 0.01 (-0.07; 0.09) |  | 0.04 (-0.15; 0.26) |  | <0.01 (-0.15; 0.15) |  | <0.01 (-0.12; 0.12) |
| Cotinine, ng/ml |  |  |  | 0.07 (-0.02; 0.15) |  | 0.02 (-0.22; 0.29) |  | 0.06 (-0.10; 0.23) |  | 0.05 (-0.10; 0.16) |
| GGT, U/L |  | -0.31 (-0.87; 0.03) |  | **-0.24 (-0.34; -0.14)**** |  | **-0.34 (-0.61; -0.16)**** |  | **-0.22 (-0.47; -0.07)*** |  | -0.15 (-0.28; 0.02) |
| 24h Systolic BP, mmHg |  | -0.04 (-0.56; 0.45) |  | 0.02 (-0.09; 0.12) |  | 0.03 (-0.20; 0.26) |  | 0.02 (-0.17; 0.23) |  | -0.04 (-0.19; 0.13) |
| HbA1c, (%) |  | 0.01 (-0.40; 0.42) |  | -0.03 (-0.12; 0.06) |  | -0.06 (-0.27; 0.14) |  | -0.07 (-0.24; 0.09) |  | <0.01 (-0.15; 0.14) |
| LDL cholesterol, mmol/L |  | -0.36 (-1.10; -0.07)* |  | **-0.26 (-0.34; -0.15)**** |  | **-0.26 (-0.52; -0.09)*** |  | **-0.27 (-0.48; -0.11)**** |  | **-0.30 (-0.36; -0.08)**** |
| SES score |  | 0.06 (-0.50; 0.73) |  | -0.09 (-0.20; 0.02) |  | -0.05 (-0.31; 0.18) |  | -0.05 (-0.27; 0.16) |  | -0.10 (-0.25; 0.08) |
| 5-Hydroxylysine, AU | **0.32**** | -0.01 (-0.20; 0.17) | **0.27**** | -0.03 (-0.09; 0.04) | **0.32**** | -0.04 (-0.16; 0.07) | **0.29**** | <0.01 (-0.12; 0.12) | **0.17**** | -0.05 (-0.14; 0.06) |
| Age, years |  | **-0.21 (-0.48; -0.02)*** |  | **-0.2 (-0.27; -0.13)**** |  | **-0.21 (-0.34; -0.09)**** |  | **-0.21 (-0.36; -0.08)**** |  | **-0.15 (-0.25; -0.01)*** |
| Sex, female/male |  | 0.14 (-0.08; 0.40) |  | 0.07 (-0.01; 0.15) |  | 0.03 (-0.11; 0.16) |  | 0.05 (-0.10; 0.19) |  | 0.11 (-0.04; 0.23) |
| Ethnicity, Black/White |  | -0.13 (-0.42; 0.08) |  | **-0.16 (-0.24; -0.07)**** |  | **-0.25 (-0.40; -0.11)**** |  | **-0.19 (-0.36; -0.04)*** |  | -0.04 (-0.19; 0.12) |
| Protein intake, g |  | -0.06 (-0.27; 0.12) |  | -0.02 (-0.09; 0.05) |  | 0.04 (-0.08; 0.16) |  | -0.04 (-0.17; 0.08) |  | -0.05 (-0.16; 0.07) |
| Waist-to-height ratio |  | 0.11 (-0.09; 0.42) |  | **0.17 (0.09; 0.23)**** |  | 0.11 (-0.01; 0.34) |  | 0.05 (-0.11; 0.22) |  | **0.19 (0.02; 0.24)*** |
| Physical act, kCal/kg/day |  | -0.04 (-0.33; 0.20) |  | 0.01 (-0.05; 0.07) |  | 0.05 (-0.07; 0.20) |  | <0.01 (-0.11; 0.11) |  | 0.01 (-0.09; 0.11) |
| Cotinine, ng/ml |  |  |  | **0.07 (<0.01; 0.13)*** |  | 0.04 (-0.10; 0.22) |  | 0.06 (-0.06; 0.19) |  | 0.03 (-0.08; 0.12) |
| GGT, U/L |  | **-0.31 (-0.68; -0.15)*** |  | **-0.24 (-0.32; -0.16)**** |  | **-0.33 (-0.51; -0.23)**** |  | **-0.21 (-0.40; -0.10)**** |  | **-0.17 (-0.26; -0.03)*** |
| 24h Systolic BP, mmHg |  | -0.04 (-0.35; 0.24) |  | 0.02 (-0.06; 0.10) |  | 0.02 (-0.12; 0.18) |  | 0.02 (-0.13; 0.17) |  | -0.02 (-0.14; 0.12) |
| HbA1c, (%) |  | 0.01 (-0.23; 0.25) |  | -0.03 (-0.10; 0.04) |  | -0.05 (-0.20; 0.07) |  | -0.07 (-0.20; 0.05) |  | <0.01 (-0.11; 0.12) |
| LDL cholesterol, mmol/L |  | **-0.36 (-0.89; -0.29)**** |  | **-0.26 (-0.32; -0.18)**** |  | **-0.27 (-0.46; -0.18)**** |  | **-0.26 (-0.42; -0.15)**** |  | **-0.31 (-0.33; -0.12)**** |
| SES score |  | 0.06 (-0.25; 0.47) |  | **-0.09 (-0.17; -0.01)*** |  | -0.07 (-0.24; 0.08) |  | -0.06 (-0.22; 0.10) |  | -0.10 (-0.21; 0.05) |
| Histidine, AU | **0.39**** | **0.24 (0.11; 0.43)**** | **0.30**** | **0.17 (0.11; 0.23)**** | **0.35**** | **0.14 (0.04; 0.24)*** | **0.34**** | **0.21 (0.12; 0.32)**** | **0.21**** | **0.16 (0.04; 0.24)*** |
| Age, years |  | **-0.20 (-0.42; -0.04)*** |  | **-0.20 (-0.26; -0.14)**** |  | **-0.20 (-0.32; -0.10)**** |  | **-0.21 (-0.34; -0.10)**** |  | **-0.17 (-0.24; -0.04)*** |
| Sex, female/male |  | 0.09 (-0.10; 0.32) |  | 0.04 (-0.03; 0.11) |  | <0.01 (-0.12; 0.12) |  | 0.02 (-0.10; 0.14) |  | 0.09 (-0.04; 0.19) |
| Ethnicity, Black/White |  | -0.13 (-0.38; 0.05) |  | **-0.14 (-0.21; -0.06)**** |  | **-0.24 (-0.37; -0.11)**** |  | **-0.17 (-0.32; -0.05)*** |  | -0.01 (-0.14; 0.13) |
| Protein intake, g |  | -0.06 (-0.24; 0.10) |  | -0.01 (-0.07; 0.05) |  | 0.05 (-0.06; 0.16) |  | -0.04 (-0.15; 0.06) |  | -0.04 (-0.12; 0.07) |
| Waist-to-height ratio |  | 0.09 (-0.09; 0.35) |  | **0.16 (0.09; 0.22)**** |  | 0.09 (-0.02; 0.29) |  | 0.05 (-0.07; 0.21) |  | **0.20 (0.04; 0.23)*** |
| Physical act, kCal/kg/day |  | -0.04 (-0.29; 0.17) |  | 0.02 (-0.04; 0.07) |  | 0.04 (-0.07; 0.17) |  | 0.01 (-0.09; 0.11) |  | 0.02 (-0.07; 0.10) |
| Cotinine, ng/ml |  |  |  | **0.09 (0.03; 0.14)*** |  | 0.06 (-0.06; 0.24) |  | 0.08 (-0.03; 0.18) |  | 0.05 (-0.05; 0.13) |
| GGT, U/L |  | **-0.28 (-0.60; -0.15)**** |  | **-0.22 (-0.29; -0.15)**** |  | **-0.31 (-0.48; -0.23)**** |  | **-0.19 (-0.35; -0.10)**** |  | **-0.15 (-0.23; -0.03)*** |
| 24h Systolic BP, mmHg |  | -0.05 (-0.32; 0.19) |  | <0.01 (-0.06; 0.07) |  | 0.04 (-0.09; 0.18) |  | -0.02 (-0.15; 0.11) |  | -0.03 (-0.13; 0.09) |
| HbA1c, (%) |  | 0.02 (-0.18; 0.23) |  | -0.04 (-0.10; 0.02) |  | -0.06 (-0.19; 0.05) |  | -0.09 (-0.20; 0.02) |  | 0.02 (-0.08; 0.11) |
| LDL cholesterol, mmol/L |  | **-0.33 (-0.80; -0.29)**** |  | **-0.24 (-0.29; -0.17)**** |  | **-0.23 (-0.40; -0.14)**** |  | **-0.25 (-0.39; -0.16)**** |  | **-0.28 (-0.30; -0.11)**** |
| SES score |  | 0.05 (-0.21; 0.40) |  | **-0.09 (-0.15; -0.02)*** |  | -0.09 (-0.25; 0.04) |  | -0.02 (-0.16; 0.12) |  | -0.11 (-0.21; 0.02) |
| Lysine, AU | **0.37**** | **0.19 (0.05; 0.37)*** | **0.29**** | **0.10 (0.04; 0.15)**** | **0.34**** | 0.08 (-0.02; 0.19) | **0.31**** | **0.12 (0.02; 0.22)*** | **0.20**** | **0.11 (<0.01; 0.19)*** |
| Age, years |  | **-0.22 (-0.45; -0.07)*** |  | **-0.21 (-0.26; -0.14)**** |  | **-0.21 (-0.32; -0.10)**** |  | **-0.21 (-0.34; -0.10)**** |  | **-0.16 (-0.24; -0.03)*** |
| Sex, female/male |  | 0.15 (-0.03; 0.38) |  | **0.08 (0.01; 0.14)*** |  | 0.02 (-0.10; 0.15) |  | 0.05 (-0.07; 0.18) |  | 0.12 (-0.01; 0.22) |
| Ethnicity, Black/White |  | -0.13 (-0.39; 0.05) |  | **-0.16 (-0.24; -0.09)**** |  | **-0.26 (-0.40; -0.13)**** |  | **-0.20 (-0.35; -0.08)*** |  | -0.03 (-0.16; 0.11) |
| Protein intake, g |  | -0.10 (-0.29; 0.06) |  | -0.02 (-0.08; 0.04) |  | 0.04 (-0.06; 0.16) |  | -0.04 (-0.15; 0.06) |  | -0.04 (-0.13; 0.06) |
| Waist-to-height ratio |  | 0.12 (-0.05; 0.39) |  | **0.16 (0.09; 0.22)**** |  | 0.10 (-0.01; 0.30) |  | 0.04 (-0.10; 0.19) |  | **0.20 (0.04; 0.23)*** |
| Physical act, kCal/kg/day |  | -0.06 (-0.33; 0.13) |  | 0.01 (-0.04; 0.07) |  | 0.05 (-0.07; 0.18) |  | 0.01 (-0.09; 0.11) |  | 0.02 (-0.07; 0.10) |
| Cotinine, ng/ml |  |  |  | **0.08 (0.02; 0.13)*** |  | 0.05 (-0.07; 0.23) |  | 0.07 (-0.04; 0.18) |  | 0.04 (-0.06; 0.12) |
| GGT, U/L |  | **-0.28 (-0.60; -0.14)*** |  | **-0.23 (-0.30; -0.16)**** |  | **-0.32 (-0.49; -0.23)**** |  | **-0.20 (-0.37; -0.11)**** |  | **-0.16 (-0.23; -0.03)*** |
| 24h Systolic BP, mmHg |  | -0.05 (-0.32; 0.19) |  | 0.01 (-0.06; 0.08) |  | 0.04 (-0.09; 0.18) |  | <0.01 (-0.13; 0.13) |  | -0.03 (-0.13; 0.09) |
| HbA1c, (%) |  | -0.01 (-0.22; 0.20) |  | -0.04 (-0.10; 0.03) |  | -0.06 (-0.19; 0.06) |  | -0.08 (-0.19; 0.02) |  | 0.01 (-0.09; 0.11) |
| LDL cholesterol, mmol/L |  | **-0.33 (-0.80; -0.28)**** |  | **-0.25 (-0.30; -0.18)**** |  | **-0.25 (-0.42; -0.16)**** |  | **-0.26 (-0.40; -0.16)**** |  | **-0.30 (-0.31; -0.12)**** |
| SES score |  | 0.05 (-0.23; 0.40) |  | **-0.09 (-0.16; -0.02)*** |  | -0.08 (-0.24; 0.05) |  | -0.03 (-0.18; 0.11) |  | -0.10 (-0.20; 0.03) |

Test used: Multiple linear regressions. Data are presented as adjusted R^2^ with β coefficient and 95% confidence intervals. Estimated glomerular filtration rate (creatinine-based), adjusted for age, sex, ethnicity, protein intake, waist-to-hight ratio, physical activity, cotinine, GGT, 24h systolic BP, HbA1c, LDL, SES score. Bold values denote P≤0.05; *P≤0.05; **P≤0.001. Cardiovascular disease risk group criteria: Obese - ≥0.55 waist-to-height ratio; Physically inactive - <600 METs for moderate and/or vigorous intensity physical activity; Smoking - ≥11 ng/mL cotinine & self-reported smoking; Excessive alcohol intake - ≥49 U/L GGT & self-reported drinking; Masked hypertensive – normal clinic BP & 24h/day/night BP classified as hypertensive; Hyperglycemic - ≥5.7% HbA1c; Dyslipidemic - >3.4 mmol/L LDL; Low socio-economic – low SES.

*Abbreviations*: AU, arbitrary units; physical act, physical activity; GGT, gamma-glutamyl transferase; BP, blood pressure; HbA1c, glycated haemoglobin; LDL, low density lipoprotein; SES, socio-economic status; eGFR, estimated glomerular filtration rate; CVD, cardiovascular disease.

**Supplementary Table 3B. Supplementary analysis with estimated glomerular filtration rate (creatinine-based) as the dependent variable, with the metabolomics data in control, cardiovascular disease risk group and cardiovascular disease risk clusters**

|  | **eGFR (creatinine), ml/min/1.73m^2^** | | | | | | | | | |
| --- | --- | --- | --- | --- | --- | --- | --- | --- | --- | --- |
|  | **Control group**  **(N=166)** | | **CVD risk group**  **(N=1036)** | | **1 CVD risk factor**  **(N=344)** | | **2 CVD risk factors (N=360)** | | **3+ CVD risk factors (N=332)** | |
| ***Metabolomic data*** | **Adj R^2^** | **β (95%Cl)** | **Adj R^2^** | **β (95%Cl)** | **Adj R^2^** | **β (95%Cl)** | **Adj R^2^** | **β (95%Cl)** | **Adj R^2^** | **β (95%Cl)** |
| Arginine, AU | **0.35**** | 0.15 (-0.01; 0.36) | **0.28**** | **0.07 (0.01; 0.12)*** | **0.33**** | 0.03 (-0.07; 0.12) | **0.30**** | 0.09 (-0.01; 0.21) | **0.19**** | 0.07 (-0.03; 0.15) |
| Age, years |  | **-0.20 (-0.43; -0.04)*** |  | **-0.21 (-0.26; -0.14)**** |  | **-0.22 (-0.33; -0.11)**** |  | **-0.21 (-0.34; -0.10)**** |  | **-0.16 (-0.24; -0.03)*** |
| Sex, female/male |  | 0.17 (-0.01; 0.42) |  | **0.09 (0.02; 0.16)*** |  | 0.03 (-0.09; 0.16) |  | 0.07 (-0.05; 0.20) |  | 0.13 (-0.01; 0.23) |
| Ethnicity, Black/White |  | -0.14 (-0.40; 0.04) |  | **-0.16 (-0.23; -0.08)**** |  | **-0.25 (-0.39; -0.13)**** |  | **-0.19 (-0.34; -0.07)*** |  | -0.03 (-0.16; 0.11) |
| Protein intake, g |  | -0.07 (-0.25; 0.09) |  | -0.02 (-0.08; 0.04) |  | 0.04 (-0.07; 0.15) |  | -0.04 (-0.15; 0.06) |  | -0.05 (-0.13; 0.06) |
| Waist-to-height ratio |  | 0.13 (-0.03; 0.41) |  | **0.17 (0.09; 0.22)**** |  | 0.10 (<0.01; 0.31) |  | 0.05 (-0.08; 0.21) |  | **0.20 (0.04; 0.23)*** |
| Physical act, kCal/kg/day |  | -0.05 (-0.31; 0.16) |  | 0.01 (-0.05; 0.06) |  | 0.05 (-0.06; 0.18) |  | <0.01 (-0.10; 0.10) |  | 0.01 (-0.08; 0.09) |
| Cotinine, ng/ml |  |  |  | **0.07 (0.01; 0.13)*** |  | 0.04 (-0.08; 0.21) |  | 0.06 (-0.04; 0.17) |  | 0.04 (-0.06; 0.12) |
| GGT, U/L |  | **-0.29 (-0.62; -0.15)**** |  | **-0.23 (-0.30; -0.17)**** |  | **-0.33 (-0.49; -0.24)**** |  | **-0.20 (-0.38; -0.11)**** |  | **-0.16 (-0.24; -0.03)*** |
| 24h Systolic BP, mmHg |  | -0.06 (-0.35; 0.17) |  | 0.01 (-0.06; 0.08) |  | 0.03 (-0.10; 0.17) |  | 0.01 (-0.12; 0.14) |  | -0.03 (-0.14; 0.09) |
| HbA1c, (%) |  | 0.01 (-0.20; 0.22) |  | -0.04 (-0.10; 0.03) |  | -0.06 (-0.19; 0.06) |  | -0.08 (-0.19; 0.03) |  | 0.01 (-0.09; 0.11) |
| LDL cholesterol, mmol/L |  | **-0.33 (-0.81; -0.28)**** |  | **-0.26 (-0.30; -0.18)**** |  | **-0.26 (-0.43; -0.18)**** |  | **-0.26 (-0.40; -0.16)**** |  | **-0.30 (-0.32; -0.12)**** |
| SES score |  | 0.04 (-0.24; 0.40) |  | **-0.09 (-0.16; -0.02)*** |  | -0.07 (-0.22; 0.06) |  | -0.04 (-0.19; 0.10) |  | -0.10 (-0.20; 0.03) |
| Asparagine, AU | **0.34**** | 0.07 (-0.08; 0.25) | **0.28**** | **0.07 (0.02; 0.13)*** | **0.33**** | 0.04 (-0.06; 0.15) | **0.30**** | 0.09 (-0.01; 0.18) | **0.19**** | 0.08 (-0.03; 0.16) |
| Age, years |  | **-0.21 (-0.44; -0.05)*** |  | **-0.21 (-0.26; -0.14)**** |  | **-0.22 (-0.33; -0.11)**** |  | **-0.21 (-0.34; -0.10)**** |  | **-0.16 (-0.24; -0.03)*** |
| Sex, female/male |  | 0.14 (-0.05; 0.38) |  | **0.07 (<0.01; 0.14)*** |  | 0.03 (-0.10; 0.15) |  | 0.06 (-0.07; 0.19) |  | 0.11 (-0.02; 0.21) |
| Ethnicity, Black/White |  | -0.13 (-0.40; 0.05) |  | **-0.16 (-0.23; -0.08)**** |  | **-0.25 (-0.39; -0.13)**** |  | **-0.19 (-0.34; -0.06)*** |  | -0.02 (-0.16; 0.11) |
| Protein intake, g |  | -0.06 (-0.24; 0.10) |  | -0.02 (-0.08; 0.05) |  | 0.04 (-0.07; 0.16) |  | -0.04 (-0.15; 0.07) |  | -0.04 (-0.13; 0.07) |
| Waist-to-height ratio |  | 0.12 (-0.05; 0.40) |  | **0.17 (0.10; 0.23)**** |  | 0.10 (-0.01; 0.30) |  | 0.06 (-0.07; 0.22) |  | **0.21 (0.05; 0.24)*** |
| Physical act, kCal/kg/day |  | -0.05 (-0.31; 0.16) |  | 0.01 (-0.04; 0.07) |  | 0.05 (-0.06; 0.18) |  | <0.01 (-0.10; 0.10) |  | 0.01 (-0.07; 0.09) |
| Cotinine, ng/ml |  |  |  | **0.08 (0.02; 0.13)*** |  | 0.04 (-0.08; 0.21) |  | 0.07 (-0.04; 0.18) |  | 0.04 (-0.06; 0.12) |
| GGT, U/L |  | **-0.30 (-0.64; -0.17)**** |  | **-0.23 (-0.30; -0.16)**** |  | **-0.32 (-0.49; -0.23)**** |  | **-0.20 (-0.37; -0.11)**** |  | **-0.16 (-0.23; -0.03)*** |
| 24h Systolic BP, mmHg |  | -0.04 (-0.32; 0.20) |  | 0.01 (-0.06; 0.08) |  | 0.03 (-0.10; 0.17) |  | <0.01 (-0.13; 0.13) |  | -0.03 (-0.14; 0.09) |
| HbA1c, (%) |  | 0.01 (-0.20; 0.23) |  | -0.03 (-0.09; 0.03) |  | -0.06 (-0.19; 0.06) |  | -0.07 (-0.19; 0.03) |  | 0.01 (-0.09; 0.11) |
| LDL cholesterol, mmol/L |  | **-0.35 (-0.85; -0.31)**** |  | **-0.25 (-0.30; -0.18)**** |  | **-0.25 (-0.43; -0.17)**** |  | **-0.25 (-0.40; -0.16)**** |  | **-0.30 (-0.31; -0.12)**** |
| SES score |  | 0.06 (-0.21; 0.43) |  | **-0.09 (-0.16; -0.02)*** |  | -0.07 (-0.22; 0.06) |  | -0.05 (-0.19; 0.09) |  | -0.11 (-0.21; 0.03) |
| Glycine, AU | **0.37**** | **0.18 (0.04; 0.37)*** | **0.28**** | **0.10 (0.03; 0.15)*** | **0.34**** | 0.09 (-0.01; 0.19) | **0.30**** | 0.10 (<0.01; 0.20) | **0.19**** | 0.08 (-0.03; 0.17) |
| Age, years |  | **-0.23 (-0.46; -0.07)*** |  | **-0.20 (-0.26; -0.14)**** |  | **-0.22 (-0.33; -0.11)**** |  | **-0.21 (-0.34; -0.10)**** |  | **-0.16 (-0.23; -0.03)*** |
| Sex, female/male |  | 0.18 (-0.01; 0.41) |  | **0.08 (0.01; 0.15)*** |  | 0.03 (-0.09; 0.16) |  | 0.06 (-0.06; 0.19) |  | 0.12 (-0.01; 0.23) |
| Ethnicity, Black/White |  | -0.11 (-0.37; 0.07) |  | **-0.15 (-0.22; -0.07)**** |  | **-0.24 (-0.38; -0.12)**** |  | **-0.18 (-0.33; -0.05)*** |  | -0.02 (-0.16; 0.11) |
| Protein intake, g |  | -0.07 (-0.25; 0.10) |  | -0.01 (-0.07; 0.05) |  | 0.05 (-0.06; 0.16) |  | -0.04 (-0.15; 0.07) |  | -0.04 (-0.13; 0.06) |
| Waist-to-height ratio |  | 0.11 (-0.06; 0.38) |  | **0.16 (0.09; 0.22)**** |  | 0.09 (-0.02; 0.30) |  | 0.05 (-0.08; 0.21) |  | **0.21 (0.05; 0.23)*** |
| Physical act, kCal/kg/day |  | -0.03 (-0.29; 0.18) |  | 0.02 (-0.04; 0.07) |  | 0.05 (-0.06; 0.18) |  | 0.01 (-0.09; 0.11) |  | 0.02 (-0.07; 0.10) |
| Cotinine, ng/ml |  |  |  | **0.07 (0.01; 0.12)*** |  | 0.05 (-0.07; 0.22) |  | 0.06 (-0.05; 0.17) |  | 0.03 (-0.07; 0.11) |
| GGT, U/L |  | **-0.28 (-0.61; -0.15)*** |  | **-0.22 (-0.29; -0.15)**** |  | **-0.31 (-0.48; -0.22)**** |  | **-0.19 (-0.37; -0.10)**** |  | **-0.15 (-0.23; -0.02)*** |
| 24h Systolic BP, mmHg |  | -0.04 (-0.31; 0.20) |  | 0.01 (-0.06; 0.08) |  | 0.04 (-0.09; 0.18) |  | 0.01 (-0.13; 0.14) |  | -0.04 (-0.14; 0.08) |
| HbA1c, (%) |  | 0.02 (-0.18; 0.24) |  | -0.04 (-0.10; 0.03) |  | -0.05 (-0.18; 0.06) |  | -0.07 (-0.19; 0.03) |  | <0.01 (-0.10; 0.10) |
| LDL cholesterol, mmol/L |  | **-0.35 (-0.84; -0.32)**** |  | **-0.25 (-0.30; -0.18)**** |  | **-0.25 (-0.42; -0.16)**** |  | **-0.25 (-0.40; -0.16)**** |  | **-0.31 (-0.32; -0.13)**** |
| SES score |  | 0.06 (-0.19; 0.44) |  | **-0.09 (-0.15; -0.01)*** |  | -0.07 (-0.22; 0.06) |  | -0.04 (-0.18; 0.10) |  | -0.10 (-0.20; 0.03) |
| Serine, AU | **0.34**** | 0.05 (-0.11; 0.23) | **0.28**** | **0.10 (0.04; 0.15)**** | **0.33**** | 0.06 (-0.04; 0.16) | **0.30**** | 0.10 (<0.01; 0.20) | **0.20**** | 0.11 (<0.01; 0.20) |
| Age, years |  | **-0.21 (-0.45; -0.05)*** |  | **-0.20 (-0.26; -0.13)**** |  | **-0.21 (-0.33; -0.11)**** |  | **-0.21 (-0.34; -0.10)**** |  | **-0.15 (-0.23; -0.03)*** |
| Sex, female/male |  | 0.15 (-0.04; 0.39) |  | **0.08 (0.01; 0.15)*** |  | 0.02 (-0.10; 0.15) |  | 0.06 (-0.07; 0.19) |  | 0.12 (-0.01; 0.22) |
| Ethnicity, Black/White |  | -0.13 (-0.39; 0.06) |  | **-0.15 (-0.22; -0.07)**** |  | **-0.25 (-0.38; -0.12)**** |  | **-0.18 (-0.33; -0.06)*** |  | -0.02 (-0.15; 0.12) |
| Protein intake, g |  | -0.06 (-0.24; 0.11) |  | -0.01 (-0.07; 0.05) |  | 0.04 (-0.07; 0.16) |  | -0.04 (-0.15; 0.07) |  | -0.03 (-0.12; 0.07) |
| Waist-to-height ratio |  | 0.12 (-0.05; 0.4) |  | **0.17 (0.10; 0.23)**** |  | 0.10 (<0.01; 0.31) |  | 0.06 (-0.07; 0.22) |  | **0.22 (0.05; 0.24)*** |
| Physical act, kCal/kg/day |  | -0.05 (-0.31; 0.17) |  | 0.01 (-0.04; 0.07) |  | 0.05 (-0.06; 0.18) |  | <0.01 (-0.10; 0.10) |  | 0.01 (-0.07; 0.10) |
| Cotinine, ng/ml |  |  |  | **0.08 (0.02; 0.13)*** |  | 0.05 (-0.08; 0.22) |  | 0.07 (-0.04; 0.17) |  | 0.04 (-0.06; 0.12) |
| GGT, U/L |  | **-0.31 (-0.64; -0.17)**** |  | **-0.22 (-0.29; -0.16)**** |  | **-0.32 (-0.49; -0.23)**** |  | **-0.20 (-0.37; -0.11)**** |  | **-0.15 (-0.23; -0.02)*** |
| 24h Systolic BP, mmHg |  | -0.05 (-0.33; 0.2) |  | 0.01 (-0.06; 0.08) |  | 0.04 (-0.09; 0.18) |  | <0.01 (-0.13; 0.14) |  | -0.04 (-0.14; 0.08) |
| HbA1c, (%) |  | 0.01 (-0.20; 0.23) |  | -0.04 (-0.09; 0.03) |  | -0.06 (-0.19; 0.06) |  | -0.07 (-0.18; 0.04) |  | <0.01 (-0.10; 0.10) |
| LDL cholesterol, mmol/L |  | **-0.35 (-0.85; -0.31)**** |  | **-0.25 (-0.30; -0.18)**** |  | **-0.25 (-0.43; -0.17)**** |  | **-0.25 (-0.40; -0.16)**** |  | **-0.30 (-0.31; -0.12)**** |
| SES score |  | 0.06 (-0.21; 0.43) |  | **-0.09 (-0.15; -0.01)*** |  | -0.07 (-0.22; 0.06) |  | -0.04 (-0.18; 0.10) |  | -0.10 (-0.20; 0.03) |

Test used: Multiple linear regressions. Data are presented as adjusted R^2^ with β coefficient and 95% confidence intervals. Estimated glomerular filtration rate (creatinine-based), adjusted for age, sex, ethnicity, protein intake, waist-to-hight ratio, physical activity, cotinine, GGT, 24h systolic BP, HbA1c, LDL, SES score. Bold values denote P≤0.05; *P≤0.05; **P≤0.001. Cardiovascular disease risk group criteria: Obese - ≥0.55 waist-to-height ratio; Physically inactive - <600 METs for moderate and/or vigorous intensity physical activity; Smoking - ≥11 ng/mL cotinine & self-reported smoking; Excessive alcohol intake - ≥49 U/L GGT & self-reported drinking; Masked hypertensive – normal clinic BP & 24h/day/night BP classified as hypertensive; Hyperglycemic - ≥5.7% HbA1c; Dyslipidemic - >3.4 mmol/L LDL; Low socio-economic – low SES.

*Abbreviations*: AU, arbitrary units; physical act, physical activity; GGT, gamma-glutamyl transferase; BP, blood pressure; HbA1c, glycated haemoglobin; LDL, low density lipoprotein; SES, socio-economic status; eGFR, estimated glomerular filtration rate; CVD, cardiovascular disease.

**Supplementary Table 3C. Supplementary analysis with estimated glomerular filtration rate (creatinine-based) as the dependent variable, with the metabolomics data in control, cardiovascular disease risk group and cardiovascular disease risk clusters**

|  | **eGFR (creatinine), ml/min/1.73m^2^** | | | | | | | | | |
| --- | --- | --- | --- | --- | --- | --- | --- | --- | --- | --- |
|  | **Control group**  **(N=166)** | | **CVD risk group**  **(N=1036)** | | **1 CVD risk factor**  **(N=344)** | | **2 CVD risk factors (N=360)** | | **3+ CVD risk factors (N=332)** | |
| ***Metabolomic data*** | **Adj R^2^** | **β (95%Cl)** | **Adj R^2^** | **β (95%Cl)** | **Adj R^2^** | **β (95%Cl)** | **Adj R^2^** | **β (95%Cl)** | **Adj R^2^** | **β (95%Cl)** |
| Glutamine, AU | **0.35**** | 0.12 (-0.03; 0.30) | **0.29**** | **0.13 (0.07; 0.18)**** | **0.34**** | 0.09 (-0.01; 0.19) | **0.32**** | **0.17 (0.07; 0.27)**** | **0.20**** | 0.11 (<0.01; 0.19) |
| Age, years |  | **-0.22 (-0.45; -0.06)*** |  | **-0.21 (-0.26; -0.14)**** |  | **-0.22 (-0.33; -0.11)**** |  | **-0.21 (-0.34; -0.10)**** |  | **-0.16 (-0.24; -0.03)*** |
| Sex, female/male |  | 0.15 (-0.04; 0.38) |  | **0.07 (<0.01; 0.14)*** |  | 0.02 (-0.10; 0.14) |  | 0.05 (-0.07; 0.18) |  | 0.11 (-0.02; 0.21) |
| Ethnicity, Black/White |  | -0.13 (-0.38; 0.06) |  | **-0.15 (-0.22; -0.07)**** |  | **-0.24 (-0.38; -0.12)**** |  | **-0.18 (-0.33; -0.06)*** |  | -0.02 (-0.15; 0.11) |
| Protein intake, g |  | -0.07 (-0.25; 0.10) |  | -0.01 (-0.07; 0.05) |  | 0.05 (-0.06; 0.16) |  | -0.04 (-0.15; 0.06) |  | -0.04 (-0.13; 0.07) |
| Waist-to-height ratio |  | 0.12 (-0.05; 0.40) |  | **0.17 (0.10; 0.23)**** |  | 0.10 (-0.01; 0.30) |  | 0.06 (-0.06; 0.22) |  | **0.22 (0.05; 0.24)*** |
| Physical act, kCal/kg/day |  | -0.04 (-0.30; 0.17) |  | 0.01 (-0.04; 0.07) |  | 0.04 (-0.07; 0.17) |  | <0.01 (-0.09; 0.10) |  | 0.01 (-0.07; 0.10) |
| Cotinine, ng/ml |  |  |  | **0.08 (0.02; 0.13)*** |  | 0.05 (-0.07; 0.22) |  | 0.07 (-0.04; 0.17) |  | 0.04 (-0.06; 0.12) |
| GGT, U/L |  | **-0.30 (-0.63; -0.16)**** |  | **-0.22 (-0.29; -0.15)**** |  | **-0.32 (-0.49; -0.23)**** |  | **-0.19 (-0.37; -0.11)**** |  | **-0.15 (-0.23; -0.02)*** |
| 24h Systolic BP, mmHg |  | -0.05 (-0.34; 0.19) |  | 0.01 (-0.06; 0.08) |  | 0.04 (-0.09; 0.18) |  | <0.01 (-0.13; 0.13) |  | -0.04 (-0.14; 0.08) |
| HbA1c, (%) |  | 0.01 (-0.19; 0.23) |  | -0.03 (-0.09; 0.03) |  | -0.06 (-0.19; 0.06) |  | -0.07 (-0.18; 0.03) |  | 0.01 (-0.09; 0.11) |
| LDL cholesterol, mmol/L |  | **-0.34 (-0.83; -0.30)**** |  | **-0.25 (-0.30; -0.17)**** |  | **-0.24 (-0.42; -0.16)**** |  | **-0.24 (-0.38; -0.15)**** |  | **-0.30 (-0.31; -0.12)**** |
| SES score |  | 0.06 (-0.20; 0.43) |  | **-0.08 (-0.15; -0.01)*** |  | -0.07 (-0.23; 0.06) |  | -0.03 (-0.17; 0.11) |  | -0.10 (-0.20; 0.03) |
| Isothreonine, AU | -0.41 | -0.02 (-0.92; 0.86) | **0.24**** | -0.09 (-0.22; 0.04) | **0.27*** | **-0.33 (-0.54; -0.06)*** | **0.16*** | 0.06 (-0.22; 0.36) | 0.02 | -0.07 (-0.32; 0.21) |
| Age, years |  | -0.21 (-1.11; 0.62) |  | **-0.21 (-0.34; -0.06)*** |  | -0.24 (-0.52; 0.02) |  | -0.19 (-0.50; 0.09) |  | -0.15 (-0.37; 0.13) |
| Sex, female/male |  | 0.13 (-0.79; 1.09) |  | 0.07 (-0.09; 0.22) |  | -0.07 (-0.38; 0.23) |  | 0.04 (-0.26; 0.35) |  | 0.11 (-0.18; 0.38) |
| Ethnicity, Black/White |  | -0.13 (-1.11; 0.76) |  | **-0.18 (-0.35; -0.01)*** |  | -0.30 (-0.63; 0.01) |  | -0.18 (-0.53; 0.14) |  | -0.06 (-0.38; 0.28) |
| Protein intake, g |  | -0.06 (-0.81; 0.68) |  | -0.03 (-0.16; 0.11) |  | 0.08 (-0.19; 0.35) |  | -0.04 (-0.30; 0.22) |  | -0.06 (-0.28; 0.18) |
| Waist-to-height ratio |  | 0.11 (-0.82; 1.14) |  | **0.16 (0.01; 0.30)*** |  | 0.12 (-0.19; 0.55) |  | 0.05 (-0.29; 0.41) |  | 0.19 (-0.09; 0.35) |
| Physical act, kCal/kg/day |  | -0.04 (-1.07; 0.93) |  | 0.02 (-0.10; 0.15) |  | 0.11 (-0.16; 0.43) |  | <0.01 (-0.24; 0.23) |  | 0.02 (-0.19; 0.22) |
| Cotinine, ng/ml |  |  |  | 0.06 (-0.07; 0.18) |  | -0.03 (-0.40; 0.32) |  | 0.06 (-0.19; 0.32) |  | 0.02 (-0.20; 0.23) |
| GGT, U/L |  | -0.32 (-1.47; 0.61) |  | **-0.25 (-0.41; -0.10)**** |  | **-0.28 (-0.63; -0.01)*** |  | -0.20 (-0.56; 0.07) |  | -0.19 (-0.42; 0.10) |
| 24h Systolic BP, mmHg |  | -0.03 (-1.17; 1.07) |  | 0.03 (-0.13; 0.18) |  | -0.01 (-0.34; 0.32) |  | 0.02 (-0.30; 0.34) |  | <0.01 (-0.28; 0.27) |
| HbA1c, (%) |  | 0.01 (-0.88; 0.91) |  | -0.05 (-0.18; 0.09) |  | -0.10 (-0.41; 0.18) |  | -0.07 (-0.33; 0.19) |  | -0.01 (-0.25; 0.23) |
| LDL cholesterol, mmol/L |  | -0.35 (-1.71; 0.54) |  | **-0.26 (-0.38; -0.11)**** |  | **-0.35 (-0.73; -0.10)*** |  | -0.26 (-0.58; <0.01) |  | -0.30 (-0.45; 0.01) |
| SES score |  | 0.05 (-1.37; 1.56) |  | -0.08 (-0.23; 0.08) |  | 0.09 (-0.27; 0.48) |  | -0.06 (-0.40; 0.27) |  | -0.09 (-0.35; 0.21) |
| Dimethylglycine, AU | **0.33**** | 0.01 (-0.16; 0.19) | **0.28**** | 0.05 (<0.01; 0.11) | **0.33**** | 0.06 (-0.04; 0.18) | **0.29**** | 0.03 (-0.07; 0.13) | **0.18**** | 0.04 (-0.06; 0.13) |
| Age, years |  | **-0.22 (-0.45; -0.05)*** |  | **-0.21 (-0.27; -0.14)**** |  | **-0.22 (-0.33; -0.11)**** |  | **-0.21 (-0.35; -0.10)**** |  | **-0.16 (-0.24; -0.03)*** |
| Sex, female/male |  | 0.14 (-0.05; 0.38) |  | **0.07 (<0.01; 0.14)*** |  | 0.02 (-0.10; 0.15) |  | 0.05 (-0.08; 0.18) |  | 0.11 (-0.03; 0.21) |
| Ethnicity, Black/White |  | -0.13 (-0.40; 0.05) |  | **-0.16 (-0.23; -0.08)**** |  | **-0.25 (-0.39; -0.12)**** |  | **-0.18 (-0.34; -0.06)*** |  | -0.04 (-0.17; 0.10) |
| Protein intake, g |  | -0.06 (-0.25; 0.11) |  | -0.02 (-0.08; 0.04) |  | 0.05 (-0.07; 0.16) |  | -0.04 (-0.15; 0.07) |  | -0.05 (-0.14; 0.06) |
| Waist-to-height ratio |  | 0.11 (-0.06; 0.40) |  | **0.17 (0.09; 0.23)**** |  | **0.10 (<0.01; 0.31)*** |  | 0.05 (-0.09; 0.21) |  | **0.21 (0.04; 0.23)*** |
| Physical act, kCal/kg/day |  | -0.04 (-0.31; 0.18) |  | 0.01 (-0.04; 0.07) |  | 0.05 (-0.06; 0.19) |  | <0.01 (-0.10; 0.10) |  | 0.01 (-0.08; 0.09) |
| Cotinine, ng/ml |  |  |  | **0.07 (0.01; 0.12)*** |  | 0.04 (-0.08; 0.21) |  | 0.06 (-0.04; 0.17) |  | 0.04 (-0.06; 0.12) |
| GGT, U/L |  | **-0.31 (-0.65; -0.18)**** |  | **-0.23 (-0.30; -0.17)**** |  | **-0.32 (-0.49; -0.23)**** |  | **-0.20 (-0.38; -0.12)**** |  | **-0.16 (-0.24; -0.03)*** |
| 24h Systolic BP, mmHg |  | -0.04 (-0.33; 0.21) |  | 0.02 (-0.06; 0.09) |  | 0.03 (-0.11; 0.17) |  | 0.02 (-0.11; 0.15) |  | -0.02 (-0.13; 0.10) |
| HbA1c, (%) |  | 0.01 (-0.20; 0.23) |  | -0.03 (-0.10; 0.03) |  | -0.05 (-0.18; 0.06) |  | -0.07 (-0.18; 0.04) |  | <0.01 (-0.10; 0.10) |
| LDL cholesterol, mmol/L |  | **-0.35 (-0.86; -0.31)**** |  | **-0.26 (-0.31; -0.18)**** |  | **-0.26 (-0.43; -0.17)**** |  | **-0.26 (-0.40; -0.16)**** |  | **-0.30 (-0.32; -0.12)**** |
| SES score |  | 0.06 (-0.21; 0.44) |  | **-0.09 (-0.16; -0.02)*** |  | -0.07 (-0.22; 0.07) |  | -0.06 (-0.21; 0.08) |  | -0.10 (-0.20; 0.04) |
| Beta-alanine, AU | **0.34**** | 0.03 (-0.14; 0.22) | **0.28**** | 0.01 (-0.04; 0.07) | **0.33**** | 0.02 (-0.08; 0.12) | **0.30**** | 0.04 (-0.06; 0.15) | **0.18**** | <0.01 (-0.09; 0.09) |
| Age, years |  | **-0.23 (-0.47; -0.06)*** |  | **-0.20 (-0.26; -0.13)**** |  | **-0.22 (-0.33; -0.11)**** |  | **-0.21 (-0.34; -0.10)**** |  | **-0.15 (-0.23; -0.02)*** |
| Sex, female/male |  | 0.14 (-0.05; 0.37) |  | **0.07 (<0.01; 0.14)*** |  | 0.03 (-0.10; 0.15) |  | 0.05 (-0.08; 0.18) |  | 0.11 (-0.02; 0.22) |
| Ethnicity, Black/White |  | -0.13 (-0.40; 0.05) |  | **-0.16 (-0.23; -0.08)**** |  | **-0.25 (-0.39; -0.13)**** |  | **-0.19 (-0.34; -0.06)*** |  | -0.03 (-0.17; 0.11) |
| Protein intake, g |  | -0.07 (-0.26; 0.10) |  | -0.02 (-0.08; 0.04) |  | 0.04 (-0.07; 0.15) |  | -0.04 (-0.16; 0.06) |  | -0.05 (-0.14; 0.06) |
| Waist-to-height ratio |  | 0.11 (-0.06; 0.39) |  | **0.16 (0.09; 0.22)**** |  | 0.10 (<0.01; 0.31) |  | 0.05 (-0.08; 0.21) |  | **0.20 (0.04; 0.23)*** |
| Physical act, kCal/kg/day |  | -0.04 (-0.30; 0.17) |  | 0.01 (-0.04; 0.07) |  | 0.05 (-0.06; 0.18) |  | <0.01 (-0.10; 0.10) |  | 0.01 (-0.08; 0.09) |
| Cotinine, ng/ml |  |  |  | **0.07 (0.01; 0.12)*** |  | 0.04 (-0.09; 0.21) |  | 0.06 (-0.04; 0.17) |  | 0.03 (-0.07; 0.11) |
| GGT, U/L |  | **-0.31 (-0.65; -0.18)**** |  | **-0.24 (-0.31; -0.17)**** |  | **-0.33 (-0.50; -0.24)**** |  | **-0.21 (-0.38; -0.12)**** |  | **-0.17 (-0.24; -0.04)*** |
| 24h Systolic BP, mmHg |  | -0.04 (-0.32; 0.21) |  | 0.02 (-0.05; 0.09) |  | 0.03 (-0.10; 0.17) |  | 0.01 (-0.12; 0.15) |  | -0.02 (-0.13; 0.09) |
| HbA1c, (%) |  | <0.01 (-0.21; 0.22) |  | -0.03 (-0.09; 0.03) |  | -0.05 (-0.18; 0.06) |  | -0.07 (-0.18; 0.03) |  | 0.01 (-0.10; 0.10) |
| LDL cholesterol, mmol/L |  | **-0.35 (-0.85; -0.32)**** |  | **-0.26 (-0.31; -0.19)**** |  | **-0.26 (-0.44; -0.18)**** |  | **-0.26 (-0.40; -0.16)**** |  | **-0.31 (-0.32; -0.13)**** |
| SES score |  | 0.07 (-0.20; 0.45) |  | **-0.09 (-0.16; -0.02)*** |  | -0.06 (-0.22; 0.07) |  | -0.05 (-0.20; 0.09) |  | -0.09 (-0.20; 0.04) |

Test used: Multiple linear regressions. Data are presented as adjusted R^2^ with β coefficient and 95% confidence intervals. Estimated glomerular filtration rate (creatinine-based), adjusted for age, sex, ethnicity, protein intake, waist-to-hight ratio, physical activity, cotinine, GGT, 24h systolic BP, HbA1c, LDL, SES score. Bold values denote P≤0.05; *P≤0.05; **P≤0.001. Cardiovascular disease risk group criteria: Obese - ≥0.55 waist-to-height ratio; Physically inactive - <600 METs for moderate and/or vigorous intensity physical activity; Smoking - ≥11 ng/mL cotinine & self-reported smoking; Excessive alcohol intake - ≥49 U/L GGT & self-reported drinking; Masked hypertensive – normal clinic BP & 24h/day/night BP classified as hypertensive; Hyperglycemic - ≥5.7% HbA1c; Dyslipidemic - >3.4 mmol/L LDL; Low socio-economic – low SES.

*Abbreviations*: AU, arbitrary units; physical act, physical activity; GGT, gamma-glutamyl transferase; BP, blood pressure; HbA1c, glycated haemoglobin; LDL, low density lipoprotein; SES, socio-economic status; eGFR, estimated glomerular filtration rate; CVD, cardiovascular disease.

**Supplementary Table 3D. Supplementary analysis with estimated glomerular filtration rate (creatinine-based) as the dependent variable, with the metabolomics data in control, cardiovascular disease risk group and cardiovascular disease risk clusters**

|  | **eGFR (creatinine), ml/min/1.73m^2^** | | | | | | | | | |
| --- | --- | --- | --- | --- | --- | --- | --- | --- | --- | --- |
|  | **Control group**  **(N=166)** | | **CVD risk group**  **(N=1036)** | | **1 CVD risk factor**  **(N=344)** | | **2 CVD risk factors (N=360)** | | **3+ CVD risk factors (N=332)** | |
| ***Metabolomic data*** | **Adj R^2^** | **β (95%Cl)** | **Adj R^2^** | **β (95%Cl)** | **Adj R^2^** | **β (95%Cl)** | **Adj R^2^** | **β (95%Cl)** | **Adj R^2^** | **β (95%Cl)** |
| Threonine, AU | **0.34**** | 0.06 (-0.10; 0.24) | **0.28**** | **0.09 (0.03; 0.15)*** | **0.33**** | 0.04 (-0.06; 0.14) | **0.31**** | **0.12 (0.02; 0.22)*** | **0.19**** | 0.10 (-0.01; 0.19) |
| Age, years |  | **-0.22 (-0.45; -0.06)*** |  | **-0.20 (-0.26; -0.13)**** |  | **-0.21 (-0.33; -0.11)**** |  | **-0.21 (-0.34; -0.10)**** |  | **-0.15 (-0.23; -0.03)*** |
| Sex, female/male |  | 0.15 (-0.04; 0.39) |  | **0.08 (0.01; 0.15)*** |  | 0.03 (-0.10; 0.15) |  | 0.06 (-0.06; 0.19) |  | 0.12 (-0.01; 0.22) |
| Ethnicity, Black/White |  | -0.13 (-0.39; 0.05) |  | **-0.15 (-0.23; -0.08)**** |  | **-0.25 (-0.39; -0.13)**** |  | **-0.19 (-0.34; -0.06)*** |  | -0.03 (-0.16; 0.11) |
| Protein intake, g |  | -0.06 (-0.25; 0.10) |  | -0.02 (-0.08; 0.04) |  | 0.04 (-0.07; 0.15) |  | -0.04 (-0.15; 0.06) |  | -0.04 (-0.13; 0.07) |
| Waist-to-height ratio |  | 0.12 (-0.05; 0.40) |  | **0.17 (0.10; 0.23)**** |  | 0.10 (-0.01; 0.31) |  | 0.06 (-0.07; 0.22) |  | **0.22 (0.05; 0.24)*** |
| Physical act, kCal/kg/day |  | -0.05 (-0.31; 0.16) |  | 0.01 (-0.04; 0.07) |  | 0.05 (-0.06; 0.18) |  | <0.01 (-0.10; 0.10) |  | 0.01 (-0.07; 0.09) |
| Cotinine, ng/ml |  |  |  | **0.08 (0.02; 0.13)*** |  | 0.05 (-0.08; 0.22) |  | 0.07 (-0.04; 0.17) |  | 0.04 (-0.06; 0.12) |
| GGT, U/L |  | **-0.30 (-0.64; -0.17)**** |  | **-0.22 (-0.29; -0.16)**** |  | **-0.32 (-0.49; -0.23)**** |  | **-0.19 (-0.37; -0.11)**** |  | **-0.15 (-0.23; -0.02)*** |
| 24h Systolic BP, mmHg |  | -0.05 (-0.33; 0.20) |  | 0.01 (-0.06; 0.08) |  | 0.04 (-0.10; 0.17) |  | <0.01 (-0.14; 0.13) |  | -0.04 (-0.14; 0.08) |
| HbA1c, (%) |  | 0.01 (-0.20; 0.23) |  | -0.04 (-0.10; 0.03) |  | -0.06 (-0.19; 0.06) |  | -0.07 (-0.18; 0.03) |  | 0.01 (-0.10; 0.10) |
| LDL cholesterol, mmol/L |  | **-0.35 (-0.85; -0.31)**** |  | **-0.25 (-0.30; -0.18)**** |  | **-0.25 (-0.43; -0.17)**** |  | **-0.25 (-0.39; -0.15)**** |  | **-0.30 (-0.31; -0.12)**** |
| SES score |  | 0.07 (-0.20; 0.44) |  | **-0.09 (-0.16; -0.02)*** |  | -0.07 (-0.22; 0.06) |  | -0.04 (-0.19; 0.10) |  | -0.10 (-0.21; 0.03) |
| Hydroxyproline, AU | -0.22 | -0.41 (-1.49; 0.46) | **0.24**** | -0.03 (-0.15; 0.09) | **0.40**** | **-0.54 (-0.78; -0.27)**** | **0.19*** | 0.09 (-0.14; 0.34) | 0.06 | 0.13 (-0.12; 0.34) |
| Age, years |  | -0.15 (-0.96; 0.61) |  | **-0.20 (-0.33; -0.06)*** |  | **-0.29 (-0.54; -0.05)*** |  | -0.22 (-0.50; 0.03) |  | -0.19 (-0.39; 0.08) |
| Sex, female/male |  | -0.07 (-1.02; 0.87) |  | 0.07 (-0.08; 0.22) |  | 0.02 (-0.24; 0.29) |  | 0.05 (-0.23; 0.33) |  | 0.13 (-0.15; 0.37) |
| Ethnicity, Black/White |  | -0.09 (-0.99; 0.76) |  | **-0.17 (-0.33; <0.01)*** |  | **-0.31 (-0.60; -0.03)*** |  | -0.16 (-0.48; 0.15) |  | 0.01 (-0.29; 0.31) |
| Protein intake, g |  | 0.01 (-0.69; 0.71) |  | -0.02 (-0.15; 0.11) |  | -0.05 (-0.30; 0.19) |  | -0.04 (-0.28; 0.20) |  | -0.06 (-0.26; 0.16) |
| Waist-to-height ratio |  | 0.11 (-0.71; 1.04) |  | **0.17 (0.02; 0.30)*** |  | **0.35 (0.14; 0.90)*** |  | 0.03 (-0.27; 0.36) |  | 0.22 (-0.06; 0.35) |
| Physical act, kCal/kg/day |  | 0.07 (-0.87; 1.11) |  | 0.02 (-0.10; 0.13) |  | 0.20 (-0.03; 0.52) |  | <0.01 (-0.21; 0.22) |  | -0.01 (-0.19; 0.18) |
| Cotinine, ng/ml |  |  |  | 0.07 (-0.05; 0.19) |  | -0.10 (-0.48; 0.18) |  | 0.05 (-0.19; 0.29) |  | 0.03 (-0.17; 0.22) |
| GGT, U/L |  | -0.11 (-1.19; 0.9) |  | **-0.25 (-0.39; -0.10)**** |  | **-0.46 (-0.80; -0.23)**** |  | -0.18 (-0.52; 0.07) |  | -0.13 (-0.34; 0.12) |
| 24h Systolic BP, mmHg |  | 0.02 (-1.01; 1.06) |  | 0.02 (-0.13; 0.17) |  | -0.04 (-0.34; 0.25) |  | 0.02 (-0.27; 0.30) |  | -0.08 (-0.32; 0.20) |
| HbA1c, (%) |  | -0.10 (-1.03; 0.74) |  | -0.04 (-0.17; 0.09) |  | -0.09 (-0.36; 0.16) |  | -0.07 (-0.31; 0.16) |  | 0.03 (-0.20; 0.24) |
| LDL cholesterol, mmol/L |  | -0.41 (-1.73; 0.37) |  | **-0.26 (-0.38; -0.11)**** |  | **-0.24 (-0.56; -0.01)*** |  | **-0.26 (-0.55; -0.02)*** |  | -0.34 (-0.45; -0.03)* |
| SES score |  | -0.10 (-1.56; 1.19) |  | -0.09 (-0.24; 0.06) |  | -0.02 (-0.33; 0.29) |  | -0.06 (-0.37; 0.24) |  | -0.11 (-0.35; 0.16) |
| Alanine, AU | **0.35**** | 0.11 (-0.03; 0.29) | **0.29**** | **0.13 (0.07; 0.19)**** | **0.34**** | 0.09 (-0.01; 0.19) | **0.32**** | **0.17 (0.07; 0.27)**** | **0.20**** | **0.11 (<0.01; 0.20)*** |
| Age, years |  | **-0.21 (-0.45; -0.05)*** |  | **-0.20 (-0.26; -0.14)**** |  | **-0.21 (-0.33; -0.11)**** |  | **-0.20 (-0.34; -0.10)**** |  | **-0.16 (-0.24; -0.03)*** |
| Sex, female/male |  | 0.15 (-0.04; 0.38) |  | 0.07 (<0.01; 0.13) |  | 0.02 (-0.11; 0.14) |  | 0.05 (-0.07; 0.18) |  | 0.12 (-0.02; 0.22) |
| Ethnicity, Black/White |  | -0.13 (-0.39; 0.05) |  | **-0.15 (-0.22; -0.07)**** |  | **-0.25 (-0.39; -0.12)**** |  | **-0.18 (-0.33; -0.06)*** |  | -0.02 (-0.15; 0.11) |
| Protein intake, g |  | -0.06 (-0.24; 0.10) |  | -0.01 (-0.07; 0.05) |  | 0.04 (-0.07; 0.16) |  | -0.04 (-0.15; 0.07) |  | -0.03 (-0.12; 0.07) |
| Waist-to-height ratio |  | 0.12 (-0.04; 0.41) |  | **0.16 (0.09; 0.22)**** |  | 0.09 (-0.01; 0.30) |  | 0.06 (-0.07; 0.22) |  | **0.21 (0.05; 0.24)*** |
| Physical act, kCal/kg/day |  | -0.05 (-0.31; 0.16) |  | 0.01 (-0.04; 0.07) |  | 0.05 (-0.06; 0.18) |  | <0.01 (-0.10; 0.10) |  | 0.01 (-0.07; 0.10) |
| Cotinine, ng/ml |  |  |  | **0.08 (0.02; 0.13)*** |  | 0.05 (-0.07; 0.23) |  | 0.06 (-0.04; 0.17) |  | 0.04 (-0.06; 0.12) |
| GGT, U/L |  | **-0.30 (-0.64; -0.17)**** |  | **-0.22 (-0.29; -0.15)**** |  | **-0.31 (-0.48; -0.22)**** |  | **-0.19 (-0.36; -0.11)**** |  | **-0.15 (-0.23; -0.02)*** |
| 24h Systolic BP, mmHg |  | -0.06 (-0.35; 0.18) |  | <0.01 (-0.07; 0.07) |  | 0.04 (-0.09; 0.18) |  | -0.01 (-0.14; 0.12) |  | -0.04 (-0.15; 0.08) |
| HbA1c, (%) |  | 0.01 (-0.20; 0.23) |  | -0.04 (-0.10; 0.03) |  | -0.06 (-0.19; 0.05) |  | -0.07 (-0.18; 0.03) |  | 0.01 (-0.09; 0.11) |
| LDL cholesterol, mmol/L |  | **-0.34 (-0.83; -0.30)**** |  | **-0.25 (-0.30; -0.17)**** |  | **-0.24 (-0.42; -0.16)**** |  | **-0.24 (-0.38; -0.14)**** |  | **-0.30 (-0.31; -0.12)**** |
| SES score |  | 0.06 (-0.20; 0.44) |  | **-0.08 (-0.15; -0.01)*** |  | -0.07 (-0.22; 0.06) |  | -0.04 (-0.18; 0.10) |  | -0.10 (-0.20; 0.03) |
| Citrulline, AU | **0.32**** | 0.05 (-0.13; 0.23) | **0.27**** | 0.02 (-0.04; 0.09) | **0.32**** | -0.06 (-0.18; 0.06) | **0.29**** | 0.05 (-0.06; 0.16) | **0.18**** | 0.06 (-0.06; 0.16) |
| Age, years |  | **-0.22 (-0.48; -0.03)*** |  | **-0.21 (-0.27; -0.13)**** |  | **-0.21 (-0.34; -0.09)**** |  | **-0.21 (-0.36; -0.09)**** |  | **-0.16 (-0.25; -0.02)*** |
| Sex, female/male |  | 0.14 (-0.07; 0.40) |  | 0.07 (<0.01; 0.15) |  | 0.03 (-0.11; 0.17) |  | 0.05 (-0.09; 0.20) |  | 0.11 (-0.03; 0.23) |
| Ethnicity, Black/White |  | -0.14 (-0.42; 0.07) |  | **-0.16 (-0.24; -0.07)**** |  | **-0.26 (-0.41; -0.12)**** |  | **-0.19 (-0.35; -0.04)*** |  | -0.03 (-0.17; 0.13) |
| Protein intake, g |  | -0.06 (-0.27; 0.12) |  | -0.02 (-0.09; 0.05) |  | 0.04 (-0.09; 0.16) |  | -0.04 (-0.17; 0.08) |  | -0.04 (-0.14; 0.07) |
| Waist-to-height ratio |  | 0.12 (-0.08; 0.42) |  | **0.17 (0.09; 0.23)**** |  | 0.11 (-0.01; 0.34) |  | 0.05 (-0.10; 0.23) |  | **0.21 (0.03; 0.24)*** |
| Physical act, kCal/kg/day |  | -0.04 (-0.33; 0.20) |  | 0.01 (-0.05; 0.07) |  | 0.06 (-0.07; 0.21) |  | <0.01 (-0.11; 0.11) |  | 0.01 (-0.08; 0.10) |
| Cotinine, ng/ml |  |  |  | **0.07 (0.01; 0.13)*** |  | 0.03 (-0.11; 0.22) |  | 0.06 (-0.06; 0.19) |  | 0.03 (-0.07; 0.12) |
| GGT, U/L |  | **-0.31 (-0.67; -0.15)*** |  | **-0.24 (-0.31; -0.16)**** |  | **-0.33 (-0.52; -0.23)**** |  | **-0.20 (-0.39; -0.10)**** |  | **-0.16 (-0.25; -0.02)*** |
| 24h Systolic BP, mmHg |  | -0.04 (-0.35; 0.24) |  | 0.02 (-0.06; 0.09) |  | 0.03 (-0.12; 0.18) |  | 0.01 (-0.13; 0.16) |  | -0.04 (-0.15; 0.10) |
| HbA1c, (%) |  | 0.01 (-0.22; 0.25) |  | -0.03 (-0.10; 0.04) |  | -0.06 (-0.20; 0.07) |  | -0.07 (-0.20; 0.05) |  | 0.01 (-0.10; 0.12) |
| LDL cholesterol, mmol/L |  | **-0.35 (-0.88; -0.28)**** |  | **-0.26 (-0.32; -0.18)**** |  | **-0.26 (-0.46; -0.17)**** |  | **-0.26 (-0.42; -0.14)**** |  | **-0.31 (-0.33; -0.12)**** |
| SES score |  | 0.06 (-0.24; 0.48) |  | **-0.09 (-0.17; -0.01)*** |  | -0.06 (-0.23; 0.09) |  | -0.05 (-0.21; 0.11) |  | -0.09 (-0.21; 0.05) |

Test used: Multiple linear regressions. Data are presented as adjusted R^2^ with β coefficient and 95% confidence intervals. Estimated glomerular filtration rate (creatinine-based), adjusted for age, sex, ethnicity, protein intake, waist-to-hight ratio, physical activity, cotinine, GGT, 24h systolic BP, HbA1c, LDL, SES score. Bold values denote P≤0.05; *P≤0.05; **P≤0.001. Cardiovascular disease risk group criteria: Obese - ≥0.55 waist-to-height ratio; Physically inactive - <600 METs for moderate and/or vigorous intensity physical activity; Smoking - ≥11 ng/mL cotinine & self-reported smoking; Excessive alcohol intake - ≥49 U/L GGT & self-reported drinking; Masked hypertensive – normal clinic BP & 24h/day/night BP classified as hypertensive; Hyperglycemic - ≥5.7% HbA1c; Dyslipidemic - >3.4 mmol/L LDL; Low socio-economic – low SES.

*Abbreviations*: AU, arbitrary units; physical act, physical activity; GGT, gamma-glutamyl transferase; BP, blood pressure; HbA1c, glycated haemoglobin; LDL, low density lipoprotein; SES, socio-economic status; eGFR, estimated glomerular filtration rate; CVD, cardiovascular disease.

**Supplementary Table 3E. Supplementary analysis with estimated glomerular filtration rate (creatinine-based) as the dependent variable, with the metabolomics data in control, cardiovascular disease risk group and cardiovascular disease risk clusters**

|  | **eGFR (creatinine), ml/min/1.73m^2^** | | | | | | | | | |
| --- | --- | --- | --- | --- | --- | --- | --- | --- | --- | --- |
|  | **Control group**  **(N=166)** | | **CVD risk group**  **(N=1036)** | | **1 CVD risk factor**  **(N=344)** | | **2 CVD risk factors (N=360)** | | **3+ CVD risk factors (N=332)** | |
| ***Metabolomic data*** | **Adj R^2^** | **β (95%Cl)** | **Adj R^2^** | **β (95%Cl)** | **Adj R^2^** | **β (95%Cl)** | **Adj R^2^** | **β (95%Cl)** | **Adj R^2^** | **β (95%Cl)** |
| GABA, AU | **0.33**** | 0.07 (-0.11; 0.27) | **0.29**** | **0.11 (0.05; 0.17)**** | **0.34**** | **0.11 (0.01; 0.22)*** | **0.32**** | **0.18 (0.09; 0.36)**** | **0.18**** | 0.01 (-0.08; 0.10) |
| Age, years |  | **-0.22 (-0.47; -0.05)*** |  | **-0.21 (-0.27; -0.14)**** |  | **-0.22 (-0.34; -0.10)**** |  | **-0.22 (-0.37; -0.11)**** |  | **-0.15 (-0.24; -0.02)*** |
| Sex, female/male |  | 0.15 (-0.06; 0.41) |  | **0.09 (0.02; 0.16)*** |  | 0.05 (-0.08; 0.18) |  | 0.08 (-0.05; 0.22) |  | 0.11 (-0.03; 0.22) |
| Ethnicity, Black/White |  | -0.15 (-0.43; 0.05) |  | **-0.18 (-0.25; -0.09)**** |  | **-0.27 (-0.42; -0.14)**** |  | **-0.22 (-0.38; -0.08)*** |  | -0.04 (-0.17; 0.11) |
| Protein intake, g |  | -0.06 (-0.25; 0.12) |  | -0.01 (-0.07; 0.06) |  | 0.06 (-0.06; 0.18) |  | -0.01 (-0.13; 0.11) |  | -0.05 (-0.14; 0.06) |
| Waist-to-height ratio |  | 0.12 (-0.06; 0.42) |  | **0.17 (0.10; 0.23)**** |  | 0.09 (-0.02; 0.31) |  | 0.07 (-0.06; 0.25) |  | **0.20 (0.04; 0.24)*** |
| Physical act, kCal/kg/day |  | -0.04 (-0.31; 0.20) |  | 0.01 (-0.05; 0.06) |  | 0.04 (-0.08; 0.18) |  | <0.01 (-0.10; 0.10) |  | 0.01 (-0.08; 0.10) |
| Cotinine, ng/ml |  |  |  | **0.07 (<0.01; 0.12)*** |  | 0.05 (-0.08; 0.23) |  | 0.05 (-0.06; 0.17) |  | 0.03 (-0.07; 0.12) |
| GGT, U/L |  | **-0.31 (-0.67; -0.17)**** |  | **-0.23 (-0.30; -0.16)**** |  | **-0.32 (-0.49; -0.22)**** |  | **-0.19 (-0.37; -0.10)**** |  | **-0.17 (-0.25; -0.03)*** |
| 24h Systolic BP, mmHg |  | -0.04 (-0.33; 0.23) |  | 0.02 (-0.06; 0.09) |  | 0.05 (-0.09; 0.19) |  | 0.01 (-0.13; 0.15) |  | -0.02 (-0.14; 0.10) |
| HbA1c, (%) |  | 0.02 (-0.20; 0.26) |  | -0.03 (-0.09; 0.04) |  | -0.05 (-0.19; 0.07) |  | -0.06 (-0.18; 0.05) |  | 0.01 (-0.10; 0.11) |
| LDL cholesterol, mmol/L |  | **-0.34 (-0.85; -0.26)**** |  | **-0.25 (-0.31; -0.18)**** |  | **-0.24 (-0.42; -0.15)**** |  | **-0.25 (-0.39; -0.14)**** |  | **-0.31 (-0.33; -0.13)**** |
| SES score |  | 0.06 (-0.22; 0.46) |  | **-0.09 (-0.16; -0.01)*** |  | -0.07 (-0.23; 0.07) |  | -0.03 (-0.18; 0.11) |  | -0.09 (-0.20; 0.04) |
| Creatine, AU | **0.34**** | 0.07 (-0.09; 0.27) | **0.28**** | **0.10 (0.04; 0.16)**** | **0.34**** | 0.08 (-0.02; 0.19) | **0.32**** | **0.18 (0.09; 0.32)**** | **0.19**** | 0.04 (-0.07; 0.13) |
| Age, years |  | **-0.22 (-0.46; -0.06)*** |  | **-0.20 (-0.26; -0.14)**** |  | **-0.21 (-0.33; -0.11)**** |  | **-0.21 (-0.34; -0.11)**** |  | **-0.15 (-0.23; -0.02)*** |
| Sex, female/male |  | 0.16 (-0.03; 0.41) |  | **0.12 (0.04; 0.19)*** |  | 0.05 (-0.08; 0.17) |  | **0.13 (0.01; 0.28)*** |  | 0.13 (-0.02; 0.24) |
| Ethnicity, Black/White |  | -0.12 (-0.38; 0.07) |  | **-0.15 (-0.23; -0.08)**** |  | **-0.25 (-0.39; -0.12)**** |  | **-0.21 (-0.36; -0.08)**** |  | -0.03 (-0.16; 0.11) |
| Protein intake, g |  | -0.08 (-0.26; 0.09) |  | -0.03 (-0.09; 0.03) |  | 0.04 (-0.07; 0.15) |  | -0.06 (-0.17; 0.05) |  | -0.05 (-0.14; 0.05) |
| Waist-to-height ratio |  | 0.11 (-0.05; 0.39) |  | **0.16 (0.09; 0.22)**** |  | 0.10 (-0.01; 0.30) |  | 0.05 (-0.08; 0.21) |  | **0.20 (0.04; 0.23)*** |
| Physical act, kCal/kg/day |  | -0.05 (-0.32; 0.16) |  | 0.01 (-0.05; 0.06) |  | 0.04 (-0.07; 0.17) |  | <0.01 (-0.09; 0.10) |  | 0.01 (-0.08; 0.09) |
| Cotinine, ng/ml |  |  |  | **0.07 (0.01; 0.13)*** |  | 0.05 (-0.07; 0.22) |  | 0.06 (-0.04; 0.17) |  | 0.03 (-0.07; 0.11) |
| GGT, U/L |  | **-0.31 (-0.65; -0.18)**** |  | **-0.24 (-0.31; -0.17)**** |  | **-0.33 (-0.50; -0.24)**** |  | **-0.22 (-0.40; -0.14)**** |  | **-0.16 (-0.24; -0.04)*** |
| 24h Systolic BP, mmHg |  | -0.05 (-0.33; 0.19) |  | 0.01 (-0.06; 0.08) |  | 0.03 (-0.10; 0.17) |  | -0.01 (-0.15; 0.12) |  | -0.02 (-0.13; 0.09) |
| HbA1c, (%) |  | 0.02 (-0.19; 0.24) |  | -0.03 (-0.09; 0.03) |  | -0.05 (-0.18; 0.06) |  | -0.07 (-0.18; 0.03) |  | 0.01 (-0.09; 0.11) |
| LDL cholesterol, mmol/L |  | **-0.34 (-0.84; -0.30)**** |  | **-0.26 (-0.31; -0.18)**** |  | **-0.25 (-0.43; -0.17)**** |  | **-0.25 (-0.39; -0.15)**** |  | **-0.31 (-0.32; -0.13)**** |
| SES score |  | 0.05 (-0.22; 0.42) |  | **-0.09 (-0.16; -0.02)*** |  | -0.07 (-0.23; 0.06) |  | -0.03 (-0.17; 0.11) |  | -0.10 (-0.20; 0.03) |
| Proline, AU | **0.36**** | **0.17 (0.03; 0.40)*** | **0.29**** | **0.11 (0.05; 0.16)**** | **0.34**** | 0.08 (-0.02; 0.18) | **0.32**** | **0.15 (0.06; 0.28)*** | **0.19**** | 0.05 (-0.05; 0.13) |
| Age, years |  | **-0.22 (-0.45; -0.06)*** |  | **-0.20 (-0.26; -0.13)**** |  | **-0.22 (-0.33; -0.11)**** |  | **-0.20 (-0.34; -0.10)**** |  | **-0.15 (-0.23; -0.02)*** |
| Sex, female/male |  | 0.17 (-0.01; 0.41) |  | **0.09 (0.02; 0.16)*** |  | 0.04 (-0.09; 0.16) |  | 0.07 (-0.05; 0.20) |  | 0.12 (-0.02; 0.22) |
| Ethnicity, Black/White |  | -0.16 (-0.42; 0.02) |  | **-0.17 (-0.25; -0.10)**** |  | **-0.27 (-0.40; -0.14)**** |  | **-0.22 (-0.37; -0.09)**** |  | -0.04 (-0.17; 0.10) |
| Protein intake, g |  | -0.05 (-0.23; 0.11) |  | -0.01 (-0.07; 0.05) |  | 0.05 (-0.06; 0.16) |  | -0.03 (-0.14; 0.08) |  | -0.04 (-0.13; 0.06) |
| Waist-to-height ratio |  | 0.13 (-0.03; 0.42) |  | **0.17 (0.10; 0.22)**** |  | 0.10 (-0.01; 0.30) |  | 0.05 (-0.07; 0.21) |  | **0.21 (0.05; 0.23)*** |
| Physical act, kCal/kg/day |  | -0.06 (-0.33; 0.14) |  | 0.01 (-0.04; 0.07) |  | 0.05 (-0.07; 0.18) |  | 0.01 (-0.09; 0.11) |  | 0.01 (-0.08; 0.09) |
| Cotinine, ng/ml |  |  |  | **0.07 (0.01; 0.12)*** |  | 0.05 (-0.08; 0.22) |  | 0.06 (-0.05; 0.16) |  | 0.03 (-0.07; 0.11) |
| GGT, U/L |  | **-0.29 (-0.61; -0.15)**** |  | **-0.22 (-0.29; -0.16)**** |  | **-0.31 (-0.48; -0.23)**** |  | **-0.19 (-0.36; -0.10)**** |  | **-0.16 (-0.24; -0.03)*** |
| 24h Systolic BP, mmHg |  | -0.06 (-0.34; 0.18) |  | 0.01 (-0.06; 0.08) |  | 0.04 (-0.09; 0.18) |  | -0.01 (-0.14; 0.12) |  | -0.03 (-0.13; 0.09) |
| HbA1c, (%) |  | 0.01 (-0.19; 0.22) |  | -0.04 (-0.10; 0.02) |  | -0.06 (-0.19; 0.05) |  | -0.08 (-0.19; 0.03) |  | 0.01 (-0.10; 0.10) |
| LDL cholesterol, mmol/L |  | **-0.32 (-0.8; -0.27)**** |  | **-0.25 (-0.30; -0.18)**** |  | **-0.24 (-0.42; -0.16)**** |  | **-0.25 (-0.39; -0.15)**** |  | **-0.31 (-0.32; -0.13)**** |
| SES score |  | 0.06 (-0.20; 0.43) |  | **-0.08 (-0.15; -0.01)*** |  | -0.06 (-0.22; 0.06) |  | -0.03 (-0.17; 0.11) |  | -0.10 (-0.20; 0.03) |
| Cystine, AU | **0.34**** | 0.03 (-0.13; 0.20) | **0.29**** | **0.12 (0.06; 0.17)**** | **0.35**** | **0.13 (0.04; 0.26)*** | **0.32**** | **0.18 (0.09; 0.31)**** | **0.19**** | 0.03 (-0.06; 0.11) |
| Age, years |  | **-0.22 (-0.46; -0.06)*** |  | **-0.21 (-0.27; -0.14)**** |  | **-0.22 (-0.34; -0.12)**** |  | **-0.21 (-0.34; -0.10)**** |  | **-0.15 (-0.23; -0.02)*** |
| Sex, female/male |  | 0.14 (-0.05; 0.38) |  | **0.07 (<0.01; 0.14)*** |  | 0.01 (-0.11; 0.14) |  | 0.06 (-0.07; 0.19) |  | 0.12 (-0.02; 0.22) |
| Ethnicity, Black/White |  | -0.14 (-0.40; 0.05) |  | **-0.16 (-0.23; -0.08)**** |  | **-0.26 (-0.40; -0.14)**** |  | **-0.20 (-0.35; -0.07)*** |  | -0.03 (-0.16; 0.11) |
| Protein intake, g |  | -0.07 (-0.25; 0.10) |  | -0.01 (-0.07; 0.05) |  | 0.05 (-0.05; 0.17) |  | -0.03 (-0.14; 0.07) |  | -0.04 (-0.13; 0.06) |
| Waist-to-height ratio |  | 0.12 (-0.05; 0.40) |  | **0.16 (0.09; 0.22)**** |  | 0.09 (-0.01; 0.29) |  | 0.03 (-0.10; 0.19) |  | **0.20 (0.04; 0.23)*** |
| Physical act, kCal/kg/day |  | -0.04 (-0.31; 0.17) |  | 0.01 (-0.04; 0.07) |  | 0.04 (-0.07; 0.17) |  | 0.01 (-0.09; 0.11) |  | 0.01 (-0.08; 0.09) |
| Cotinine, ng/ml |  |  |  | **0.08 (0.02; 0.13)*** |  | 0.06 (-0.06; 0.24) |  | 0.07 (-0.04; 0.17) |  | 0.03 (-0.07; 0.11) |
| GGT, U/L |  | **-0.31 (-0.65; -0.18)**** |  | **-0.23 (-0.30; -0.16)**** |  | **-0.30 (-0.47; -0.21)**** |  | **-0.19 (-0.36; -0.10)**** |  | **-0.17 (-0.24; -0.04)*** |
| 24h Systolic BP, mmHg |  | -0.04 (-0.33; 0.20) |  | 0.01 (-0.06; 0.08) |  | 0.03 (-0.10; 0.17) |  | -0.01 (-0.14; 0.12) |  | -0.02 (-0.13; 0.09) |
| HbA1c, (%) |  | 0.01 (-0.20; 0.23) |  | -0.03 (-0.09; 0.03) |  | -0.05 (-0.18; 0.06) |  | -0.07 (-0.19; 0.03) |  | 0.01 (-0.09; 0.11) |
| LDL cholesterol, mmol/L |  | **-0.35 (-0.85; -0.30)**** |  | **-0.26 (-0.30; -0.18)**** |  | **-0.24 (-0.41; -0.16)**** |  | **-0.26 (-0.41; -0.17)**** |  | **-0.31 (-0.32; -0.13)**** |
| SES score |  | 0.06 (-0.21; 0.44) |  | **-0.08 (-0.15; -0.01)*** |  | -0.06 (-0.22; 0.06) |  | -0.01 (-0.16; 0.13) |  | -0.10 (-0.20; 0.04) |

Test used: Multiple linear regressions. Data are presented as adjusted R^2^ with β coefficient and 95% confidence intervals. Estimated glomerular filtration rate (creatinine-based), adjusted for age, sex, ethnicity, protein intake, waist-to-hight ratio, physical activity, cotinine, GGT, 24h systolic BP, HbA1c, LDL, SES score. Bold values denote P≤0.05; *P≤0.05; **P≤0.001. Cardiovascular disease risk group criteria: Obese - ≥0.55 waist-to-height ratio; Physically inactive - <600 METs for moderate and/or vigorous intensity physical activity; Smoking - ≥11 ng/mL cotinine & self-reported smoking; Excessive alcohol intake - ≥49 U/L GGT & self-reported drinking; Masked hypertensive – normal clinic BP & 24h/day/night BP classified as hypertensive; Hyperglycemic - ≥5.7% HbA1c; Dyslipidemic - >3.4 mmol/L LDL; Low socio-economic – low SES.

*Abbreviations*: AU, arbitrary units; physical act, physical activity; GGT, gamma-glutamyl transferase; BP, blood pressure; HbA1c, glycated haemoglobin; LDL, low density lipoprotein; SES, socio-economic status; eGFR, estimated glomerular filtration rate; CVD, cardiovascular disease.

**Supplementary Table 3F. Supplementary analysis with estimated glomerular filtration rate (creatinine-based) as the dependent variable, with the metabolomics data in control, cardiovascular disease risk group and cardiovascular disease risk clusters**

|  | **eGFR (creatinine), ml/min/1.73m^2^** | | | | | | | | | |
| --- | --- | --- | --- | --- | --- | --- | --- | --- | --- | --- |
|  | **Control group**  **(N=166)** | | **CVD risk group**  **(N=1036)** | | **1 CVD risk factor**  **(N=344)** | | **2 CVD risk factors (N=360)** | | **3+ CVD risk factors (N=332)** | |
| ***Metabolomic data*** | **Adj R^2^** | **β (95%Cl)** | **Adj R^2^** | **β (95%Cl)** | **Adj R^2^** | **β (95%Cl)** | **Adj R^2^** | **β (95%Cl)** | **Adj R^2^** | **β (95%Cl)** |
| Valine, AU | **0.36**** | **0.18 (0.04; 0.40)*** | **0.30**** | **0.16 (0.10; 0.21)**** | **0.34**** | **0.12 (0.03; 0.23)*** | **0.34**** | **0.21 (0.12; 0.32)**** | **0.19**** | 0.10 (-0.01; 0.17) |
| Age, years |  | **-0.23 (-0.46; -0.08)*** |  | **-0.20 (-0.26; -0.14)**** |  | **-0.21 (-0.32; -0.10)**** |  | **-0.21 (-0.34; -0.10)**** |  | **-0.16 (-0.24; -0.03)*** |
| Sex, female/male |  | 0.16 (-0.02; 0.40) |  | **0.08 (0.01; 0.15)*** |  | 0.02 (-0.10; 0.15) |  | 0.08 (-0.04; 0.20) |  | 0.12 (-0.02; 0.22) |
| Ethnicity, Black/White |  | -0.15 (-0.41; 0.02) |  | **-0.18 (-0.25; -0.10)**** |  | **-0.26 (-0.40; -0.14)**** |  | **-0.23 (-0.38; -0.11)**** |  | -0.04 (-0.17; 0.10) |
| Protein intake, g |  | -0.10 (-0.28; 0.06) |  | -0.01 (-0.07; 0.05) |  | 0.04 (-0.06; 0.16) |  | -0.04 (-0.15; 0.06) |  | -0.03 (-0.12; 0.07) |
| Waist-to-height ratio |  | 0.11 (-0.05; 0.39) |  | **0.15 (0.08; 0.21)**** |  | 0.09 (-0.02; 0.29) |  | 0.05 (-0.07; 0.21) |  | **0.21 (0.05; 0.23)*** |
| Physical act, kCal/kg/day |  | -0.06 (-0.32; 0.14) |  | 0.01 (-0.05; 0.06) |  | 0.04 (-0.07; 0.17) |  | <0.01 (-0.10; 0.09) |  | 0.01 (-0.08; 0.09) |
| Cotinine, ng/ml |  |  |  | **0.08 (0.02; 0.13)*** |  | 0.05 (-0.07; 0.22) |  | 0.08 (-0.02; 0.19) |  | 0.04 (-0.06; 0.12) |
| GGT, U/L |  | **-0.29 (-0.62; -0.16)**** |  | **-0.23 (-0.29; -0.16)**** |  | **-0.31 (-0.48; -0.22)**** |  | **-0.20 (-0.38; -0.12)**** |  | **-0.16 (-0.24; -0.04)*** |
| 24h Systolic BP, mmHg |  | -0.06 (-0.35; 0.17) |  | 0.01 (-0.06; 0.08) |  | 0.04 (-0.09; 0.18) |  | -0.01 (-0.14; 0.12) |  | -0.03 (-0.13; 0.09) |
| HbA1c, (%) |  | 0.02 (-0.18; 0.23) |  | -0.04 (-0.09; 0.03) |  | -0.06 (-0.19; 0.05) |  | -0.07 (-0.18; 0.03) |  | 0.01 (-0.09; 0.11) |
| LDL cholesterol, mmol/L |  | **-0.32 (-0.80; -0.27)**** |  | **-0.25 (-0.30; -0.17)**** |  | **-0.24 (-0.41; -0.16)**** |  | **-0.24 (-0.38; -0.15)**** |  | **-0.30 (-0.32; -0.13)**** |
| SES score |  | 0.06 (-0.19; 0.43) |  | **-0.09 (-0.16; -0.02)*** |  | -0.08 (-0.24; 0.05) |  | -0.03 (-0.16; 0.11) |  | -0.11 (-0.21; 0.02) |
| Methionine, AU | **0.34**** | 0.08 (-0.09; 0.31) | **0.29**** | **0.12 (0.06; 0.17)**** | **0.34**** | 0.09 (-0.01; 0.20) | **0.32**** | **0.18 (0.08; 0.29)**** | **0.19**** | 0.05 (-0.05; 0.13) |
| Age, years |  | **-0.23 (-0.46; -0.07)*** |  | **-0.21 (-0.26; -0.14)**** |  | **-0.21 (-0.33; -0.10)**** |  | **-0.21 (-0.34; -0.10)**** |  | **-0.16 (-0.23; -0.03)*** |
| Sex, female/male |  | 0.15 (-0.04; 0.39) |  | **0.08 (0.01; 0.15)*** |  | 0.03 (-0.09; 0.15) |  | 0.07 (-0.06; 0.20) |  | 0.11 (-0.02; 0.22) |
| Ethnicity, Black/White |  | -0.13 (-0.4; 0.05) |  | **-0.15 (-0.22; -0.07)**** |  | **-0.25 (-0.38; -0.12)**** |  | **-0.18 (-0.32; -0.05)*** |  | -0.03 (-0.16; 0.11) |
| Protein intake, g |  | -0.07 (-0.25; 0.10) |  | -0.01 (-0.07; 0.05) |  | 0.05 (-0.06; 0.16) |  | -0.03 (-0.14; 0.08) |  | -0.04 (-0.13; 0.06) |
| Waist-to-height ratio |  | 0.12 (-0.04; 0.41) |  | **0.17 (0.10; 0.23)**** |  | 0.10 (<0.01; 0.30) |  | 0.07 (-0.06; 0.23) |  | **0.21 (0.05; 0.23)*** |
| Physical act, kCal/kg/day |  | -0.04 (-0.31; 0.17) |  | 0.01 (-0.04; 0.07) |  | 0.04 (-0.07; 0.18) |  | <0.01 (-0.10; 0.10) |  | 0.01 (-0.07; 0.10) |
| Cotinine, ng/ml |  |  |  | **0.08 (0.02; 0.13)*** |  | 0.05 (-0.07; 0.23) |  | 0.07 (-0.03; 0.18) |  | 0.04 (-0.06; 0.12) |
| GGT, U/L |  | **-0.31 (-0.64; -0.17)**** |  | **-0.23 (-0.29; -0.16)**** |  | **-0.31 (-0.48; -0.22)**** |  | **-0.20 (-0.37; -0.11)**** |  | **-0.16 (-0.24; -0.04)*** |
| 24h Systolic BP, mmHg |  | -0.05 (-0.34; 0.19) |  | 0.01 (-0.06; 0.08) |  | 0.03 (-0.10; 0.17) |  | -0.01 (-0.15; 0.12) |  | -0.02 (-0.13; 0.09) |
| HbA1c, (%) |  | 0.01 (-0.20; 0.23) |  | -0.04 (-0.10; 0.02) |  | -0.06 (-0.19; 0.05) |  | -0.08 (-0.19; 0.02) |  | 0.01 (-0.09; 0.11) |
| LDL cholesterol, mmol/L |  | **-0.33 (-0.82; -0.27)**** |  | **-0.25 (-0.30; -0.18)**** |  | **-0.24 (-0.42; -0.16)**** |  | **-0.25 (-0.39; -0.15)**** |  | **-0.31 (-0.32; -0.13)**** |
| SES score |  | 0.06 (-0.20; 0.44) |  | **-0.09 (-0.16; -0.02)*** |  | -0.07 (-0.23; 0.06) |  | -0.03 (-0.17; 0.11) |  | -0.10 (-0.20; 0.03) |
| Tyrosine, AU | **0.36**** | 0.15 (<0.01; 0.34) | **0.30**** | **0.14 (0.08; 0.20)**** | **0.34**** | **0.12 (0.02; 0.22)*** | **0.33**** | **0.19 (0.10; 0.31)**** | **0.19**** | 0.09 (-0.02; 0.17) |
| Age, years |  | **-0.22 (-0.45; -0.06)*** |  | **-0.20 (-0.26; -0.14)**** |  | **-0.22 (-0.33; -0.11)**** |  | **-0.20 (-0.33; -0.09)**** |  | **-0.16 (-0.23; -0.03)*** |
| Sex, female/male |  | 0.16 (-0.03; 0.40) |  | **0.08 (0.01; 0.15)*** |  | 0.02 (-0.10; 0.14) |  | 0.07 (-0.05; 0.20) |  | 0.12 (-0.02; 0.22) |
| Ethnicity, Black/White |  | -0.16 (-0.43; 0.01) |  | **-0.17 (-0.24; -0.09)**** |  | **-0.26 (-0.40; -0.14)**** |  | **-0.21 (-0.36; -0.09)**** |  | -0.03 (-0.16; 0.11) |
| Protein intake, g |  | -0.08 (-0.26; 0.08) |  | -0.01 (-0.07; 0.05) |  | 0.05 (-0.06; 0.16) |  | -0.03 (-0.14; 0.08) |  | -0.04 (-0.13; 0.07) |
| Waist-to-height ratio |  | 0.12 (-0.04; 0.40) |  | **0.15 (0.08; 0.21)**** |  | 0.09 (-0.02; 0.29) |  | 0.04 (-0.09; 0.20) |  | **0.20 (0.04; 0.23)*** |
| Physical act, kCal/kg/day |  | -0.05 (-0.32; 0.15) |  | 0.01 (-0.04; 0.07) |  | 0.04 (-0.07; 0.17) |  | <0.01 (-0.10; 0.10) |  | 0.01 (-0.07; 0.10) |
| Cotinine, ng/ml |  |  |  | **0.08 (0.02; 0.13)*** |  | 0.05 (-0.07; 0.23) |  | 0.08 (-0.03; 0.18) |  | 0.04 (-0.06; 0.12) |
| GGT, U/L |  | **-0.32 (-0.65; -0.19)**** |  | **-0.23 (-0.30; -0.16)**** |  | **-0.31 (-0.48; -0.22)**** |  | **-0.20 (-0.38; -0.12)**** |  | **-0.17 (-0.24; -0.04)*** |
| 24h Systolic BP, mmHg |  | -0.07 (-0.35; 0.17) |  | <0.01 (-0.07; 0.07) |  | 0.04 (-0.09; 0.18) |  | -0.02 (-0.15; 0.11) |  | -0.03 (-0.13; 0.09) |
| HbA1c, (%) |  | 0.02 (-0.18; 0.24) |  | -0.04 (-0.09; 0.03) |  | -0.06 (-0.19; 0.05) |  | -0.07 (-0.18; 0.03) |  | 0.01 (-0.09; 0.11) |
| LDL cholesterol, mmol/L |  | **-0.32 (-0.80; -0.26)**** |  | **-0.25 (-0.29; -0.17)**** |  | **-0.23 (-0.41; -0.15)**** |  | **-0.24 (-0.38; -0.14)**** |  | **-0.30 (-0.31; -0.12)**** |
| SES score |  | 0.06 (-0.20; 0.43) |  | **-0.09 (-0.15; -0.01)*** |  | -0.07 (-0.23; 0.06) |  | -0.03 (-0.17; 0.11) |  | -0.11 (-0.21; 0.03) |
| Pyroglutamic acid, AU | **0.35**** | 0.10 (-0.06; 0.31) | **0.29**** | **0.11 (0.05; 0.16)**** | **0.34**** | **0.10 (<0.01; 0.21)*** | **0.32**** | **0.15 (0.06; 0.27)*** | **0.19**** | 0.05 (-0.05; 0.13) |
| Age, years |  | **-0.22 (-0.46; -0.06)*** |  | **-0.21 (-0.27; -0.14)**** |  | **-0.22 (-0.33; -0.11)**** |  | **-0.22 (-0.35; -0.11)**** |  | **-0.16 (-0.23; -0.03)*** |
| Sex, female/male |  | 0.15 (-0.04; 0.39) |  | **0.08 (0.01; 0.15)*** |  | 0.02 (-0.10; 0.15) |  | 0.06 (-0.06; 0.19) |  | 0.12 (-0.02; 0.22) |
| Ethnicity, Black/White |  | -0.13 (-0.40; 0.05) |  | **-0.16 (-0.23; -0.08)**** |  | **-0.25 (-0.39; -0.13)**** |  | **-0.20 (-0.35; -0.08)*** |  | -0.03 (-0.16; 0.11) |
| Protein intake, g |  | -0.07 (-0.25; 0.09) |  | -0.01 (-0.07; 0.05) |  | 0.05 (-0.06; 0.16) |  | -0.03 (-0.14; 0.08) |  | -0.04 (-0.13; 0.06) |
| Waist-to-height ratio |  | 0.12 (-0.05; 0.40) |  | **0.17 (0.10; 0.23)**** |  | 0.10 (<0.01; 0.30) |  | 0.06 (-0.07; 0.22) |  | **0.21 (0.05; 0.24)*** |
| Physical act, kCal/kg/day |  | -0.05 (-0.31; 0.16) |  | 0.01 (-0.05; 0.06) |  | 0.04 (-0.07; 0.17) |  | <0.01 (-0.10; 0.10) |  | 0.01 (-0.08; 0.09) |
| Cotinine, ng/ml |  |  |  | **0.08 (0.02; 0.13)*** |  | 0.05 (-0.07; 0.22) |  | 0.07 (-0.04; 0.17) |  | 0.03 (-0.06; 0.11) |
| GGT, U/L |  | **-0.30 (-0.64; -0.17)**** |  | **-0.23 (-0.29; -0.16)**** |  | **-0.31 (-0.48; -0.22)**** |  | **-0.20 (-0.37; -0.11)**** |  | **-0.16 (-0.24; -0.04)*** |
| 24h Systolic BP, mmHg |  | -0.05 (-0.34; 0.19) |  | 0.01 (-0.06; 0.08) |  | 0.04 (-0.09; 0.18) |  | -0.01 (-0.14; 0.12) |  | -0.03 (-0.13; 0.09) |
| HbA1c, (%) |  | 0.01 (-0.19; 0.23) |  | -0.03 (-0.09; 0.03) |  | -0.06 (-0.18; 0.06) |  | -0.08 (-0.19; 0.03) |  | 0.01 (-0.09; 0.11) |
| LDL cholesterol, mmol/L |  | **-0.33 (-0.82; -0.28)**** |  | **-0.25 (-0.30; -0.17)**** |  | **-0.24 (-0.41; -0.16)**** |  | **-0.24 (-0.38; -0.14)**** |  | **-0.31 (-0.32; -0.13)**** |
| SES score |  | 0.06 (-0.21; 0.43) |  | **-0.08 (-0.15; -0.01)*** |  | -0.07 (-0.22; 0.06) |  | -0.03 (-0.17; 0.11) |  | -0.10 (-0.20; 0.03) |

Test used: Multiple linear regressions. Data are presented as adjusted R^2^ with β coefficient and 95% confidence intervals. Estimated glomerular filtration rate (creatinine-based), adjusted for age, sex, ethnicity, protein intake, waist-to-hight ratio, physical activity, cotinine, GGT, 24h systolic BP, HbA1c, LDL, SES score. Bold values denote P≤0.05; *P≤0.05; **P≤0.001. Cardiovascular disease risk group criteria: Obese - ≥0.55 waist-to-height ratio; Physically inactive - <600 METs for moderate and/or vigorous intensity physical activity; Smoking - ≥11 ng/mL cotinine & self-reported smoking; Excessive alcohol intake - ≥49 U/L GGT & self-reported drinking; Masked hypertensive – normal clinic BP & 24h/day/night BP classified as hypertensive; Hyperglycemic - ≥5.7% HbA1c; Dyslipidemic - >3.4 mmol/L LDL; Low socio-economic – low SES.

*Abbreviations*: AU, arbitrary units; physical act, physical activity; GGT, gamma-glutamyl transferase; BP, blood pressure; HbA1c, glycated haemoglobin; LDL, low density lipoprotein; SES, socio-economic status; eGFR, estimated glomerular filtration rate; CVD, cardiovascular disease.

**Supplementary Table 3G. Supplementary analysis with estimated glomerular filtration rate (creatinine-based) as the dependent variable, with the metabolomics data in control, cardiovascular disease risk group and cardiovascular disease risk clusters**

|  | **eGFR (creatinine), ml/min/1.73m^2^** | | | | | | | | | |
| --- | --- | --- | --- | --- | --- | --- | --- | --- | --- | --- |
|  | **Control group**  **(N=166)** | | **CVD risk group**  **(N=1036)** | | **1 CVD risk factor**  **(N=344)** | | **2 CVD risk factors (N=360)** | | **3+ CVD risk factors (N=332)** | |
| ***Metabolomic data*** | **Adj R^2^** | **β (95%Cl)** | **Adj R^2^** | **β (95%Cl)** | **Adj R^2^** | **β (95%Cl)** | **Adj R^2^** | **β (95%Cl)** | **Adj R^2^** | **β (95%Cl)** |
| Leucine/Isoleucine, AU | **0.36**** | **0.17 (0.02; 0.39)*** | **0.29**** | **0.14 (0.08; 0.19)**** | **0.34**** | 0.10 (<0.01; 0.19) | **0.33**** | **0.20 (0.11; 0.32)**** | **0.19**** | 0.10 (-0.01; 0.17) |
| Age, years |  | **-0.22 (-0.45; -0.07)*** |  | **-0.20 (-0.26; -0.13)**** |  | **-0.21 (-0.32; -0.10)**** |  | **-0.20 (-0.34; -0.10)**** |  | **-0.16 (-0.24; -0.03)*** |
| Sex, female/male |  | 0.15 (-0.04; 0.38) |  | **0.08 (0.01; 0.14)*** |  | 0.02 (-0.10; 0.15) |  | 0.06 (-0.06; 0.19) |  | 0.11 (-0.02; 0.21) |
| Ethnicity, Black/White |  | -0.15 (-0.41; 0.03) |  | **-0.17 (-0.24; -0.09)**** |  | **-0.26 (-0.40; -0.14)**** |  | **-0.22 (-0.37; -0.10)**** |  | -0.04 (-0.16; 0.10) |
| Protein intake, g |  | -0.09 (-0.27; 0.07) |  | -0.01 (-0.07; 0.05) |  | 0.04 (-0.06; 0.16) |  | -0.04 (-0.15; 0.07) |  | -0.03 (-0.12; 0.07) |
| Waist-to-height ratio |  | 0.12 (-0.05; 0.39) |  | **0.16 (0.09; 0.21)**** |  | 0.09 (-0.02; 0.29) |  | 0.05 (-0.08; 0.20) |  | **0.21 (0.05; 0.23)*** |
| Physical act, kCal/kg/day |  | -0.06 (-0.34; 0.14) |  | 0.01 (-0.05; 0.06) |  | 0.04 (-0.07; 0.17) |  | -0.01 (-0.10; 0.09) |  | 0.01 (-0.08; 0.09) |
| Cotinine, ng/ml |  |  |  | **0.08 (0.02; 0.13)*** |  | 0.05 (-0.08; 0.22) |  | 0.08 (-0.03; 0.18) |  | 0.04 (-0.06; 0.12) |
| GGT, U/L |  | **-0.30 (-0.63; -0.17)**** |  | **-0.23 (-0.29; -0.16)**** |  | **-0.32 (-0.48; -0.23)**** |  | **-0.20 (-0.37; -0.12)**** |  | **-0.16 (-0.24; -0.03)*** |
| 24h Systolic BP, mmHg |  | -0.06 (-0.35; 0.17) |  | 0.01 (-0.06; 0.07) |  | 0.04 (-0.09; 0.18) |  | -0.02 (-0.15; 0.11) |  | -0.03 (-0.13; 0.09) |
| HbA1c, (%) |  | 0.01 (-0.19; 0.23) |  | -0.04 (-0.10; 0.02) |  | -0.06 (-0.18; 0.06) |  | -0.08 (-0.19; 0.02) |  | 0.01 (-0.09; 0.11) |
| LDL cholesterol, mmol/L |  | **-0.32 (-0.80; -0.27)**** |  | **-0.25 (-0.30; -0.17)**** |  | **-0.24 (-0.42; -0.16)**** |  | **-0.24 (-0.38; -0.14)**** |  | **-0.30 (-0.32; -0.13)**** |
| SES score |  | 0.06 (-0.20; 0.43) |  | **-0.09 (-0.16; -0.02)*** |  | -0.08 (-0.24; 0.05) |  | -0.03 (-0.17; 0.11) |  | -0.11 (-0.21; 0.03) |
| Phenylalanine, AU | **0.36**** | **0.15 (<0.01; 0.35)*** | **0.30**** | **0.16 (0.10; 0.21)**** | **0.35**** | **0.14 (0.05; 0.25)*** | **0.34**** | **0.22 (0.13; 0.33)**** | **0.19**** | 0.09 (-0.02; 0.17) |
| Age, years |  | **-0.22 (-0.45; -0.06)*** |  | **-0.20 (-0.26; -0.13)**** |  | **-0.21 (-0.32; -0.10)**** |  | **-0.20 (-0.33; -0.10)**** |  | **-0.16 (-0.23; -0.03)*** |
| Sex, female/male |  | 0.15 (-0.04; 0.39) |  | **0.08 (0.01; 0.15)*** |  | 0.02 (-0.10; 0.15) |  | 0.07 (-0.05; 0.20) |  | 0.12 (-0.02; 0.22) |
| Ethnicity, Black/White |  | -0.15 (-0.42; 0.02) |  | **-0.16 (-0.23; -0.09)**** |  | **-0.25 (-0.39; -0.13)**** |  | **-0.20 (-0.35; -0.08)**** |  | -0.03 (-0.16; 0.10) |
| Protein intake, g |  | -0.08 (-0.27; 0.08) |  | -0.01 (-0.07; 0.05) |  | 0.05 (-0.06; 0.16) |  | -0.02 (-0.13; 0.08) |  | -0.04 (-0.13; 0.07) |
| Waist-to-height ratio |  | 0.12 (-0.05; 0.39) |  | **0.15 (0.08; 0.21)**** |  | 0.09 (-0.02; 0.29) |  | 0.04 (-0.09; 0.20) |  | **0.20 (0.04; 0.23)*** |
| Physical act, kCal/kg/day |  | -0.05 (-0.31; 0.16) |  | 0.01 (-0.04; 0.06) |  | 0.04 (-0.07; 0.17) |  | <0.01 (-0.10; 0.10) |  | 0.01 (-0.07; 0.09) |
| Cotinine, ng/ml |  |  |  | **0.08 (0.02; 0.13)*** |  | 0.05 (-0.07; 0.22) |  | 0.08 (-0.02; 0.19) |  | 0.04 (-0.06; 0.12) |
| GGT, U/L |  | **-0.31 (-0.64; -0.18)**** |  | **-0.23 (-0.29; -0.16)**** |  | **-0.31 (-0.48; -0.22)**** |  | **-0.20 (-0.37; -0.12)**** |  | **-0.16 (-0.24; -0.04)*** |
| 24h Systolic BP, mmHg |  | -0.07 (-0.36; 0.17) |  | <0.01 (-0.07; 0.07) |  | 0.03 (-0.10; 0.17) |  | -0.02 (-0.15; 0.10) |  | -0.02 (-0.13; 0.09) |
| HbA1c, (%) |  | 0.01 (-0.19; 0.23) |  | -0.04 (-0.10; 0.02) |  | -0.06 (-0.19; 0.05) |  | -0.08 (-0.19; 0.02) |  | 0.01 (-0.09; 0.11) |
| LDL cholesterol, mmol/L |  | **-0.32 (-0.80; -0.26)**** |  | **-0.24 (-0.29; -0.17)**** |  | **-0.23 (-0.40; -0.14)**** |  | **-0.25 (-0.38; -0.15)**** |  | **-0.30 (-0.31; -0.12)**** |
| SES score |  | 0.05 (-0.21; 0.42) |  | **-0.09 (-0.16; -0.02)*** |  | -0.08 (-0.24; 0.05) |  | -0.03 (-0.16; 0.11) |  | -0.11 (-0.21; 0.03) |
| Aspartic acid, AU | **0.35**** | 0.11 (-0.04; 0.35) | **0.28**** | **0.09 (0.03; 0.15)*** | **0.34**** | **0.10 (<0.01; 0.21)*** | **0.30**** | **0.10 (<0.01; 0.20)*** | **0.19**** | 0.05 (-0.05; 0.14) |
| Age, years |  | **-0.22 (-0.46; -0.06)*** |  | **-0.21 (-0.27; -0.14)**** |  | **-0.22 (-0.34; -0.11)**** |  | **-0.21 (-0.35; -0.11)**** |  | **-0.16 (-0.23; -0.03)*** |
| Sex, female/male |  | 0.15 (-0.04; 0.38) |  | **0.09 (0.02; 0.15)*** |  | 0.04 (-0.08; 0.16) |  | 0.07 (-0.06; 0.20) |  | 0.12 (-0.02; 0.22) |
| Ethnicity, Black/White |  | -0.14 (-0.41; 0.03) |  | **-0.16 (-0.24; -0.09)**** |  | **-0.26 (-0.40; -0.14)**** |  | **-0.20 (-0.35; -0.07)*** |  | -0.03 (-0.16; 0.11) |
| Protein intake, g |  | -0.07 (-0.25; 0.10) |  | -0.02 (-0.07; 0.04) |  | 0.04 (-0.07; 0.15) |  | -0.04 (-0.15; 0.07) |  | -0.04 (-0.13; 0.06) |
| Waist-to-height ratio |  | 0.12 (-0.04; 0.41) |  | **0.16 (0.09; 0.22)**** |  | 0.10 (-0.01; 0.30) |  | 0.05 (-0.08; 0.21) |  | **0.21 (0.05; 0.23)*** |
| Physical act, kCal/kg/day |  | -0.05 (-0.32; 0.15) |  | 0.01 (-0.04; 0.07) |  | 0.04 (-0.07; 0.17) |  | <0.01 (-0.10; 0.10) |  | 0.01 (-0.08; 0.09) |
| Cotinine, ng/ml |  |  |  | **0.08 (0.02; 0.13)*** |  | 0.05 (-0.08; 0.22) |  | 0.07 (-0.04; 0.18) |  | 0.03 (-0.06; 0.11) |
| GGT, U/L |  | **-0.30 (-0.63; -0.17)**** |  | **-0.23 (-0.30; -0.16)**** |  | **-0.31 (-0.48; -0.22)**** |  | **-0.20 (-0.37; -0.11)**** |  | **-0.16 (-0.24; -0.03)*** |
| 24h Systolic BP, mmHg |  | -0.05 (-0.33; 0.19) |  | 0.01 (-0.06; 0.08) |  | 0.04 (-0.09; 0.18) |  | <0.01 (-0.14; 0.13) |  | -0.03 (-0.13; 0.09) |
| HbA1c, (%) |  | 0.01 (-0.20; 0.22) |  | -0.04 (-0.10; 0.03) |  | -0.06 (-0.19; 0.05) |  | -0.08 (-0.19; 0.03) |  | 0.01 (-0.09; 0.11) |
| LDL cholesterol, mmol/L |  | **-0.34 (-0.82; -0.29)**** |  | **-0.25 (-0.30; -0.18)**** |  | **-0.24 (-0.41; -0.16)**** |  | **-0.25 (-0.39; -0.15)**** |  | **-0.31 (-0.32; -0.13)**** |
| SES score |  | 0.06 (-0.21; 0.43) |  | **-0.09 (-0.15; -0.01)*** |  | -0.06 (-0.22; 0.06) |  | -0.04 (-0.18; 0.10) |  | -0.10 (-0.20; 0.03) |
| Tryptophan, AU | **0.36**** | 0.15 (<0.01; 0.36) | **0.30**** | **0.14 (0.08; 0.19)**** | **0.35**** | **0.13 (0.04; 0.24)*** | **0.33**** | **0.19 (0.10; 0.29)**** | **0.19**** | 0.06 (-0.04; 0.15) |
| Age, years |  | **-0.22 (-0.45; -0.06)*** |  | **-0.20 (-0.26; -0.13)**** |  | **-0.21 (-0.33; -0.11)**** |  | **-0.20 (-0.33; -0.09)**** |  | **-0.15 (-0.23; -0.03)*** |
| Sex, female/male |  | 0.15 (-0.04; 0.39) |  | **0.08 (0.01; 0.15)*** |  | 0.02 (-0.10; 0.15) |  | 0.07 (-0.05; 0.20) |  | 0.12 (-0.02; 0.22) |
| Ethnicity, Black/White |  | -0.17 (-0.44; 0.01) |  | **-0.17 (-0.25; -0.10)**** |  | **-0.27 (-0.40; -0.14)**** |  | **-0.22 (-0.37; -0.09)**** |  | -0.04 (-0.17; 0.10) |
| Protein intake, g |  | -0.08 (-0.26; 0.09) |  | -0.01 (-0.07; 0.05) |  | 0.05 (-0.06; 0.16) |  | -0.03 (-0.14; 0.07) |  | -0.04 (-0.13; 0.06) |
| Waist-to-height ratio |  | 0.12 (-0.05; 0.40) |  | **0.16 (0.09; 0.21)**** |  | 0.09 (-0.02; 0.29) |  | 0.04 (-0.09; 0.20) |  | **0.20 (0.04; 0.23)*** |
| Physical act, kCal/kg/day |  | -0.05 (-0.32; 0.15) |  | 0.01 (-0.05; 0.06) |  | 0.04 (-0.07; 0.17) |  | <0.01 (-0.10; 0.09) |  | 0.01 (-0.08; 0.09) |
| Cotinine, ng/ml |  |  |  | **0.08 (0.02; 0.13)*** |  | 0.05 (-0.07; 0.22) |  | 0.07 (-0.04; 0.17) |  | 0.04 (-0.06; 0.12) |
| GGT, U/L |  | **-0.32 (-0.65; -0.19)**** |  | **-0.23 (-0.30; -0.17)**** |  | **-0.31 (-0.47; -0.22)**** |  | **-0.21 (-0.38; -0.13)**** |  | **-0.17 (-0.24; -0.04)*** |
| 24h Systolic BP, mmHg |  | -0.06 (-0.35; 0.17) |  | 0.01 (-0.06; 0.07) |  | 0.04 (-0.09; 0.17) |  | -0.01 (-0.14; 0.12) |  | -0.03 (-0.13; 0.09) |
| HbA1c, (%) |  | 0.02 (-0.18; 0.25) |  | -0.04 (-0.10; 0.03) |  | -0.06 (-0.19; 0.05) |  | -0.07 (-0.18; 0.03) |  | 0.01 (-0.09; 0.11) |
| LDL cholesterol, mmol/L |  | **-0.32 (-0.80; -0.26)**** |  | **-0.25 (-0.30; -0.17)**** |  | **-0.23 (-0.40; -0.15)**** |  | **-0.25 (-0.39; -0.16)**** |  | **-0.30 (-0.32; -0.13)**** |
| SES score |  | 0.05 (-0.22; 0.42) |  | **-0.09 (-0.16; -0.02)*** |  | -0.08 (-0.24; 0.05) |  | -0.04 (-0.18; 0.10) |  | -0.10 (-0.20; 0.03) |

Test used: Multiple linear regressions. Data are presented as adjusted R^2^ with β coefficient and 95% confidence intervals. Estimated glomerular filtration rate (creatinine-based), adjusted for age, sex, ethnicity, protein intake, waist-to-hight ratio, physical activity, cotinine, GGT, 24h systolic BP, HbA1c, LDL, SES score. Bold values denote P≤0.05; *P≤0.05; **P≤0.001. Cardiovascular disease risk group criteria: Obese - ≥0.55 waist-to-height ratio; Physically inactive - <600 METs for moderate and/or vigorous intensity physical activity; Smoking - ≥11 ng/mL cotinine & self-reported smoking; Excessive alcohol intake - ≥49 U/L GGT & self-reported drinking; Masked hypertensive – normal clinic BP & 24h/day/night BP classified as hypertensive; Hyperglycemic - ≥5.7% HbA1c; Dyslipidemic - >3.4 mmol/L LDL; Low socio-economic – low SES.

*Abbreviations*: AU, arbitrary units; physical act, physical activity; GGT, gamma-glutamyl transferase; BP, blood pressure; HbA1c, glycated haemoglobin; LDL, low density lipoprotein; SES, socio-economic status; eGFR, estimated glomerular filtration rate; CVD, cardiovascular disease.

**Supplementary Table 3H. Supplementary analysis with estimated glomerular filtration rate (creatinine-based) as the dependent variable, with the metabolomics data in control, cardiovascular disease risk group and cardiovascular disease risk clusters**

|  | **eGFR (creatinine), ml/min/1.73m^2^** | | | | | | | | | |
| --- | --- | --- | --- | --- | --- | --- | --- | --- | --- | --- |
|  | **Control group**  **(N=166)** | | **CVD risk group**  **(N=1036)** | | **1 CVD risk factor**  **(N=344)** | | **2 CVD risk factors (N=360)** | | **3+ CVD risk factors (N=332)** | |
| ***Metabolomic data*** | **Adj R^2^** | **β (95%Cl)** | **Adj R^2^** | **β (95%Cl)** | **Adj R^2^** | **β (95%Cl)** | **Adj R^2^** | **β (95%Cl)** | **Adj R^2^** | **β (95%Cl)** |
| Glutamic acid, AU | **0.35**** | 0.14 (-0.01; 0.35) | **0.29**** | **0.12 (0.06; 0.17)**** | **0.34**** | **0.10 (<0.01; 0.20)*** | **0.32**** | **0.17 (0.08; 0.28)**** | **0.19**** | 0.05 (-0.05; 0.14) |
| Age, years |  | **-0.23 (-0.46; -0.07)*** |  | **-0.21 (-0.27; -0.14)**** |  | **-0.22 (-0.33; -0.11)**** |  | **-0.22 (-0.35; -0.11)**** |  | **-0.16 (-0.23; -0.03)*** |
| Sex, female/male |  | 0.16 (-0.03; 0.39) |  | **0.09 (0.02; 0.15)*** |  | 0.03 (-0.09; 0.15) |  | 0.08 (-0.04; 0.21) |  | 0.12 (-0.02; 0.22) |
| Ethnicity, Black/White |  | -0.14 (-0.40; 0.04) |  | **-0.16 (-0.24; -0.09)**** |  | **-0.25 (-0.39; -0.13)**** |  | **-0.20 (-0.35; -0.08)**** |  | -0.03 (-0.16; 0.10) |
| Protein intake, g |  | -0.07 (-0.25; 0.10) |  | -0.01 (-0.07; 0.05) |  | 0.05 (-0.06; 0.16) |  | -0.03 (-0.14; 0.08) |  | -0.04 (-0.13; 0.07) |
| Waist-to-height ratio |  | 0.13 (-0.03; 0.41) |  | **0.17 (0.10; 0.23)**** |  | 0.10 (-0.01; 0.30) |  | 0.06 (-0.07; 0.22) |  | **0.21 (0.05; 0.24)*** |
| Physical act, kCal/kg/day |  | -0.06 (-0.33; 0.15) |  | 0.01 (-0.05; 0.06) |  | 0.05 (-0.06; 0.18) |  | <0.01 (-0.10; 0.10) |  | 0.01 (-0.08; 0.09) |
| Cotinine, ng/ml |  |  |  | **0.07 (0.01; 0.12)*** |  | 0.05 (-0.07; 0.22) |  | 0.06 (-0.05; 0.17) |  | 0.03 (-0.07; 0.11) |
| GGT, U/L |  | **-0.30 (-0.63; -0.16)**** |  | **-0.22 (-0.29; -0.16)**** |  | **-0.31 (-0.48; -0.22)**** |  | **-0.20 (-0.37; -0.11)**** |  | **-0.16 (-0.24; -0.03)*** |
| 24h Systolic BP, mmHg |  | -0.06 (-0.35; 0.18) |  | 0.01 (-0.06; 0.08) |  | 0.04 (-0.09; 0.17) |  | -0.01 (-0.14; 0.12) |  | -0.03 (-0.13; 0.09) |
| HbA1c, (%) |  | 0.01 (-0.19; 0.23) |  | -0.03 (-0.09; 0.03) |  | -0.05 (-0.18; 0.06) |  | -0.08 (-0.19; 0.02) |  | 0.01 (-0.09; 0.11) |
| LDL cholesterol, mmol/L |  | **-0.33 (-0.81; -0.28)**** |  | **-0.25 (-0.30; -0.17)**** |  | **-0.24 (-0.41; -0.15)**** |  | **-0.24 (-0.38; -0.14)**** |  | **-0.31 (-0.32; -0.13)**** |
| SES score |  | 0.06 (-0.20; 0.43) |  | **-0.08 (-0.15; -0.01)*** |  | -0.07 (-0.22; 0.06) |  | -0.02 (-0.16; 0.12) |  | -0.10 (-0.20; 0.03) |
| 2-Aminoadipic acid, AU | **0.37**** | **0.18 (0.04; 0.39)*** | **0.29**** | **0.12 (0.06; 0.17)**** | **0.34**** | **0.11 (0.01; 0.21)*** | **0.32**** | **0.17 (0.07; 0.28)**** | **0.19**** | 0.07 (-0.03; 0.15) |
| Age, years |  | **-0.23 (-0.46; -0.08)*** |  | **-0.20 (-0.26; -0.14)**** |  | **-0.22 (-0.33; -0.11)**** |  | **-0.20 (-0.33; -0.09)**** |  | **-0.16 (-0.24; -0.03)*** |
| Sex, female/male |  | 0.14 (-0.05; 0.37) |  | **0.07 (<0.01; 0.14)*** |  | 0.01 (-0.11; 0.14) |  | 0.06 (-0.06; 0.19) |  | 0.11 (-0.02; 0.22) |
| Ethnicity, Black/White |  | -0.17 (-0.44; <0.01) |  | **-0.17 (-0.24; -0.09)**** |  | **-0.26 (-0.40; -0.14)**** |  | **-0.22 (-0.38; -0.10)**** |  | -0.03 (-0.16; 0.10) |
| Protein intake, g |  | -0.09 (-0.27; 0.07) |  | -0.02 (-0.08; 0.04) |  | 0.04 (-0.07; 0.15) |  | -0.05 (-0.16; 0.05) |  | -0.05 (-0.13; 0.06) |
| Waist-to-height ratio |  | 0.11 (-0.05; 0.39) |  | **0.15 (0.08; 0.21)**** |  | 0.09 (-0.03; 0.28) |  | 0.04 (-0.09; 0.19) |  | **0.20 (0.04; 0.23)*** |
| Physical act, kCal/kg/day |  | -0.05 (-0.31; 0.16) |  | 0.01 (-0.04; 0.06) |  | 0.04 (-0.07; 0.17) |  | 0.01 (-0.09; 0.10) |  | 0.01 (-0.08; 0.09) |
| Cotinine, ng/ml |  |  |  | **0.08 (0.02; 0.13)*** |  | 0.05 (-0.07; 0.22) |  | 0.08 (-0.03; 0.18) |  | 0.04 (-0.06; 0.12) |
| GGT, U/L |  | **-0.30 (-0.62; -0.16)**** |  | **-0.24 (-0.30; -0.17)**** |  | **-0.32 (-0.48; -0.23)**** |  | **-0.21 (-0.39; -0.13)**** |  | **-0.17 (-0.24; -0.04)*** |
| 24h Systolic BP, mmHg |  | -0.07 (-0.36; 0.16) |  | 0.01 (-0.06; 0.08) |  | 0.03 (-0.10; 0.17) |  | <0.01 (-0.13; 0.13) |  | -0.03 (-0.13; 0.09) |
| HbA1c, (%) |  | 0.01 (-0.19; 0.22) |  | -0.03 (-0.09; 0.03) |  | -0.06 (-0.19; 0.05) |  | -0.07 (-0.18; 0.04) |  | 0.01 (-0.09; 0.11) |
| LDL cholesterol, mmol/L |  | **-0.33 (-0.81; -0.28)**** |  | **-0.25 (-0.30; -0.18)**** |  | **-0.24 (-0.42; -0.16)**** |  | **-0.25 (-0.39; -0.15)**** |  | **-0.30 (-0.32; -0.13)**** |
| SES score |  | 0.05 (-0.22; 0.41) |  | **-0.11 (-0.17; -0.03)*** |  | -0.08 (-0.24; 0.05) |  | -0.06 (-0.20; 0.08) |  | -0.11 (-0.21; 0.02) |
| Free carnitine, AU | **0.35**** | -0.11 (-0.33; 0.06) | **0.28**** | **0.07 (0.01; 0.13)*** | **0.33**** | 0.07 (-0.03; 0.17) | **0.30**** | 0.06 (-0.04; 0.18) | **0.19**** | 0.09 (-0.02; 0.16) |
| Age, years |  | **-0.20 (-0.43; -0.04)*** |  | **-0.21 (-0.27; -0.14)**** |  | **-0.22 (-0.34; -0.11)**** |  | **-0.21 (-0.35; -0.11)**** |  | **-0.16 (-0.24; -0.03)*** |
| Sex, female/male |  | 0.16 (-0.03; 0.39) |  | 0.06 (-0.01; 0.13) |  | 0.01 (-0.12; 0.14) |  | 0.03 (-0.09; 0.16) |  | 0.10 (-0.03; 0.21) |
| Ethnicity, Black/White |  | -0.10 (-0.36; 0.10) |  | **-0.18 (-0.25; -0.10)**** |  | **-0.27 (-0.41; -0.14)**** |  | **-0.20 (-0.36; -0.07)*** |  | -0.04 (-0.17; 0.10) |
| Protein intake, g |  | -0.05 (-0.23; 0.12) |  | -0.02 (-0.08; 0.04) |  | 0.04 (-0.07; 0.16) |  | -0.04 (-0.15; 0.07) |  | -0.04 (-0.13; 0.06) |
| Waist-to-height ratio |  | 0.14 (-0.02; 0.44) |  | **0.17 (0.10; 0.23)**** |  | 0.10 (-0.01; 0.30) |  | 0.04 (-0.09; 0.20) |  | **0.21 (0.05; 0.24)*** |
| Physical act, kCal/kg/day |  | -0.05 (-0.32; 0.15) |  | 0.01 (-0.05; 0.06) |  | 0.05 (-0.06; 0.18) |  | <0.01 (-0.10; 0.10) |  | 0.01 (-0.08; 0.09) |
| Cotinine, ng/ml |  |  |  | **0.08 (0.02; 0.13)*** |  | 0.04 (-0.08; 0.21) |  | 0.07 (-0.04; 0.18) |  | 0.04 (-0.06; 0.12) |
| GGT, U/L |  | **-0.32 (-0.66; -0.20)**** |  | **-0.24 (-0.31; -0.17)**** |  | **-0.32 (-0.49; -0.23)**** |  | **-0.21 (-0.38; -0.12)**** |  | **-0.17 (-0.25; -0.04)*** |
| 24h Systolic BP, mmHg |  | -0.03 (-0.30; 0.22) |  | 0.02 (-0.05; 0.09) |  | 0.03 (-0.10; 0.17) |  | 0.02 (-0.11; 0.15) |  | -0.03 (-0.14; 0.09) |
| HbA1c, (%) |  | 0.01 (-0.20; 0.23) |  | -0.04 (-0.09; 0.03) |  | -0.06 (-0.19; 0.05) |  | -0.08 (-0.19; 0.03) |  | 0.01 (-0.09; 0.11) |
| LDL cholesterol, mmol/L |  | **-0.37 (-0.88; -0.35)**** |  | **-0.26 (-0.31; -0.18)**** |  | **-0.26 (-0.43; -0.18)**** |  | **-0.26 (-0.40; -0.16)**** |  | **-0.31 (-0.32; -0.13)**** |
| SES score |  | 0.05 (-0.23; 0.41) |  | **-0.09 (-0.16; -0.02)*** |  | -0.07 (-0.22; 0.06) |  | -0.06 (-0.20; 0.08) |  | -0.10 (-0.20; 0.03) |
| Acetylcarnitine, AU | **0.34**** | -0.10 (-0.32; 0.06) | **0.28**** | 0.05 (-0.01; 0.11) | **0.33**** | 0.02 (-0.08; 0.13) | **0.30**** | 0.05 (-0.05; 0.16) | **0.19**** | 0.08 (-0.03; 0.16) |
| Age, years |  | **-0.20 (-0.44; -0.04)*** |  | **-0.21 (-0.26; -0.14)**** |  | **-0.22 (-0.33; -0.11)**** |  | **-0.21 (-0.35; -0.10)**** |  | **-0.16 (-0.24; -0.03)*** |
| Sex, female/male |  | 0.14 (-0.05; 0.37) |  | 0.07 (<0.01; 0.14) |  | 0.02 (-0.10; 0.15) |  | 0.04 (-0.09; 0.17) |  | 0.10 (-0.03; 0.21) |
| Ethnicity, Black/White |  | -0.10 (-0.36; 0.10) |  | **-0.17 (-0.25; -0.09)**** |  | **-0.26 (-0.40; -0.13)**** |  | **-0.20 (-0.35; -0.07)*** |  | -0.04 (-0.17; 0.10) |
| Protein intake, g |  | -0.05 (-0.23; 0.12) |  | -0.02 (-0.08; 0.04) |  | 0.04 (-0.07; 0.15) |  | -0.04 (-0.15; 0.06) |  | -0.05 (-0.13; 0.06) |
| Waist-to-height ratio |  | 0.14 (-0.02; 0.44) |  | **0.17 (0.09; 0.23)**** |  | 0.10 (<0.01; 0.31) |  | 0.04 (-0.09; 0.20) |  | **0.21 (0.05; 0.24)*** |
| Physical act, kCal/kg/day |  | -0.05 (-0.32; 0.15) |  | 0.01 (-0.05; 0.06) |  | 0.05 (-0.06; 0.18) |  | <0.01 (-0.10; 0.10) |  | 0.01 (-0.08; 0.09) |
| Cotinine, ng/ml |  |  |  | **0.08 (0.02; 0.13)*** |  | 0.04 (-0.09; 0.21) |  | 0.07 (-0.04; 0.18) |  | 0.04 (-0.06; 0.12) |
| GGT, U/L |  | **-0.33 (-0.67; -0.20)**** |  | **-0.24 (-0.31; -0.17)**** |  | **-0.32 (-0.49; -0.24)**** |  | **-0.21 (-0.38; -0.12)**** |  | **-0.17 (-0.24; -0.04)*** |
| 24h Systolic BP, mmHg |  | -0.03 (-0.30; 0.22) |  | 0.02 (-0.05; 0.09) |  | 0.03 (-0.10; 0.17) |  | 0.02 (-0.11; 0.15) |  | -0.02 (-0.13; 0.09) |
| HbA1c, (%) |  | 0.01 (-0.20; 0.22) |  | -0.03 (-0.09; 0.03) |  | -0.05 (-0.18; 0.06) |  | -0.08 (-0.19; 0.03) |  | 0.01 (-0.09; 0.11) |
| LDL cholesterol, mmol/L |  | **-0.37 (-0.88; -0.35)**** |  | **-0.26 (-0.31; -0.19)**** |  | **-0.26 (-0.44; -0.18)**** |  | **-0.26 (-0.40; -0.16)**** |  | **-0.32 (-0.33; -0.14)**** |
| SES score |  | 0.05 (-0.22; 0.42) |  | **-0.10 (-0.16; -0.02)*** |  | -0.07 (-0.22; 0.06) |  | -0.06 (-0.20; 0.08) |  | -0.11 (-0.21; 0.03) |

Test used: Multiple linear regressions. Data are presented as adjusted R^2^ with β coefficient and 95% confidence intervals. Estimated glomerular filtration rate (creatinine-based), adjusted for age, sex, ethnicity, protein intake, waist-to-hight ratio, physical activity, cotinine, GGT, 24h systolic BP, HbA1c, LDL, SES score. Bold values denote P≤0.05; *P≤0.05; **P≤0.001. Cardiovascular disease risk group criteria: Obese - ≥0.55 waist-to-height ratio; Physically inactive - <600 METs for moderate and/or vigorous intensity physical activity; Smoking - ≥11 ng/mL cotinine & self-reported smoking; Excessive alcohol intake - ≥49 U/L GGT & self-reported drinking; Masked hypertensive – normal clinic BP & 24h/day/night BP classified as hypertensive; Hyperglycemic - ≥5.7% HbA1c; Dyslipidemic - >3.4 mmol/L LDL; Low socio-economic – low SES.

*Abbreviations*: AU, arbitrary units; physical act, physical activity; GGT, gamma-glutamyl transferase; BP, blood pressure; HbA1c, glycated haemoglobin; LDL, low density lipoprotein; SES, socio-economic status; eGFR, estimated glomerular filtration rate; CVD, cardiovascular disease.

**Supplementary Table 3I. Supplementary analysis with estimated glomerular filtration rate (creatinine-based) as the dependent variable, with the metabolomics data in control, cardiovascular disease risk group and cardiovascular disease risk clusters**

|  | **eGFR (creatinine), ml/min/1.73m^2^** | | | | | | | | | |
| --- | --- | --- | --- | --- | --- | --- | --- | --- | --- | --- |
|  | **Control group**  **(N=166)** | | **CVD risk group**  **(N=1036)** | | **1 CVD risk factor**  **(N=344)** | | **2 CVD risk factors (N=360)** | | **3+ CVD risk factors (N=332)** | |
| ***Metabolomic data*** | **Adj R^2^** | **β (95%Cl)** | **Adj R^2^** | **β (95%Cl)** | **Adj R^2^** | **β (95%Cl)** | **Adj R^2^** | **β (95%Cl)** | **Adj R^2^** | **β (95%Cl)** |
| Propionylcarnitine, AU | **0.34**** | -0.10 (-0.31; 0.07) | **0.28**** | **0.07 (0.01; 0.13)*** | **0.33**** | 0.06 (-0.05; 0.16) | **0.29**** | 0.05 (-0.06; 0.17) | **0.19**** | 0.12 (<0.01; 0.19) |
| Age, years |  | **-0.21 (-0.45; -0.04)*** |  | **-0.20 (-0.27; -0.13)**** |  | **-0.22 (-0.34; -0.10)**** |  | **-0.21 (-0.35; -0.09)**** |  | **-0.16 (-0.24; -0.02)*** |
| Sex, female/male |  | 0.17 (-0.03; 0.42) |  | 0.06 (-0.02; 0.13) |  | 0.01 (-0.12; 0.14) |  | 0.04 (-0.10; 0.17) |  | 0.09 (-0.04; 0.21) |
| Ethnicity, Black/White |  | -0.11 (-0.38; 0.10) |  | **-0.17 (-0.25; -0.09)**** |  | **-0.26 (-0.41; -0.13)**** |  | **-0.19 (-0.35; -0.06)*** |  | -0.05 (-0.19; 0.10) |
| Protein intake, g |  | -0.06 (-0.25; 0.11) |  | -0.02 (-0.09; 0.04) |  | 0.04 (-0.08; 0.16) |  | -0.04 (-0.16; 0.07) |  | -0.05 (-0.14; 0.06) |
| Waist-to-height ratio |  | 0.13 (-0.04; 0.42) |  | **0.17 (0.09; 0.23)**** |  | 0.10 (-0.02; 0.31) |  | 0.04 (-0.10; 0.21) |  | **0.22 (0.05; 0.25)*** |
| Physical act, kCal/kg/day |  | -0.05 (-0.33; 0.16) |  | 0.01 (-0.05; 0.07) |  | 0.05 (-0.06; 0.19) |  | <0.01 (-0.10; 0.11) |  | 0.01 (-0.08; 0.09) |
| Cotinine, ng/ml |  |  |  | **0.08 (0.02; 0.13)*** |  | 0.04 (-0.09; 0.22) |  | 0.07 (-0.04; 0.18) |  | 0.04 (-0.06; 0.13) |
| GGT, U/L |  | **-0.32 (-0.67; -0.19)**** |  | **-0.24 (-0.31; -0.17)**** |  | **-0.32 (-0.50; -0.23)**** |  | **-0.21 (-0.39; -0.11)**** |  | **-0.18 (-0.26; -0.04)*** |
| 24h Systolic BP, mmHg |  | -0.03 (-0.32; 0.23) |  | 0.02 (-0.06; 0.09) |  | 0.03 (-0.11; 0.18) |  | 0.02 (-0.12; 0.16) |  | -0.03 (-0.14; 0.09) |
| HbA1c, (%) |  | 0.01 (-0.20; 0.24) |  | -0.04 (-0.10; 0.03) |  | -0.06 (-0.19; 0.06) |  | -0.08 (-0.20; 0.03) |  | 0.01 (-0.10; 0.11) |
| LDL cholesterol, mmol/L |  | **-0.37 (-0.89; -0.33)**** |  | **-0.26 (-0.31; -0.18)**** |  | **-0.26 (-0.44; -0.17)**** |  | **-0.26 (-0.41; -0.16)**** |  | **-0.31 (-0.32; -0.12)**** |
| SES score |  | 0.05 (-0.23; 0.43) |  | **-0.09 (-0.17; -0.02)*** |  | -0.06 (-0.23; 0.07) |  | -0.06 (-0.21; 0.09) |  | -0.10 (-0.21; 0.04) |
| Butyrylcarnitine, AU | **0.34**** | 0.01 (-0.16; 0.19) | **0.28**** | **0.06 (<0.01; 0.11)*** | **0.34**** | 0.09 (-0.01; 0.19) | **0.30**** | 0.06 (-0.05; 0.18) | **0.19**** | 0.03 (-0.07; 0.11) |
| Age, years |  | **-0.22 (-0.45; -0.06)*** |  | **-0.21 (-0.26; -0.14)**** |  | **-0.22 (-0.33; -0.11)**** |  | **-0.21 (-0.35; -0.10)**** |  | **-0.15 (-0.23; -0.02)*** |
| Sex, female/male |  | 0.14 (-0.05; 0.37) |  | 0.07 (<0.01; 0.14) |  | 0.02 (-0.11; 0.14) |  | 0.04 (-0.08; 0.17) |  | 0.11 (-0.02; 0.22) |
| Ethnicity, Black/White |  | -0.14 (-0.40; 0.05) |  | **-0.17 (-0.24; -0.09)**** |  | **-0.27 (-0.41; -0.14)**** |  | **-0.20 (-0.35; -0.07)*** |  | -0.04 (-0.17; 0.10) |
| Protein intake, g |  | -0.07 (-0.25; 0.10) |  | -0.02 (-0.08; 0.04) |  | 0.04 (-0.07; 0.15) |  | -0.05 (-0.16; 0.06) |  | -0.05 (-0.14; 0.06) |
| Waist-to-height ratio |  | 0.11 (-0.06; 0.39) |  | **0.17 (0.10; 0.23)**** |  | 0.10 (<0.01; 0.31) |  | 0.04 (-0.09; 0.20) |  | **0.21 (0.05; 0.23)*** |
| Physical act, kCal/kg/day |  | -0.04 (-0.31; 0.17) |  | 0.01 (-0.05; 0.06) |  | 0.04 (-0.07; 0.17) |  | <0.01 (-0.10; 0.10) |  | 0.01 (-0.08; 0.09) |
| Cotinine, ng/ml |  |  |  | **0.08 (0.02; 0.13)*** |  | 0.05 (-0.08; 0.22) |  | 0.07 (-0.04; 0.18) |  | 0.04 (-0.06; 0.12) |
| GGT, U/L |  | **-0.31 (-0.65; -0.18)**** |  | **-0.24 (-0.30; -0.17)**** |  | **-0.32 (-0.49; -0.23)**** |  | **-0.21 (-0.39; -0.12)**** |  | **-0.16 (-0.24; -0.04)*** |
| 24h Systolic BP, mmHg |  | -0.04 (-0.32; 0.21) |  | 0.01 (-0.06; 0.08) |  | 0.04 (-0.09; 0.17) |  | 0.01 (-0.12; 0.15) |  | -0.03 (-0.13; 0.09) |
| HbA1c, (%) |  | 0.01 (-0.20; 0.23) |  | -0.04 (-0.10; 0.02) |  | -0.06 (-0.19; 0.05) |  | -0.08 (-0.19; 0.03) |  | <0.01 (-0.10; 0.10) |
| LDL cholesterol, mmol/L |  | **-0.35 (-0.85; -0.31)**** |  | **-0.26 (-0.31; -0.18)**** |  | **-0.25 (-0.42; -0.17)**** |  | **-0.26 (-0.40; -0.16)**** |  | **-0.31 (-0.32; -0.13)**** |
| SES score |  | 0.06 (-0.21; 0.43) |  | **-0.09 (-0.16; -0.02)*** |  | -0.07 (-0.23; 0.05) |  | -0.05 (-0.20; 0.09) |  | -0.10 (-0.20; 0.04) |
| Isovalerylcarnitine, AU | **0.34**** | -0.07 (-0.29; 0.11) | **0.28**** | **0.09 (0.03; 0.15)*** | **0.34**** | 0.09 (-0.02; 0.19) | **0.30**** | 0.10 (<0.01; 0.21) | **0.19**** | 0.08 (-0.03; 0.17) |
| Age, years |  | **-0.21 (-0.44; -0.04)*** |  | **-0.20 (-0.26; -0.13)**** |  | **-0.21 (-0.33; -0.11)**** |  | **-0.21 (-0.35; -0.11)**** |  | **-0.15 (-0.23; -0.02)*** |
| Sex, female/male |  | 0.15 (-0.04; 0.39) |  | 0.05 (-0.02; 0.12) |  | <0.01 (-0.12; 0.13) |  | 0.02 (-0.10; 0.15) |  | 0.10 (-0.03; 0.21) |
| Ethnicity, Black/White |  | -0.11 (-0.38; 0.09) |  | **-0.18 (-0.25; -0.10)**** |  | **-0.27 (-0.41; -0.15)**** |  | **-0.21 (-0.37; -0.09)**** |  | -0.05 (-0.18; 0.09) |
| Protein intake, g |  | -0.05 (-0.23; 0.12) |  | -0.02 (-0.08; 0.04) |  | 0.04 (-0.07; 0.15) |  | -0.04 (-0.16; 0.06) |  | -0.04 (-0.13; 0.07) |
| Waist-to-height ratio |  | 0.12 (-0.05; 0.40) |  | **0.16 (0.09; 0.22)**** |  | 0.09 (-0.02; 0.29) |  | 0.04 (-0.10; 0.19) |  | **0.20 (0.04; 0.23)*** |
| Physical act, kCal/kg/day |  | -0.04 (-0.3; 0.17) |  | 0.01 (-0.05; 0.06) |  | 0.05 (-0.06; 0.18) |  | <0.01 (-0.10; 0.10) |  | <0.01 (-0.08; 0.09) |
| Cotinine, ng/ml |  |  |  | **0.08 (0.02; 0.13)*** |  | 0.04 (-0.08; 0.21) |  | 0.07 (-0.03; 0.18) |  | 0.03 (-0.07; 0.11) |
| GGT, U/L |  | **-0.31 (-0.65; -0.18)**** |  | **-0.24 (-0.30; -0.17)**** |  | **-0.32 (-0.49; -0.23)**** |  | **-0.21 (-0.39; -0.12)**** |  | **-0.17 (-0.24; -0.04)*** |
| 24h Systolic BP, mmHg |  | -0.03 (-0.31; 0.22) |  | 0.01 (-0.06; 0.08) |  | 0.03 (-0.10; 0.17) |  | 0.01 (-0.12; 0.14) |  | -0.03 (-0.14; 0.09) |
| HbA1c, (%) |  | 0.01 (-0.19; 0.23) |  | -0.04 (-0.10; 0.03) |  | -0.05 (-0.18; 0.06) |  | -0.08 (-0.19; 0.03) |  | 0.01 (-0.10; 0.10) |
| LDL cholesterol, mmol/L |  | **-0.38 (-0.90; -0.35)**** |  | **-0.26 (-0.31; -0.19)**** |  | **-0.25 (-0.43; -0.18)**** |  | **-0.26 (-0.40; -0.16)**** |  | **-0.32 (-0.33; -0.14)**** |
| SES score |  | 0.06 (-0.21; 0.43) |  | **-0.10 (-0.17; -0.03)*** |  | -0.07 (-0.23; 0.06) |  | -0.06 (-0.20; 0.08) |  | -0.11 (-0.21; 0.03) |
| Hexanoylcarnitine, AU | **0.33**** | 0.02 (-0.16; 0.21) | **0.28**** | **0.09 (0.03; 0.14)*** | **0.33**** | 0.06 (-0.03; 0.16) | **0.31**** | **0.12 (0.02; 0.25)*** | **0.19**** | 0.06 (-0.04; 0.14) |
| Age, years |  | **-0.22 (-0.46; -0.05)*** |  | **-0.21 (-0.27; -0.14)**** |  | **-0.22 (-0.34; -0.11)**** |  | **-0.21 (-0.35; -0.11)**** |  | **-0.16 (-0.24; -0.03)*** |
| Sex, female/male |  | 0.14 (-0.06; 0.38) |  | **0.07 (<0.01; 0.14)*** |  | 0.02 (-0.1; 0.15) |  | 0.05 (-0.07; 0.19) |  | 0.11 (-0.02; 0.22) |
| Ethnicity, Black/White |  | -0.14 (-0.41; 0.05) |  | **-0.17 (-0.24; -0.09)**** |  | **-0.25 (-0.39; -0.13)**** |  | **-0.21 (-0.36; -0.08)*** |  | -0.04 (-0.17; 0.10) |
| Protein intake, g |  | -0.06 (-0.25; 0.11) |  | -0.01 (-0.07; 0.05) |  | 0.04 (-0.07; 0.16) |  | -0.03 (-0.14; 0.08) |  | -0.04 (-0.13; 0.06) |
| Waist-to-height ratio |  | 0.11 (-0.06; 0.40) |  | **0.17 (0.09; 0.23)**** |  | **0.10 (<0.01; 0.31)*** |  | 0.04 (-0.09; 0.20) |  | **0.21 (0.05; 0.24)*** |
| Physical act, kCal/kg/day |  | -0.04 (-0.31; 0.18) |  | 0.01 (-0.05; 0.06) |  | 0.05 (-0.06; 0.18) |  | <0.01 (-0.10; 0.10) |  | 0.01 (-0.08; 0.09) |
| Cotinine, ng/ml |  |  |  | **0.07 (0.01; 0.12)*** |  | 0.04 (-0.08; 0.22) |  | 0.06 (-0.05; 0.17) |  | 0.03 (-0.07; 0.11) |
| GGT, U/L |  | **-0.31 (-0.66; -0.18)**** |  | **-0.23 (-0.30; -0.16)**** |  | **-0.31 (-0.48; -0.22)**** |  | **-0.21 (-0.39; -0.12)**** |  | **-0.16 (-0.24; -0.03)*** |
| 24h Systolic BP, mmHg |  | -0.04 (-0.33; 0.21) |  | 0.01 (-0.06; 0.08) |  | 0.03 (-0.10; 0.17) |  | 0.01 (-0.12; 0.15) |  | -0.03 (-0.13; 0.09) |
| HbA1c, (%) |  | 0.01 (-0.21; 0.23) |  | -0.04 (-0.10; 0.02) |  | -0.06 (-0.19; 0.06) |  | -0.09 (-0.20; 0.02) |  | 0.01 (-0.10; 0.10) |
| LDL cholesterol, mmol/L |  | **-0.35 (-0.86; -0.30)**** |  | **-0.26 (-0.31; -0.18)**** |  | **-0.26 (-0.43; -0.18)**** |  | **-0.26 (-0.40; -0.16)**** |  | **-0.31 (-0.32; -0.13)**** |
| SES score |  | 0.06 (-0.22; 0.44) |  | **-0.09 (-0.16; -0.01)*** |  | -0.07 (-0.23; 0.06) |  | -0.03 (-0.17; 0.11) |  | -0.10 (-0.20; 0.04) |

Test used: Multiple linear regressions. Data are presented as adjusted R^2^ with β coefficient and 95% confidence intervals. Estimated glomerular filtration rate (creatinine-based), adjusted for age, sex, ethnicity, protein intake, waist-to-hight ratio, physical activity, cotinine, GGT, 24h systolic BP, HbA1c, LDL, SES score. Bold values denote P≤0.05; *P≤0.05; **P≤0.001. Cardiovascular disease risk group criteria: Obese - ≥0.55 waist-to-height ratio; Physically inactive - <600 METs for moderate and/or vigorous intensity physical activity; Smoking - ≥11 ng/mL cotinine & self-reported smoking; Excessive alcohol intake - ≥49 U/L GGT & self-reported drinking; Masked hypertensive – normal clinic BP & 24h/day/night BP classified as hypertensive; Hyperglycemic - ≥5.7% HbA1c; Dyslipidemic - >3.4 mmol/L LDL; Low socio-economic – low SES.

*Abbreviations*: AU, arbitrary units; physical act, physical activity; GGT, gamma-glutamyl transferase; BP, blood pressure; HbA1c, glycated haemoglobin; LDL, low density lipoprotein; SES, socio-economic status; eGFR, estimated glomerular filtration rate; CVD, cardiovascular disease.

**Supplementary Table 3J. Supplementary analysis with estimated glomerular filtration rate (creatinine-based) as the dependent variable, with the metabolomics data in control, cardiovascular disease risk group and cardiovascular disease risk clusters**

|  | **eGFR (creatinine), ml/min/1.73m^2^** | | | | | | | | | |
| --- | --- | --- | --- | --- | --- | --- | --- | --- | --- | --- |
|  | **Control group**  **(N=166)** | | **CVD risk group**  **(N=1036)** | | **1 CVD risk factor**  **(N=344)** | | **2 CVD risk factors (N=360)** | | **3+ CVD risk factors (N=332)** | |
| ***Metabolomic data*** | **Adj R^2^** | **β (95%Cl)** | **Adj R^2^** | **β (95%Cl)** | **Adj R^2^** | **β (95%Cl)** | **Adj R^2^** | **β (95%Cl)** | **Adj R^2^** | **β (95%Cl)** |
| Octanoylcarnitine, AU | **0.34**** | <0.01 (-0.20; 0.20) | **0.28**** | **0.09 (0.04; 0.15)**** | **0.33**** | 0.06 (-0.04; 0.17) | **0.31**** | **0.12 (0.02; 0.24)*** | **0.19**** | 0.07 (-0.03; 0.14) |
| Age, years |  | **-0.22 (-0.46; -0.05)*** |  | **-0.21 (-0.27; -0.14)**** |  | **-0.22 (-0.34; -0.11)**** |  | **-0.22 (-0.35; -0.11)**** |  | **-0.16 (-0.23; -0.03)*** |
| Sex, female/male |  | 0.14 (-0.05; 0.38) |  | 0.06 (-0.01; 0.13) |  | 0.02 (-0.11; 0.14) |  | 0.03 (-0.09; 0.16) |  | 0.11 (-0.02; 0.21) |
| Ethnicity, Black/White |  | -0.13 (-0.40; 0.05) |  | **-0.17 (-0.25; -0.10)**** |  | **-0.26 (-0.40; -0.13)**** |  | **-0.22 (-0.38; -0.09)**** |  | -0.04 (-0.17; 0.10) |
| Protein intake, g |  | -0.06 (-0.25; 0.10) |  | -0.01 (-0.07; 0.05) |  | 0.04 (-0.07; 0.16) |  | -0.03 (-0.14; 0.08) |  | -0.04 (-0.13; 0.07) |
| Waist-to-height ratio |  | 0.11 (-0.06; 0.39) |  | **0.16 (0.09; 0.22)**** |  | 0.10 (<0.01; 0.31) |  | 0.04 (-0.09; 0.20) |  | **0.21 (0.05; 0.24)*** |
| Physical act, kCal/kg/day |  | -0.04 (-0.31; 0.17) |  | 0.01 (-0.05; 0.06) |  | 0.05 (-0.06; 0.18) |  | <0.01 (-0.10; 0.09) |  | 0.01 (-0.08; 0.09) |
| Cotinine, ng/ml |  |  |  | **0.08 (0.02; 0.13)*** |  | 0.04 (-0.08; 0.21) |  | 0.07 (-0.04; 0.18) |  | 0.04 (-0.06; 0.12) |
| GGT, U/L |  | **-0.31 (-0.65; -0.18)**** |  | **-0.23 (-0.30; -0.16)**** |  | **-0.31 (-0.48; -0.22)**** |  | **-0.21 (-0.38; -0.12)**** |  | **-0.16 (-0.24; -0.04)*** |
| 24h Systolic BP, mmHg |  | -0.04 (-0.32; 0.21) |  | 0.01 (-0.06; 0.08) |  | 0.03 (-0.10; 0.16) |  | 0.02 (-0.11; 0.15) |  | -0.03 (-0.14; 0.09) |
| HbA1c, (%) |  | 0.01 (-0.20; 0.23) |  | -0.04 (-0.09; 0.03) |  | -0.05 (-0.18; 0.06) |  | -0.08 (-0.19; 0.03) |  | 0.01 (-0.10; 0.10) |
| LDL cholesterol, mmol/L |  | **-0.36 (-0.86; -0.32)**** |  | **-0.26 (-0.31; -0.18)**** |  | **-0.26 (-0.43; -0.18)**** |  | **-0.25 (-0.39; -0.15)**** |  | **-0.31 (-0.32; -0.13)**** |
| SES score |  | 0.06 (-0.21; 0.44) |  | **-0.09 (-0.16; -0.02)*** |  | -0.07 (-0.22; 0.06) |  | -0.04 (-0.18; 0.10) |  | -0.10 (-0.20; 0.03) |
| Decanoylcarnitine, AU | **0.34**** | 0.01 (-0.17; 0.19) | **0.29**** | **0.11 (0.05; 0.16)**** | **0.33**** | 0.07 (-0.03; 0.18) | **0.31**** | **0.12 (0.02; 0.23)*** | **0.20**** | **0.12 (<0.01; 0.18)*** |
| Age, years |  | **-0.22 (-0.45; -0.06)*** |  | **-0.20 (-0.26; -0.14)**** |  | **-0.22 (-0.33; -0.11)**** |  | **-0.21 (-0.34; -0.10)**** |  | **-0.15 (-0.23; -0.02)*** |
| Sex, female/male |  | 0.14 (-0.05; 0.37) |  | 0.06 (-0.01; 0.13) |  | 0.01 (-0.11; 0.14) |  | 0.03 (-0.10; 0.16) |  | 0.11 (-0.03; 0.21) |
| Ethnicity, Black/White |  | -0.14 (-0.40; 0.05) |  | **-0.17 (-0.24; -0.09)**** |  | **-0.26 (-0.40; -0.13)**** |  | **-0.22 (-0.37; -0.09)**** |  | -0.03 (-0.16; 0.10) |
| Protein intake, g |  | -0.06 (-0.25; 0.10) |  | -0.01 (-0.07; 0.05) |  | 0.04 (-0.07; 0.16) |  | -0.02 (-0.14; 0.08) |  | -0.05 (-0.13; 0.06) |
| Waist-to-height ratio |  | 0.11 (-0.06; 0.39) |  | **0.17 (0.10; 0.23)**** |  | **0.10 (<0.01; 0.31)*** |  | 0.05 (-0.08; 0.21) |  | **0.21 (0.05; 0.24)*** |
| Physical act, kCal/kg/day |  | -0.04 (-0.3; 0.17) |  | <0.01 (-0.05; 0.06) |  | 0.05 (-0.06; 0.18) |  | <0.01 (-0.10; 0.09) |  | <0.01 (-0.08; 0.08) |
| Cotinine, ng/ml |  |  |  | **0.08 (0.02; 0.13)*** |  | 0.05 (-0.08; 0.22) |  | 0.07 (-0.03; 0.18) |  | 0.04 (-0.06; 0.12) |
| GGT, U/L |  | **-0.31 (-0.65; -0.18)**** |  | **-0.23 (-0.30; -0.16)**** |  | **-0.31 (-0.48; -0.22)**** |  | **-0.20 (-0.38; -0.12)**** |  | **-0.16 (-0.24; -0.03)*** |
| 24h Systolic BP, mmHg |  | -0.04 (-0.32; 0.21) |  | 0.01 (-0.06; 0.08) |  | 0.02 (-0.11; 0.16) |  | 0.02 (-0.11; 0.15) |  | -0.03 (-0.14; 0.09) |
| HbA1c, (%) |  | 0.01 (-0.20; 0.23) |  | -0.03 (-0.09; 0.03) |  | -0.05 (-0.18; 0.06) |  | -0.08 (-0.19; 0.02) |  | 0.01 (-0.09; 0.11) |
| LDL cholesterol, mmol/L |  | **-0.35 (-0.86; -0.31)**** |  | **-0.26 (-0.31; -0.18)**** |  | **-0.26 (-0.43; -0.18)**** |  | **-0.26 (-0.40; -0.16)**** |  | **-0.31 (-0.32; -0.13)**** |
| SES score |  | 0.06 (-0.21; 0.43) |  | **-0.09 (-0.16; -0.02)*** |  | -0.07 (-0.23; 0.06) |  | -0.04 (-0.19; 0.09) |  | -0.11 (-0.21; 0.02) |
| Dodecanoylcarnitine, AU | 0.12 | -0.09 (-0.56; 0.34) | **0.25**** | -0.04 (-0.15; 0.07) | **0.24*** | -0.04 (-0.23; 0.16) | **0.19*** | 0.08 (-0.20; 0.42) | 0.10 | -0.18 (-0.31; 0.05) |
| Age, years |  | -0.19 (-0.68; 0.24) |  | **-0.2 (-0.32; -0.07)*** |  | -0.21 (-0.44; 0.01) |  | -0.19 (-0.48; 0.07) |  | -0.13 (-0.31; 0.10) |
| Sex, female/male |  | 0.11 (-0.37; 0.62) |  | 0.07 (-0.07; 0.20) |  | 0.02 (-0.23; 0.27) |  | 0.06 (-0.22; 0.34) |  | 0.09 (-0.16; 0.31) |
| Ethnicity, Black/White |  | -0.15 (-0.71; 0.31) |  | **-0.16 (-0.31; -0.01)*** |  | -0.25 (-0.53; 0.01) |  | -0.18 (-0.50; 0.11) |  | -0.06 (-0.32; 0.21) |
| Protein intake, g |  | -0.04 (-0.45; 0.35) |  | -0.02 (-0.14; 0.09) |  | 0.04 (-0.19; 0.27) |  | -0.03 (-0.27; 0.21) |  | -0.08 (-0.26; 0.13) |
| Waist-to-height ratio |  | 0.12 (-0.32; 0.69) |  | **0.16 (0.03; 0.28)*** |  | 0.10 (-0.17; 0.47) |  | 0.04 (-0.27; 0.37) |  | 0.16 (-0.08; 0.30) |
| Physical act, kCal/kg/day |  | -0.05 (-0.62; 0.45) |  | 0.02 (-0.09; 0.12) |  | 0.05 (-0.18; 0.31) |  | -0.01 (-0.23; 0.21) |  | 0.03 (-0.14; 0.19) |
| Cotinine, ng/ml |  |  |  | 0.07 (-0.04; 0.18) |  | 0.03 (-0.25; 0.35) |  | 0.04 (-0.20; 0.29) |  | 0.04 (-0.15; 0.20) |
| GGT, U/L |  | -0.31 (-0.94; 0.10) |  | **-0.24 (-0.37; -0.11)**** |  | **-0.33 (-0.63; -0.11)*** |  | -0.20 (-0.53; 0.05) |  | -0.20 (-0.37; 0.04) |
| 24h Systolic BP, mmHg |  | -0.03 (-0.63; 0.55) |  | 0.03 (-0.11; 0.17) |  | 0.04 (-0.24; 0.32) |  | 0.01 (-0.28; 0.31) |  | 0.05 (-0.19; 0.27) |
| HbA1c, (%) |  | -0.02 (-0.54; 0.48) |  | -0.04 (-0.16; 0.08) |  | -0.06 (-0.33; 0.18) |  | -0.07 (-0.32; 0.16) |  | <0.01 (-0.20; 0.19) |
| LDL cholesterol, mmol/L |  | -0.37 (-1.20; -0.01)* |  | **-0.26 (-0.36; -0.12)**** |  | **-0.27 (-0.58; -0.06)*** |  | **-0.27 (-0.56; -0.03)*** |  | -0.25 (-0.38; 0.01) |
| SES score |  | 0.06 (-0.60; 0.83) |  | -0.09 (-0.23; 0.05) |  | -0.06 (-0.37; 0.21) |  | -0.06 (-0.37; 0.25) |  | -0.10 (-0.31; 0.15) |

Test used: Multiple linear regressions. Data are presented as adjusted R^2^ with β coefficient and 95% confidence intervals. Estimated glomerular filtration rate (creatinine-based), adjusted for age, sex, ethnicity, protein intake, waist-to-hight ratio, physical activity, cotinine, GGT, 24h systolic BP, HbA1c, LDL, SES score. Bold values denote P≤0.05; *P≤0.05; **P≤0.001. Cardiovascular disease risk group criteria: Obese - ≥0.55 waist-to-height ratio; Physically inactive - <600 METs for moderate and/or vigorous intensity physical activity; Smoking - ≥11 ng/mL cotinine & self-reported smoking; Excessive alcohol intake - ≥49 U/L GGT & self-reported drinking; Masked hypertensive – normal clinic BP & 24h/day/night BP classified as hypertensive; Hyperglycemic - ≥5.7% HbA1c; Dyslipidemic - >3.4 mmol/L LDL; Low socio-economic – low SES.

*Abbreviations*: AU, arbitrary units; physical act, physical activity; GGT, gamma-glutamyl transferase; BP, blood pressure; HbA1c, glycated haemoglobin; LDL, low density lipoprotein; SES, socio-economic status; eGFR, estimated glomerular filtration rate; CVD, cardiovascular disease.
